# Synthesis, Reactivity, and Bonding Analysis of a Tetracoordinated Nickel Carbene

**DOI:** 10.1002/chem.202403211

**Published:** 2024-10-23

**Authors:** Pablo M. Pérez‐García, María L. G. Sansores‐Paredes, Célia Fonseca Guerra, Pascal Vermeeren, Marc‐Etienne Moret

**Affiliations:** ^1^ Organic Chemistry and Catalysis, Institute for Sustainable and Circular Chemistry Utrecht University Universiteitsweg 99 3584 CG Utrecht The Netherlands; ^2^ Department of Chemistry and Pharmaceutical Sciences AIMMS Vrije Universiteit Amsterdam De Boelelaan 1108 1081 HZ Amsterdam The Netherlands; ^3^ Universidad Privada Boliviana (UPB) Cochabamba Bolivia

**Keywords:** metal carbenes, nickel, bonding analysis, carbene transfer, metal-ligand multiple bonds

## Abstract

Nickel carbenes are key reactive intermediates in the catalytic cyclopropanation of olefins and other reactions, but isolated examples are scarce and generally rely on low coordination numbers (≤3) to stabilize the metal−ligand multiple bond. Here we report the isolation and characterization of a stable tetracoordinated nickel carbene bearing a triphosphine pincer ligand. Its nucleophilic character is evidenced by reaction with acids, and it can transfer the carbene fragment to CO to form a ketene. A computational study of the Ni=C chemical bond sheds light on the role of the third phosphine in the pincer framework to the stabilization of the nickel carbene fragment.

## Graphical abstract

The isolation of a four‐coordinate triphosphine nickel carbene complex challenges the generally accepted idea that low coordination numbers are required to stabilize M=C double bonds at late transition metal centers. The isolated compound displays nucleophilic and carbene‐transfer reactivity. Computational analyses shed light on the unexpected role of the third phosphine in stabilizing the Ni=C fragment.

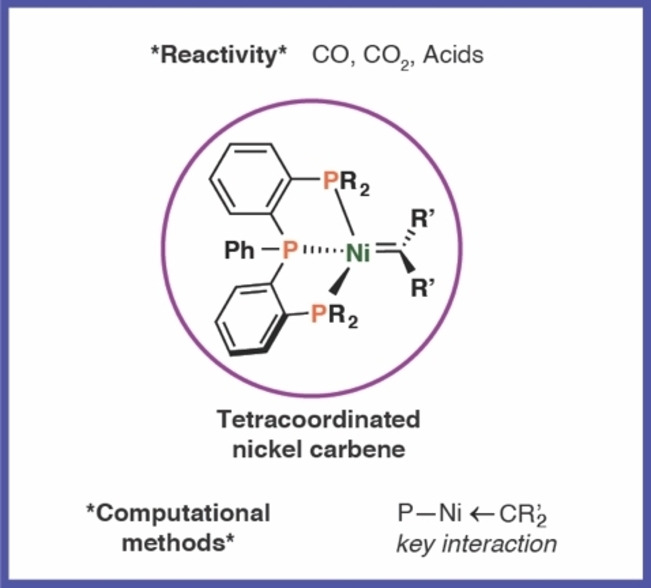

## Introduction

Metal carbenes are important intermediates for many broadly applied chemical reactions such as cyclopropanations,^[1]^ cross‐couplings,^[2]^ and olefin metathesis.^[3]^ A detailed understanding of the reactivity of these organometallic entities is necessary to fully exploit their catalytic potential, both to optimize known reactions and to develop new ones. First row transition metal centers are of high current interest, because of not only their earth‐abundance but also their high reactivity.[^4^, ^5^]

**Figure 1:**
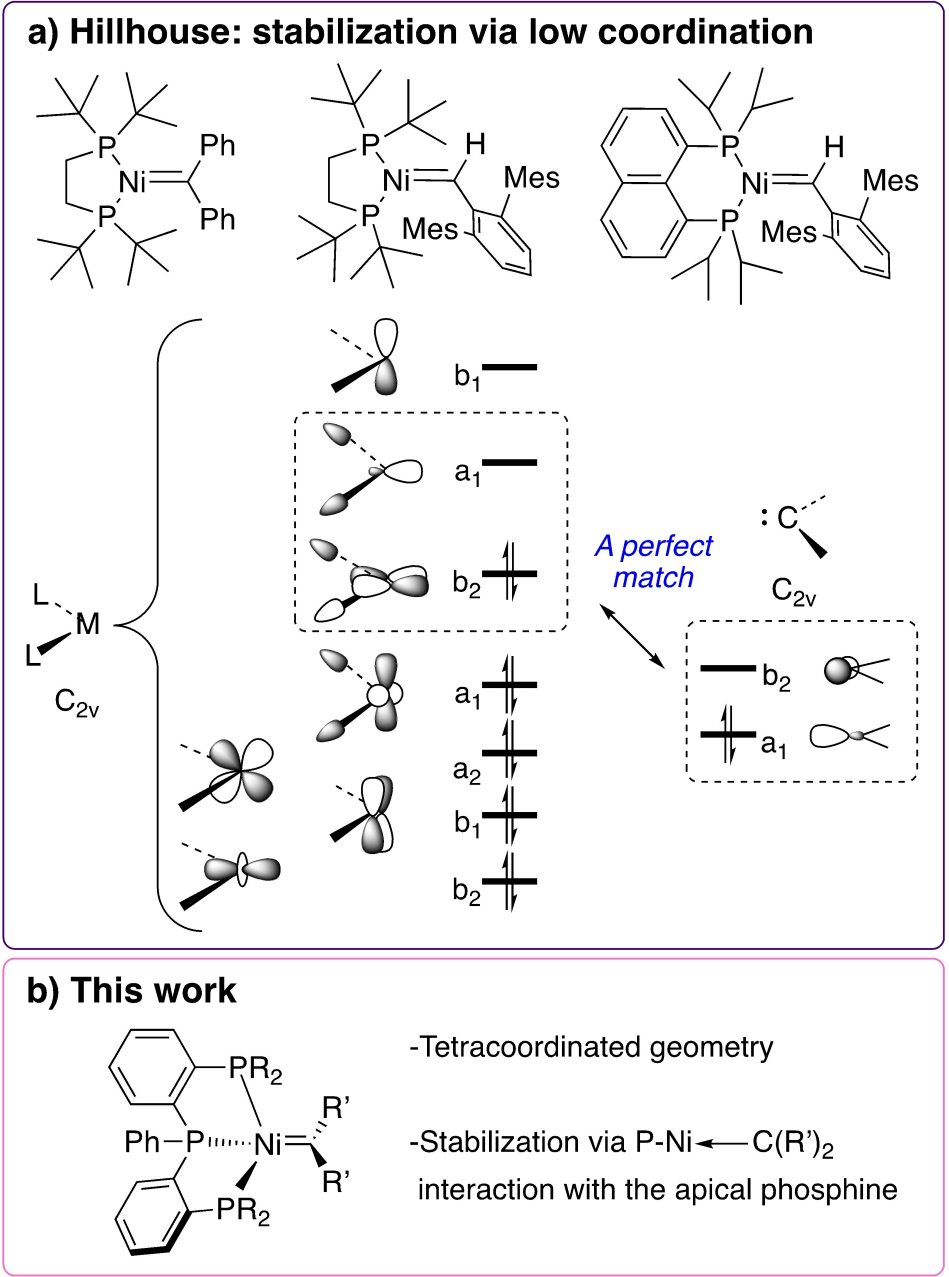
a) Nickel carbenes reported by Hillhouse,[^8^, ^9^, ^13^] b) tetracoordinated nickel carbene presented in this work.

Early work by Miyashita and Grubbs[^6^, ^7^] suggested the potential of nickel carbenes generated by the fragmentation of metallacycles for olefin metathesis and cyclopropanation. However, the stabilization of such species is challenging because of the electron rich nature of nickel and the strong σ‐donation of the carbene fragment. Several years later, based on the conceptualization that a “L_2_Ni” moiety has isolobal orbitals with the CH_2_ fragment, the group of Hillhouse pioneered the synthetic study of nickel carbene species.[^8^, ^9^] They described the isolation and thorough characterization of 16 valence electrons nickel carbene complexes bearing the dtpbe ligand (bis(di‐tert‐butylphosphino)ethane=dtbpe) and another related rigid bidentate ligand (Figure 1). To the best of our knowledge these are the only examples of isolated nickel carbenes that are neither heteroatom‐stabilized (*e. g*., NHCs)^[10]^ nor incorporated in a multidentate (*e. g*., pincer) ligand.^[11]^

The dtbpe‐supported nickel carbene complex undergoes nucleophilic addition to two molecules of CO_2_ to form an 1,1‐dicarboxylate ligand, and it can transfer the carbene fragment to small molecules such as CO and ethylene to form the corresponding ketene and cyclopropane, respectively.^[8]^ In the case of the reaction with ethylene, a metallacyclobutane intermediate was proposed.^[12]^

Our group recently investigated the reactivity of pentacoordinated nickelacyclobutanes with distorted trigonal bipyramidal geometry incorporated in a pincer framework.^[14]^ In agreement with the early observation from Myashita that the metathesis‐like fragmentation of nickelacyclobutanes could be favored by higher coordination numbers,^[15]^ selective [2+2] cycloreversion could be observed in one case. This contrasts with the known reactivity of square planar nickelacyclobutanes, for which no metathesis‐like fragmentation has been observed. For a putative olefin‐metathesis cycle based on a 5‐coordinate nickelacyclobutane to be viable, the corresponding 4‐coordinate, 18 valence electrons Ni carbene would also need to be stable despite deviating from the isolobal paradigm outlined above.

In this work, we investigate whether such a species can be supported by a tridentate phosphine ligand. We present the synthesis of a well‐defined tetracoordinated nickel carbene complex bearing a triphosphine pincer ligand and study its reactivity. DFT calculations,[^16^, ^17^, ^18^] the Activation Strain Model (ASM)[^19^, ^20^] and Energy Decomposition Analyses (EDA) within the framework of quantitative Kohn–Sham molecular orbital theory[^21^, ^22^] are used to shine light onto the bonding situation of the tetracoordinated nickel carbene.

## Results and Discussion

**Figure 2:**
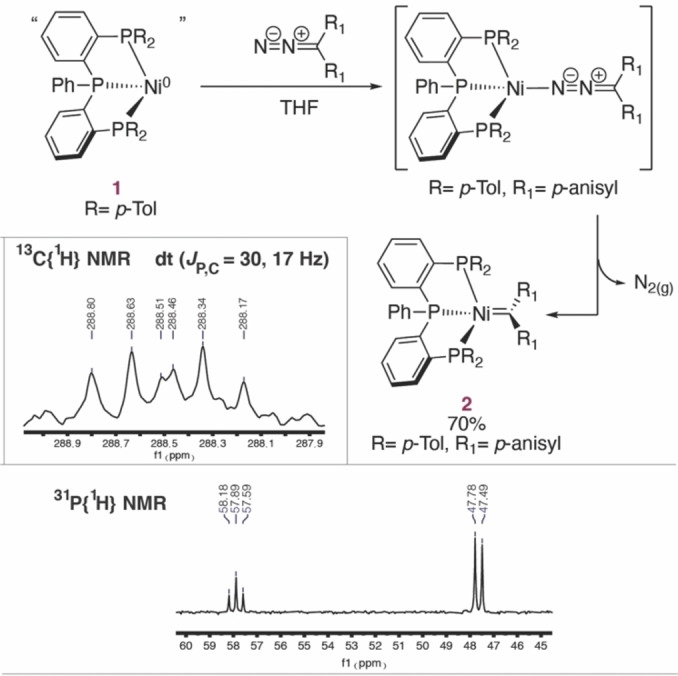
Synthesis of nickel carbene **2** bearing pincer ligand PPP^*p‐tol*^ and relevant NMR spectra.

Reduction of the previously reported PPP^*p*−tol^Ni^I^Br complex^[23]^ with sodium naphthalenide afforded well‐defined Ni(0) complex **1** bearing only nitrogen as coligand (Figure 2 and Figures S1–S5). Treating complex **1** with 1 equivalent of N_2_C(*p*‐anisyl)_2_ resulted in instantaneous complete conversion to two new species detected by ^31^P{^1^H} NMR analysis (Figure S6). The IR spectrum of the reaction mixture showed a strong signal at 2030 cm^−1^, supporting the presence of a Ni(0) diazo complex as major species (Figure S7), which is associated with broad ^31^P NMR signals at 20 and 75 ppm. With the help of DFT calculations, we propose the diazoalkane to be coordinated in η^1^(N) mode to the nickel center to be the most stable coordination mode (Figure S34), in line with other structurally characterized tetracoordinated Ni(0) diazo compounds.[^14^, ^24^] This species cleanly converted into the initially minor product **2** over five days at room temperature. The latter displayed a clear triplet and doublet at 57.9 and 47.6 ppm in ^31^P NMR (Figure 2). Gratifyingly, ^13^C{^1^H} NMR analysis revealed that compound **2** is a Ni‐carbene complex with a diagnostic signal at 288.8 ppm (Figure 2 and S10). The doublet of triplet multiplicity (dt, ^2^ *J*_C,P_=30 Hz, ^2^ *J*_C,P_=17 Hz) confirms that all three phosphine moieties are bound to Ni. The assignment is additionally confirmed by the observation of three‐bond ^1^H‐^13^C coupling by HMBC analysis (Figure S13). Complex **2** was isolated in 61 % yield as a black solid.

**Scheme 1:**
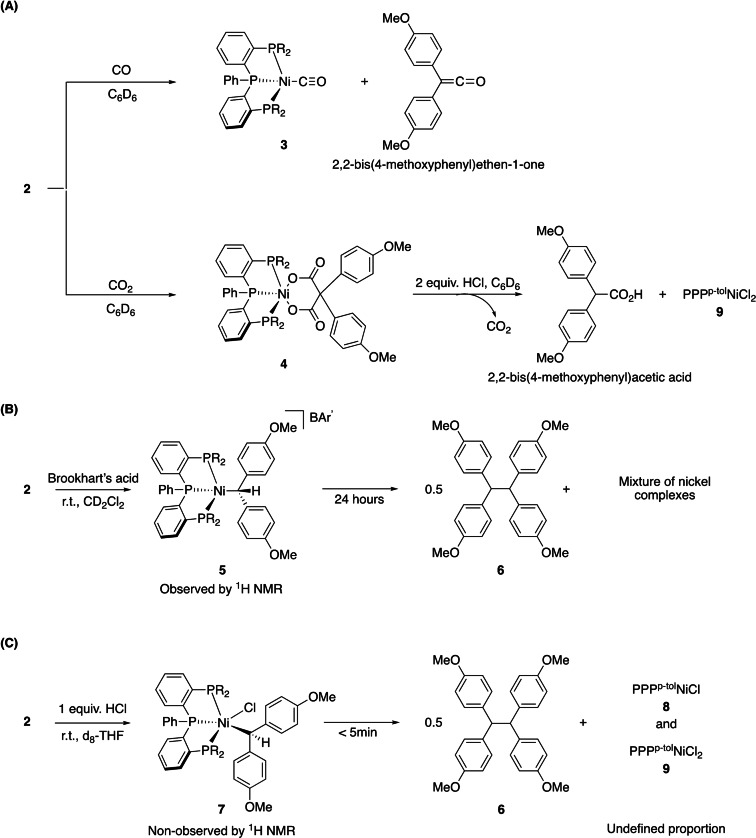
(A) Reactivity of complex 2 with CO and CO_2_. (B) Reaction of complex 2 with Brookhart's acid. (C) Reaction of complex **2** with HCl.

Complex **2** reacts rapidly with CO (1 atm) in C_6_D_6_, showing a solution color change from black to light yellow (Scheme 1, A). NMR and IR analysis show the total conversion of complex **2** to free 2,2‐bis(4‐methoxyphenyl)ethen‐1‐one and complex (PPP^*p*−tol^)Ni^0^(CO) (**3**, Figures S16 and S18), which could also be independently generated from complex **1** with CO (Figures S14 and S15). This result demonstrates the ability of **2** to transfer the carbene fragment to a small molecule in milder conditions than reported for (dtbpe)NiCPh_2_. The reaction of complex **2** with CO_2_ led to the formation of a metallalactone complex **4** via a possible [2+2] sequential cycloaddition of two CO_2_ equivalents (Figures S19‐S21). Strong IR ester signals at 1602 cm^−1^ and 1643 cm^−1^, similar to those reported by Hillhouse,^[8]^ testify the presence of two ester groups in the metallacycle. In addition, the reaction complex **4** with HCl in ether leads to the formation of 2,2‐bis(4‐methoxyphenyl)acetic acid, the expected product of protolysis of the dicarboxylate ligand in **4** (Figure S24).^[25]^

As nickel carbenes are generally nucleophilic,^[26]^ we also investigated the protonation of compound **2**. The reaction with Brookhart's acid [H(OEt_2_)_2_]^+^{[3,5‐(CF_3_)_2_C_6_H_3_]_4_B}^−^ in CD_2_Cl_2_ at room temperature (Scheme 1, B) was first tested. A ^1^H multiplet centered at 4.93 ppm, shown to couple to three ^31^P nuclei by ^1^H{^31^P} NMR analysis, suggests the formation of transient Ni^II^(alkyl) complex **5** in the reaction mixture. Over time, complex **5** degrades to form coupling product **6** (1,1,2,2‐tetrakis(4‐methoxyphenyl)ethane) and a complex mixture of Ni complexes (Figures S27 and S28). In contrast, the reaction of **2** with one equivalent of HCl (diethyl ether solution, Scheme 1, C) in d_8_‐THF at room temperature led to the rapid formation of a stoichiometric amount of **6** together with a mixture of paramagnetic complex (PPP^*p‐tol*^)Ni(Cl) (**8)** and diamagnetic complex (PPP^*p‐tol*^)NiCl_2_ (**9)**. EPR analysis confirmed the presence of complex **8**, and ^31^P{^1^H} NMR analysis confirmed the presence of complex **9** (Figures S31 and S32). To account for these observations, we propose the initial formation of a Ni^II^(alkyl) complex (either **5** or **7**), which undergoes homolytic cleavage of the nickel alkyl ligand bond (Scheme 1B and 1 C) to form a Ni^I^ center and a stabilized alkyl radical that ultimately dimerizes to form **6**. This contrasts with the reactivity of the dtbpe analogue, for which the Ni^II^(alkyl) complex resulting from protonation is stable and can be isolated.^[8]^

**Figure 3:**
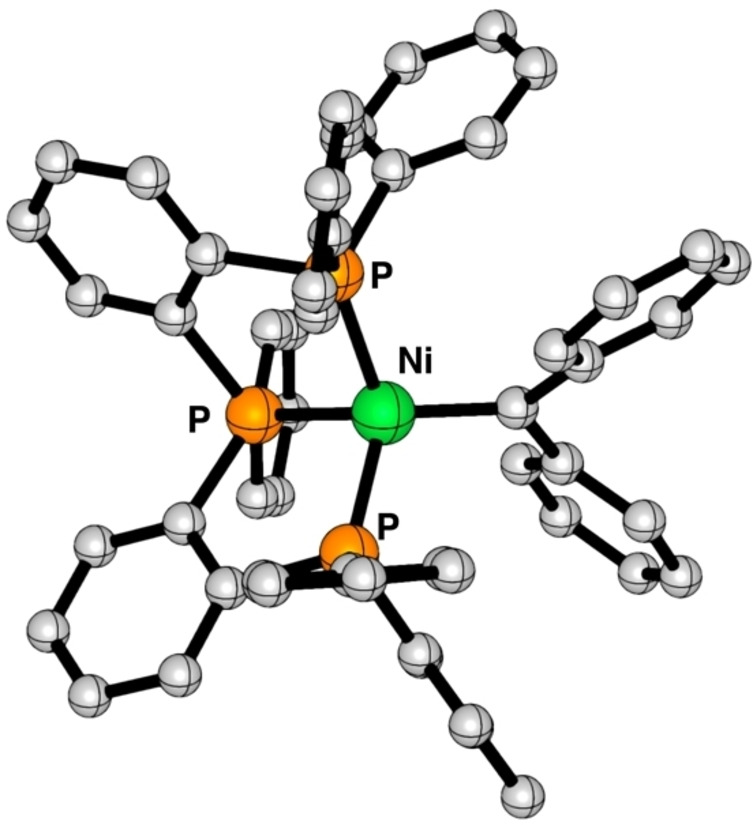
Optimized structure of **2** calculated at the M06L‐D3/6‐31g(d,p) level of theory. Hydrogen atoms are omitted for clarity.

DFT calculations[^16^, ^17^, ^18^] provide more insight into the nature of the nickel−carbene interaction in compound **2**. First, the geometry of a slightly truncated model of **2** bearing phenyl instead of *p*‐tolyl and *p*‐anisyl substituents is optimized at M06L‐D3/6‐31 g(d,p) (Figure 3). The nickel carbene **2** presents a distorted tetrahedral geometry where the calculated Ni−C bond length is somewhat longer (1.854 Å, Table 1) than the analogous bond of the optimized (dtbpe)NiCPh_2_ (1.805 Å, Figure S35) and the corresponding X‐ray crystal structure (1.836(2) Å).^[8]^ A conformational search reveals alternative geometries, involving the coordination of only two of the three phosphine moieties of the ligand, which are significantly less stable (Figure S36).

**Table 1:** Activation strain and energy decomposition analyses (in kcal mol^−1^) of the Ni−carbene complexes and Ni−carbene bond length (in Å).^[a,b]^

Complex	Δ*E*	Δ*E*_strain_	Δ*E*_int_	Δ*V*_elstat_	Δ*E*_Pauli_	Δ*E*_oi_	Δ*E*_disp_	*r*(Ni⋅⋅⋅C)
(dtbpe)NiCPh_2_	−86.2	12.4	−98.6	−169.1	207.5	−112.3	−24.7	1.805
(PPP^Ph^)NiCPh_2_	−74.1	9.8	−83.9	−176.0	223.9	−94.2	−37.6	1.854

[a] Computed at ZORA‐BLYP‐D3(BJ)/TZ2P//M06L‐D3/6‐31g(d,p). [b] See Table S1 for analyses at M06L‐D3/def2TZVP//M06L‐D3/6‐31g(d,p).

To get more insight into the chemical bonding of (PPP^Ph^)NiCPh_2_, Quantum Theory of Atoms in Molecules (QTAIM)^[27]^ and Energy Decomposition Analyses (EDA) within the framework of quantitative Kohn–Sham molecular orbital theory[^19^, ^20^, ^21^, ^22^] were performed. QTAIM is a topological analysis of the electron density that can provide information about the nature of the Ni−carbene bond as donor‐acceptor interaction (Fischer carbenes, prone to nucleophilic attack at carbon) or covalent delocalized bonds (Schrock carbenes, carbon nucleophiles).[^28^, ^29^] Analysis of the Laplacian map of the π‐plane at the carbene carbon (perpendicular to the CPh_2_ plane) of (PPP^Ph^)NiCPh_2_ shows an area of charge depletion in the direction of the p_π_ orbital of the carbon atom of the carbene. This indicates that the electrons of the carbene fragment are primarily located in the sp^2^‐hybridized orbital, engaging in a donor‐acceptor interaction with nickel, and the p_π_‐like orbital is depleted (Figure S37). Notably, the same feature is also observed for (dtpbe)NiCPh_2_ (Figure S38). In contrast, analysis of the Laplacian map of the π‐plane of Piers’ carbene pincer complex (PC_carbene_P)NiPPh_3_ shows a charge concentration around the carbon atom showing characteristics reminiscent to Schrock carbenes (Figure S39).[^11^, ^28^] These results suggest that, even though Ni carbenes exhibit nucleophilic reactivity (see above), their electronic structure can resemble that of a Fischer carbene. Similarly, Grubbs’ Ru−carbene catalysts have been shown to present characteristics of both Fischer and Schrock carbenes.^[30]^

The ASM[^19^, ^20^] is used to analyze and compare the bonding mechanism between the Ligand−Ni and the CPh_2_ fragments in the Ni−carbene complexes (dtpbe)NiCPh_2_ and (PPP^Ph^)NiCPh_2_ (Table 1).[^16^, ^28^, ^31^, ^32^] In this model, the electronic bond energy (Δ*E*) is decomposed intro the strain energy (Δ*E*_strain_), that is, the penalty that needs to be paid for deforming the fragment upon binding, and the interaction energy (Δ*E*_int_), which accounts for all mutual interaction that occur between the deformed fragment. We find that the Ni−carbene bond is for our newly synthesized (PPP^Ph^)NiCPh_2_ less stabilizing than for (dtpbe)NiCPh_2_, that is, the bond energy is −74.1 kcal mol^−1^ for (PPP^Ph^)NiCPh_2_ and −86.2 kcal mol^−1^ for (dtpbe)NiCPh_2_. Note that the same trend can be found in the bond dissociation enthalpies and Gibbs free energies of the Ni−carbene bonds (Table S1). This trend in bond energy is exclusively determined by the interaction energy, whereas the strain energy is only small and even follows the opposite trend.

To understand the origin of the trend in interaction energy in more detail, we decompose the interaction energy into four physically meaningful terms using the EDA: the classical electrostatic interaction (Δ*V*_elstat_), the steric (Pauli) repulsion (Δ*E*_Pauli_) arising from the repulsion between occupied closed‐shell orbitals of both fragments, the orbital interaction (Δ*E*_oi_) that accounts for donor‐acceptor interaction between the fragments and polarization within a fragment, and the dispersion energy (Δ*E*_disp_).[^21^, ^22^] This difference in interaction energy originates from (i) a more destabilizing steric (Pauli) repulsion; and (ii) less stabilizing orbital interactions for (PPP^Ph^)NiCPh_2_ compared to (dtpbe)NiCPh_2_. The former is a direct consequence of the increased steric repulsion between the phenyl groups of (PPP^Ph^)Ni and CPh_2_, which are, due to their spatial orientation, in closer proximity than the *tert*‐butyl groups of (dtpbe)Ni and CPh_2_. This, in fact, not only amplifies the steric (Pauli) repulsion but also forces the Ni−carbene bond length to be longer for (PPP^Ph^)NiCPh_2_ (1.854 Å) compared to (dtpbe)NiCPh_2_ (1.805 Å). Additionally, we have analyzed the Ni−carbene bond of (PPP^Ph^)NiC(*p*‐anisyl)_2_ (**2**) using the EDA which showed no significant energetic difference with (PPP^Ph^)NiCPh_2_ (Table S3).

**Figure 4:**
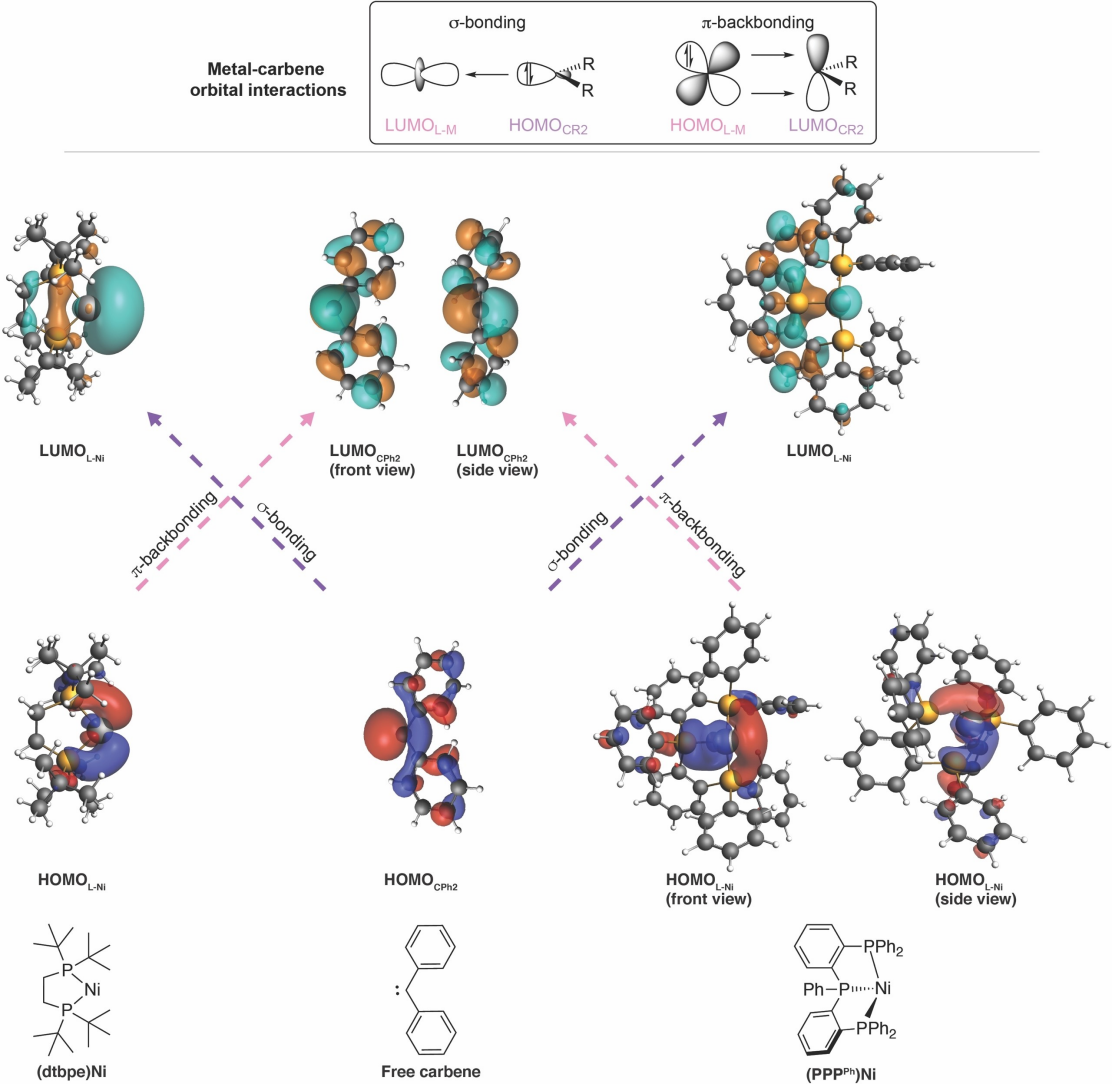
Molecular orbitals of the Ligand−Ni and carbene engaging in the σ‐bonding (HOMO_CPh2_‐LUMO_L−Ni_) and π‐backbonding (HOMO_L−Ni_‐LUMO_CPh2_) interaction, computed at ZORA‐BLYP‐D3(BJ)/TZ2P//M06L‐D3/6‐31g(d,p) level of theory.

**Figure 5:**
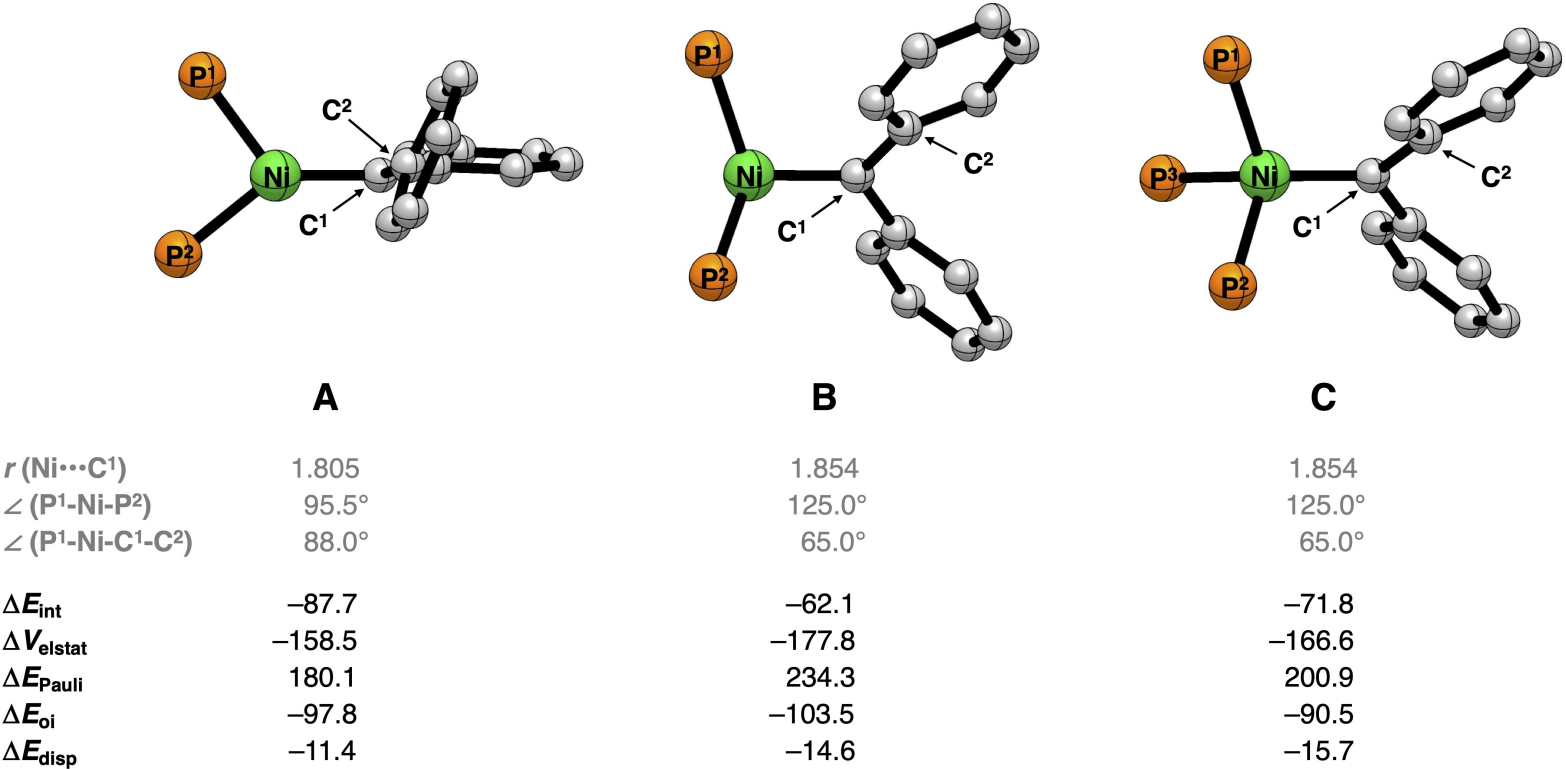
Energy decomposition analyses (in kcal mol^−1^) and geometical parameters (in Å and °) of the simplified (PH_3_)_2_NiCPh_2_ (**A**, **B**) and (PH_3_)_3_NiCPh_2_ (**C**) complexes computed at ZORA‐BLYP‐D3(BJ)/TZ2P//M06L‐D3/6‐31(d,p) level. The simplified Ni−carbene complexes were constructed by replacing the phenyl substituents by hydrogen atoms from the optimized structures of (dtbpe)NiCPh_2_ (complex **A**), (PPP^Ph^)NiCPh_2_ without the apical phosphine ligand (complex **B**), and (PPP^Ph^)NiCPh_2_ with the apical phosphine ligand (complex **C**). Hydrogen atoms are omitted for clarity.

The reduction of stabilizing orbital interactions from −112.3 kcal mol^−1^ for (dtpbe)NiCPh_2_ to −94.2 kcal mol^−1^ for (PPP^Ph^)NiCPh_2_ can be directly understood by analyzing the molecular orbitals responsible for the interaction between the Ligand−Ni and the CPh_2_ fragments in the Ni−carbene complexes, that is, (i) σ‐bonding between the occupied sp^2^‐hybridized orbital of CPh_2_ (HOMO_CPh2_) and the unoccupied a_1_‐like orbital of Ligand−Ni (LUMO_L−Ni_); (ii) π‐backbonding between the unoccupied p_π_‐like orbital of CPh_2_ (LUMO_CPh2_) and the occupied b_2_‐like orbital of Ligand−Ni (HOMO_L−Ni_) (Figure 4). As proposed by Hillhouse and coworkers,^[8]^ the orbitals of the L_2_Ni fragment are a perfect match for both σ‐bonding and π‐backbonding interactions with the CPh_2_ fragment (Figure 1). The perpendicular conformation of the carbene fragment in (dtpbe)NiCPh_2_ stand to maximize the π‐backbonding interaction. In the case of (PPP^Ph^)NiCPh_2_, however, both the HOMO and LUMO, engaging in the σ‐bonding and π‐backbonding interactions with the CPh_2_, are delocalized over the pincer ligand, which supresses stabilizing orbital interactions. Kohn–Sham molecular orbital analysis quantifies this and demonstrates that both the σ‐bonding and π‐backbonding orbital interaction weaken from (PPP^Ph^)NiCPh_2_ to (dtpbe)NiCPh_2_, due to a loss of build‐up in stabilizing orbital overlap as a result of both a smaller HOMO_L−Ni_ and LUMO_L−Ni_ lobe at the external face of (PPP^Ph^)Ni and a longer Ni−carbene bond length (Figure S41). This further explains the facile reactivity of (PPP^Ph^)NiCPh_2_ with small molecules like CO. Next, we aim to further study the influence of the phosphine ligand in apical position of (PPP^Ph^)NiCPh_2_ on the Ni−carbene bond. To obtain a more detailed insight, we perform an EDA on simplified Ni−carbene complexes (Figure 5, see SI), where the phenyl groups bound to the phosphine ligands are replaced by hydrogen atoms and systematically go, in two steps, from the simplified (dtpbe)NiCPh_2_ (complex **A**) to the simplified (PPP^Ph^)NiCPh_2_ without the apical phosphine ligand (complex **B**) to the simplified (PPP^Ph^)NiCPh_2_ with the apical phosphine ligand (complex **C**). Our analyses reveal that the apical phosphine ligand polarized the occupied orbital density away from the external face, thereby reducing the steric (Pauli) repulsion between the simplified (PPP^Ph^)Ni and CPh_2_ fragments and increasing the Ni−carbene bond strength. By going from complex **A** to complex **B**, that is, by increasing the Ni−carbene bond length and P^1^−Ni−P^2^ bond angle to that of (PPP^Ph^)NiCPh_2_, significantly weakens the Ni−carbene interaction from −87.7 kcal mol^−1^ for **A** to −62.1 kcal mol^−^ for **B**. This weakening is a direct result of the increased steric repulsion between the phosphine ligands and CPh_2_, which are in closer proximity, as also reflected by the increase in steric (Pauli) repulsion. Introducing the apical phosphine ligand, hence having a complex that resembles (PPP^Ph^)NiCPh_2_, leads to a remarkable stabilization of the interaction energy from −62.1 kcal mol^−1^ for **B** to −71.8 kcal mol^−1^ for **C**. This stabilization can be traced back to the π‐accepting character of the apical phosphine ligand, which, upon binding, pulls occupied orbital density away from the incoming CPh_2_ fragment, thereby lowering the destabilizing steric (Pauli) repulsion between the simplified (PPP^Ph^)Ni and CPh_2_ fragments (Figure S45). This effect compensates the observed weakening of the stabilizing electrostatic and orbital interactions, corresponding to going from complex **B** to complex **C**.

## Conclusions

In conclusion, we report the synthesis and characterization of a stable tetracoordinated nickel carbene (PPP^p−Tol^)NiC(*p*‐anisyl)_2_. The complex is reactive towards small molecules such as CO and CO_2_, and its nucleophilic character is evidenced by its reactivity towards acids. Computational methods support the tetracoordinated geometry of (PPP^Ph^)NiCPh_2_ as the most stable structure, with the Ni−C bond best viewed as a donor‐acceptor interaction. Computational studies using the ASM and EDA provides a view of the interaction between (PPP^Ph^)Ni and CPh_2_ in this complex. This analysis shows that the key σ‐bonding and π‐backbonding interactions are possible for (PPP^Ph^)NiCPh_2_ but somewhat weaker than for its 3‐coordinate analogues. Interestingly, EDA also reveals that the third, apical phosphine ligand provides stabilization to the tetracoordinated nickel carbene by polarizing occupied orbital density away from its external face and thereby reducing the steric (Pauli) repulsion between (PPP^Ph^)Ni and CPh_2_. Overall, these results show that electron‐rich metal carbenes can be stabilized even with relatively high coordination numbers, opening new possibilities for developing the chemistry of these reactive intermediates.

## Conflict of Interests

The authors declare no conflict of interest.

1

## Supporting information

As a service to our authors and readers, this journal provides supporting information supplied by the authors. Such materials are peer reviewed and may be re‐organized for online delivery, but are not copy‐edited or typeset. Technical support issues arising from supporting information (other than missing files) should be addressed to the authors.

Supporting Information

## Data Availability

The data that support the findings of this study are available in the supplementary material of this article.
