# Supplementary material for: Synthesis, Reactivity, and Bonding Analysis of a Tetracoordinated Nickel Carbene
Source: Chemistry. 2024 Oct 23;30(68):e202403211. doi: 10.1002/chem.202403211 (PMC11618040; doi:10.1002/chem.202403211)
Supplement: Supplementary file 1 — Supporting Information [file CHEM-30-e202403211-s001.pdf]

Chemistry–A European Journal

Supporting Information

Synthesis, Reactivity, and Bonding Analysis of a Tetracoordinated Nickel Carbene

Pablo M. Pérez-García, María L. G. Sansores-Paredes, Célia Fonseca Guerra, Pascal Vermeeren,* and Marc-Etienne Moret*

Supporting information for:

Synthesis, Reactivity, and Bonding Analysis of a Tetracoordinated Nickel Carbene

Pablo M. Pérez-García,[1,2‡] María L. G. Sansores-Paredes,[1‡] Célia Fonseca Guerra,[3] Pascal Vermeeren,[3*] and Marc-Etienne Moret[1*]

[1] Organic Chemistry and Catalysis, Institute for Sustainable and Circular Chemistry, Faculty of Science, Utrecht University, Universiteitsweg 99, 3584 CG, Utrecht, The Netherlands.

[2] Universidad Privada Boliviana (UPB), Cochabamba, Bolivia

[3] Department of Chemistry and Pharmaceutical Sciences, AIMMS, Vrije Universiteit Amsterdam, De Boelelaan 1108, 1081 HZ Amsterdam, The Netherlands.

TABLE OF CONTENTS

EXPERIMENTAL SECTION: *2*

Chemicals and Reagents **2**

Physical Methods **2**

Synthesis of the active Ni^0 complex from the reduction of $(PPP^{p\text{-}tol})Ni^I(Br)$ with sodium naphthalenide (complex 1) **2**

Synthesis of the carbene $(PPP^{p\text{-}tol})Ni{=}C(p\text{-}C_6H_4OMe)_2$ (complex 2): **5**

Reaction of complex 1 with CO to generate $(PPP^{p\text{-}tol})Ni(CO)$ (complex 3): **9**

Reaction of complex 2 with CO: **11**

Reaction of complex 2 with CO_2 to form $(PPP^{p\text{-}tol})Ni[(O_2C)_2C(p\text{-}C_6H_4OMe)_2]$ (complex 4): **13**

Protonation of complex 4: **15**

Synthesis of complex 9 $PPP^{p\text{-}tol}NiCl_2$ **18**

Reaction of complex 2 with $[H(Et_2O)]^+[[3,5\text{-}(CF_3)_2C_6H_3]_4B]^-$: **19**

Reaction of complex 2 with HCl dissolved in ether: **22**

Synthesis of complex $(PPP^{p\text{-}tol})Ni^ICl$ (8) for the comparison of the EPR spectrum: **24**

COMPUTATIONAL SECTION *24*

General information **24**

DFT calculations **25**

QTAIM comparison of reported Ni carbenes **27**

Additional notes of the EDA analysis **30**

Cartesian coordinates of optimized structures **35**

REFERENCES: *62*

EXPERIMENTAL SECTION:

Chemicals and Reagents

Unless otherwise noted, all reactions were carried out under an inert $N_{2(g)}$ atmosphere, using standard Schlenk line or glovebox techniques, and stirred magnetically. Deuterated solvents were purchased from Cambridge Isotope Laboratory Incorporation (Cambridge, USA) and were degassed by standard freeze−thaw−pump procedure[1] and subsequently stored over molecular sieves. Common solvents were purified using a MBRAUN MB SPS-80 purification system or by standard distillation techniques or both. They were degassed by bubbling $N_{2(g)}$ through the liquid for at least 30 min and then stored over molecular sieves. Non-halogenated solvents were tested with a standard purple solution of sodium benzophenone ketyl in tetrahydrofuran to confirm effective oxygen and moisture removal. Other solvents were checked for water content by the Karl Fischer titration. Liquid chemicals were first degassed by standard freeze−pump−thaw procedures or purged with $N_{2(g)}$ and then stored over molecular sieves prior to use.[1] ($PPP^{p\text{-tol}}$)Ni^{I}(Br),[2] Bis(4-methoxyphenyl)diazomethane,[3] and Brookhart's acid $[H(Et_2O)]^+\{[3,5\text{-}(CF_3)_2C_6H_3]_4B\}^{-4}$ were prepared following literature procedures. Phosphorus containing compounds were checked for oxidation by ^{31}P NMR before use. All other reagents and starting materials were purchased from commercial sources and used without further purification, except when specified. Additionally, 4,4'-(diazomethylene)bis(methoxybenzene) and HBArF were stored in the glovebox after synthesis at -40°C.

Physical Methods

The ^1H, ^{13}C, and ^{31}P(400, 100, and 161 respectively) spectra were recorded at 297 K on an Agilent MRF 400 spectrometer. All chemical shifts are reported in the standard δ notation of parts per million, referenced to residual peak of the solvent, as determined relative to Me_4Si (δ = 0 ppm) and ^{31}P NMR chemical shifts were externally referenced to 85% aqueous H_3PO_4 solution. EPR analyses were carried on a Bruker EMX Plus 6000 Gauss machine with ER 041 XG X-Band Microwave Bridge. Infrared spectra were recorded using a Perking Elmer Spectrum One FT-IR spectrometer under N_2 flow.

Synthesis of the active Ni^0 complex from the reduction of ($PPP^{p\text{-tol}}$)Ni^{I}(Br) with sodium naphthalenide (complex 1)

Under N_2 atmosphere, napththalene (6.0 mg, 0.047 mmol) was dissolved in 1mL of THF at room temperature. A lump of sodium was added and the resulting dark green mixture was stirred for 3 h at room temperature. ($PPP^{p\text{-tol}}$)Ni^{I}(Br) (40 mg, 0.047 mmol) was dissolved in 2 mL of THF in a separate vial and cooled to -78 °C. The sodium naphthalide mixture was filtered and added dropwise over 5 min to the suspension of solution of ($PPP^{p\text{-tol}}$)Ni^{I}(Br). The resulting deep red solution was stirred at –78 °C for 1.25 h and then overnight at room temperature. The solution was filtered and dried in vacuo to evaporate the solvent and remove the residual naphtalene resulting in a 32 mg of a red powder (88 % yield).
Spectroscopic analysis in solution (^1H NMR, ^{31}P NMR, DOSY, IR, see figures S1-5) show that the Ni^0 complex is a mixture of a dimer with a N_2 bridging ligand and a monomer with a N_2 end-on coordinated ligand. Similar systems reported in the literature support our hypothesis.[5.6]

$^{31}P\{^1H\}$ NMR (161 MHz, C_6D_6): δ 47.16 (t, J = 65.8 Hz, 1P), 39.14 (d, J = 66.0 Hz, 2P).
$^{31}P\{^1H\}$NMR (161 MHz, C_6D_6): δ 40.26 (t, J = 67.8 Hz, 1P), 35.47 (d, J = 74.7 Hz, 2P).

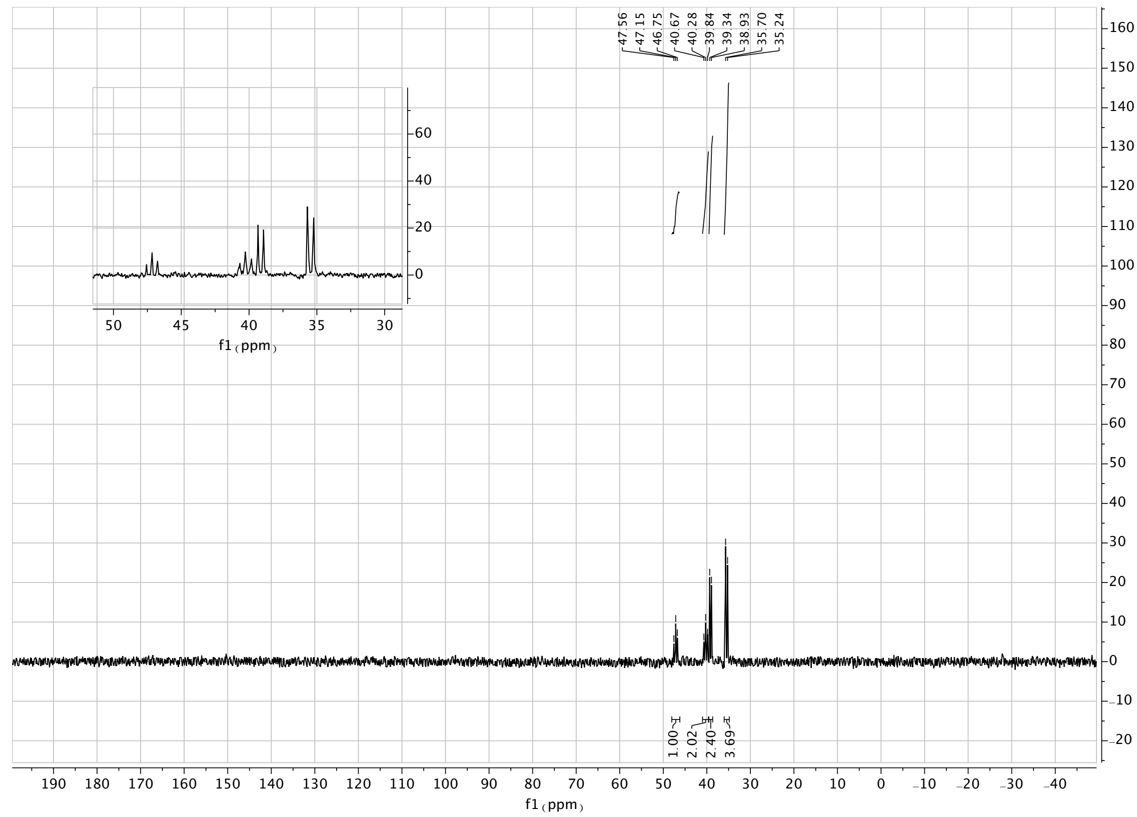

Figure S1: $^{31}P\{^{1}H\}$ NMR spectrum of Ni^{0} complexes synthesized from the reduction of $(PPP^{p\text{-tol}})Ni^{I}(Br)$ in C_6D_6 at room temperature.

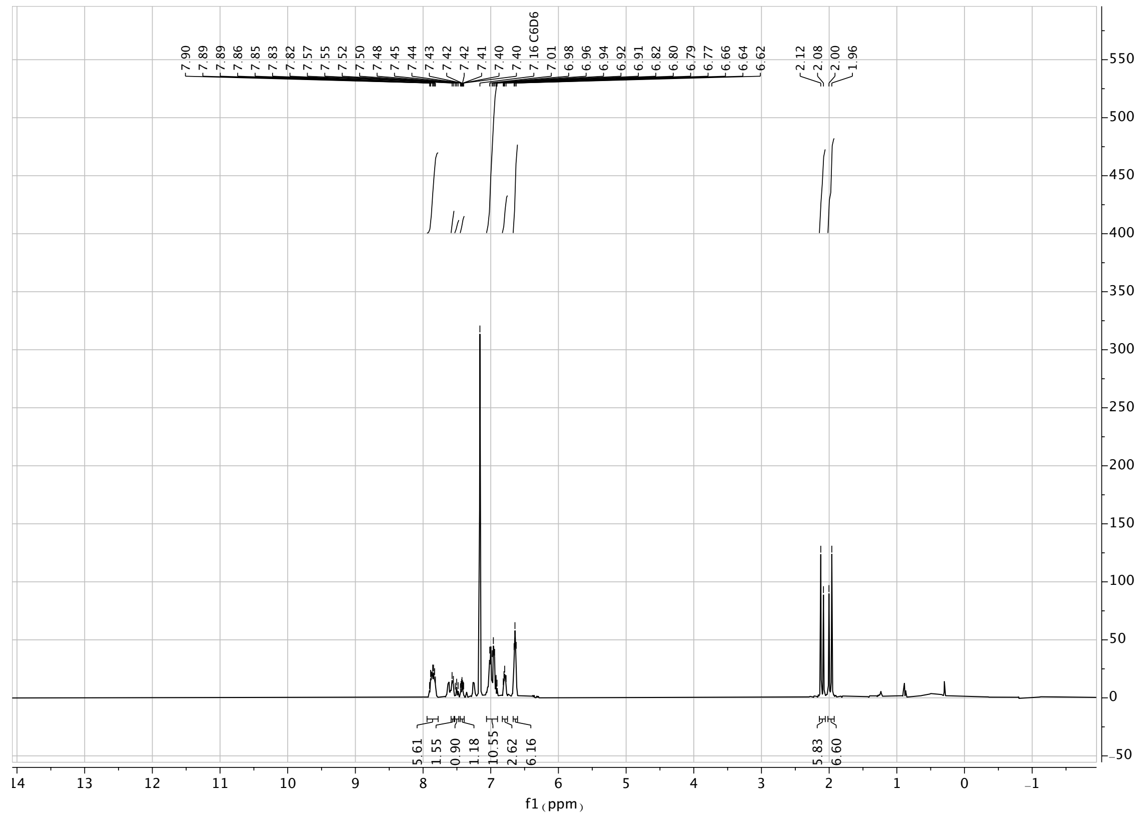

Figure S2: ^{1}H NMR spectrum of Ni^{0} complexes synthesized from the reduction of $(PPP^{p\text{-tol}})Ni^{I}(Br)$ in C_6D_6 at room temperature.

The IR and the DOSY analysis show that the Ni^0 complex is a mixture of a dimer with a N_2 bridging ligand and a monomer with a N_2 end-on coordinated ligand. Similar systems reported in the literature support our hypothesis.[5,6]

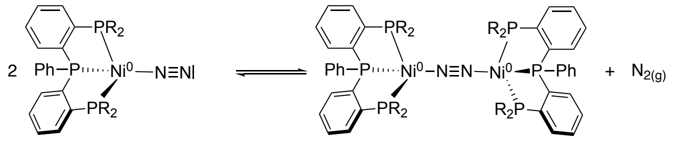

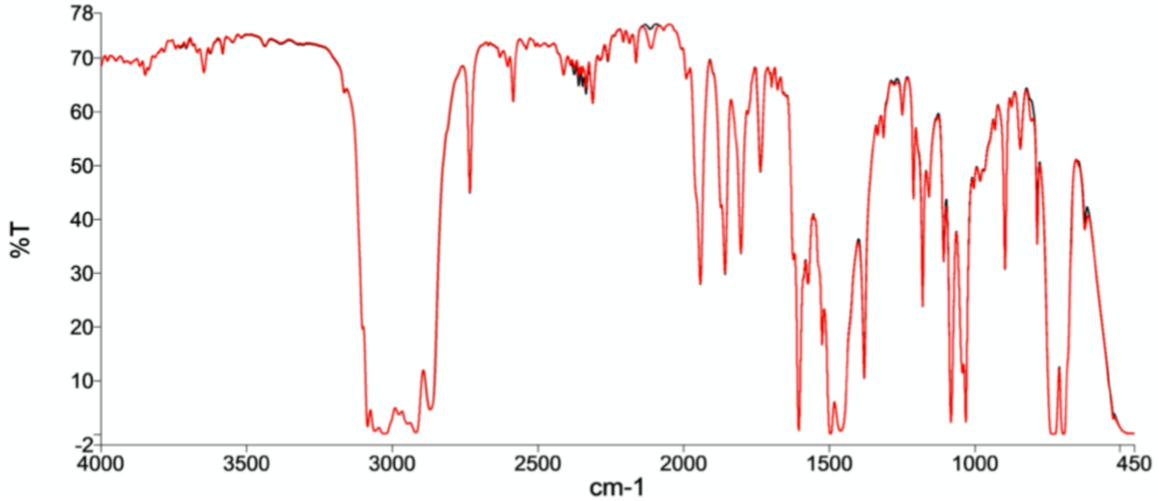

Figure S3: Solution IR spectra of complex **1** broad range (red spectrum). Black spectrum corresponds to pure THF solvent.

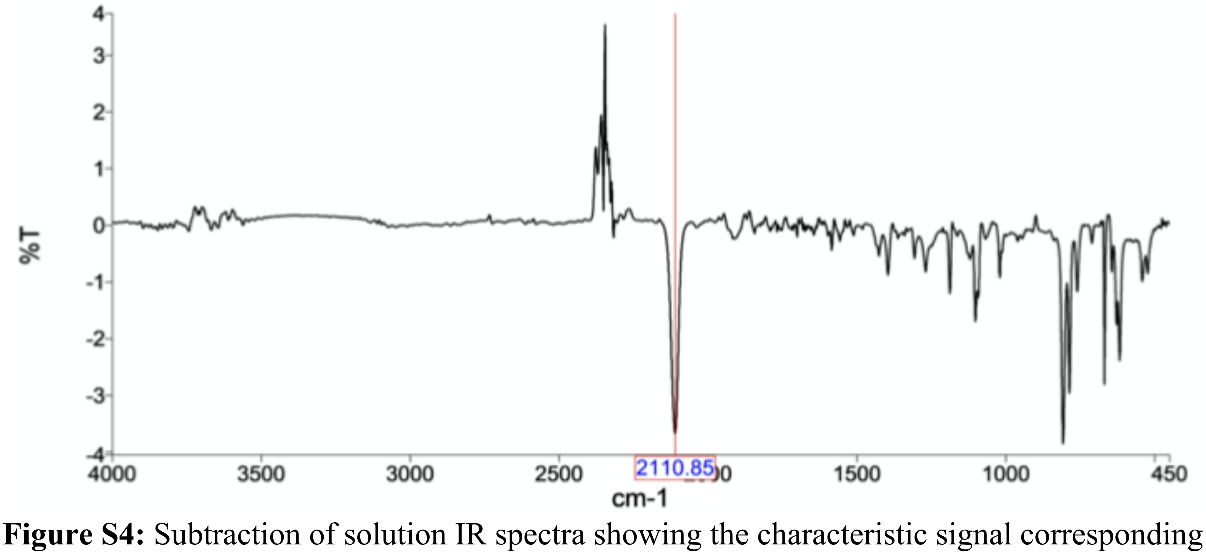

Figure S4: Subtraction of solution IR spectra showing the characteristic signal corresponding to an end-on coordination nitrogen.

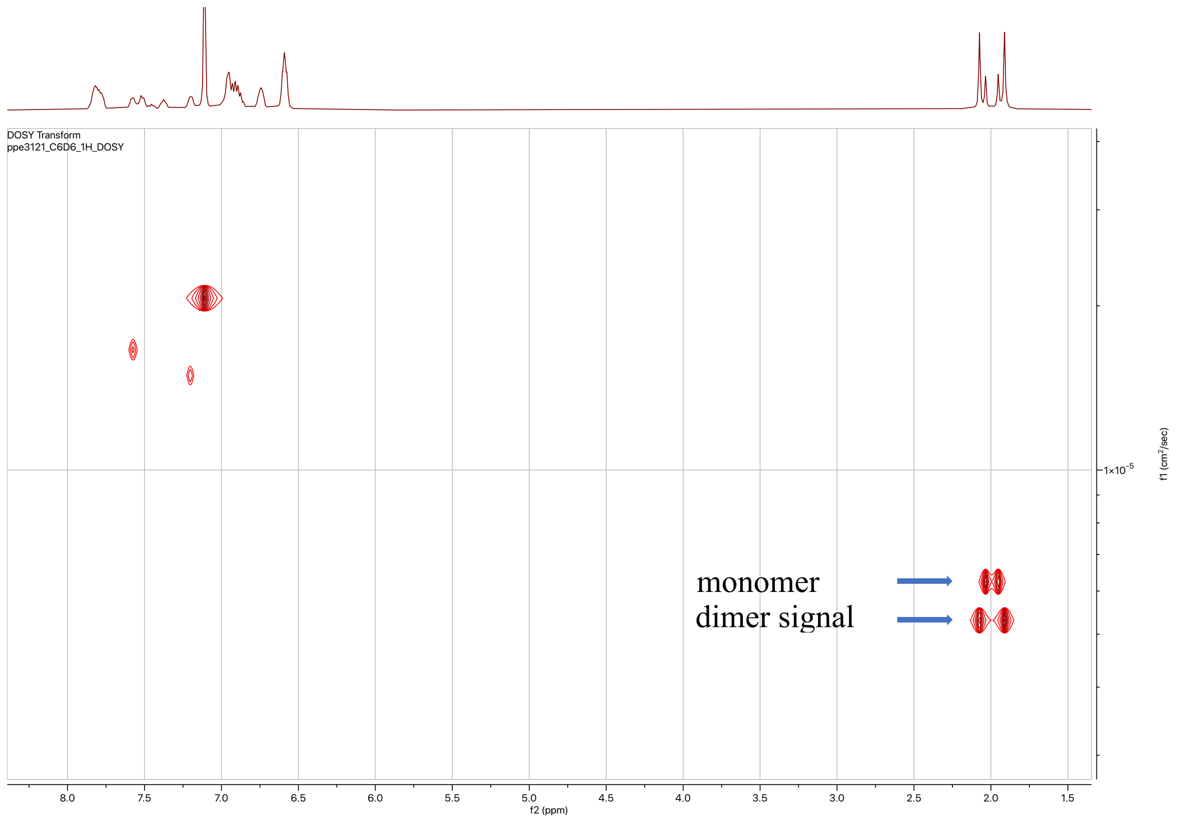

Figure S5: DOSY spectrum of the (PPP$^{p\text{-tol}}$)Ni^0 complexes mixture in C_6D_6 at room temperature.

Synthesis of the carbene (PPP$^{p\text{-tol}}$)Ni=C(*p*-C_6H_4OMe)$_2$ (complex 2):

Under a $N_{2(g)}$ atmosphere, 90 mg of complex **1** (0.12 mmol) were dissolved in 5 ml THF in a 20 mL vial. To this solution, 60 mg of Bis(4-methoxyphenyl)diazomethane (0.24 mmol, 2 equiv.) dissolved in 5 mL of THF were added dropwise at room temperature. The reaction was monitored by ^{31}P NMR analysis and showed completion after 5 days under magnetic stirring. The color of the solution during this reaction time evolved from red to black. The solution was filtered through glass fiber filter paper, and the solvent evaporated under vacuum. The residue was dissolved in hexane, precipitating a yellow solid (pressumably an organic homocoupling product dervided from the diazo precursor). The solid was removed by filtration, and ca. 90 % of the solvent was evaporated from the filtrate under vacuum. The concentrated solution was cooled down to –78°C and 5 mL of tetramethylsilane (TMS) were added, resulting in the formation of a black precipitate after several minutes. 70 mg of complex **2** were then recovered by filtration at –78°C and drying in vacuum as a black solid (61 % yield).

^{1}H NMR (400 MHz, Benzene-*d*$_6$) δ 7.93 (t, *J* = 5.8 Hz, 2H), 7.73 (d, *J* = 8.2 Hz, 4H), 7.55 (d, *J* = 7.4 Hz, 2H), 7.48 (d, *J* = 8.5 Hz, 4H), 7.40 (t, *J* = 8.4 Hz, 2H), 7.09 (d, *J* = 7.2 Hz, 3H), 7.07 – 6.98 (m, 7H), 6.94 (t, *J* = 7.5 Hz, 2H), 6.69 (t, *J* = 7.2 Hz, 7H), 6.47 (d, *J* = 8.3 Hz, 4H), 3.32 – 3.27 (m, 6H), 2.13 (s, 6H), 1.94 (s, 6H).
^{31}P{^{1}H} NMR (162 MHz, Benzene-*d*$_6$) δ 57.89 (t, *J* = 48.0 Hz, 1P), 47.64 (d, *J* = 48.0 Hz, 2P).

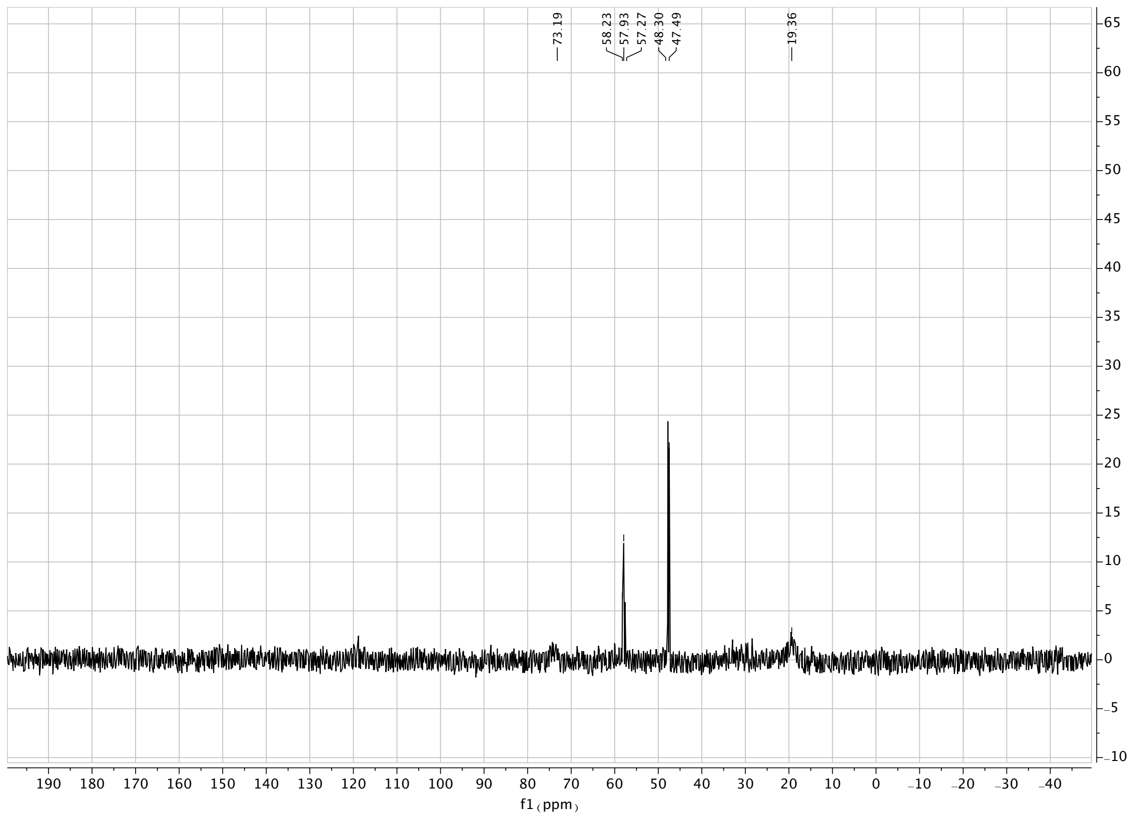

Figure S6: $^{31}P\{^1H\}$ NMR spectrum of the mixture of carbene **2** and presumed Ni(0) diazo compound measured in THF at room temperature. The reaction time was 16 hours.

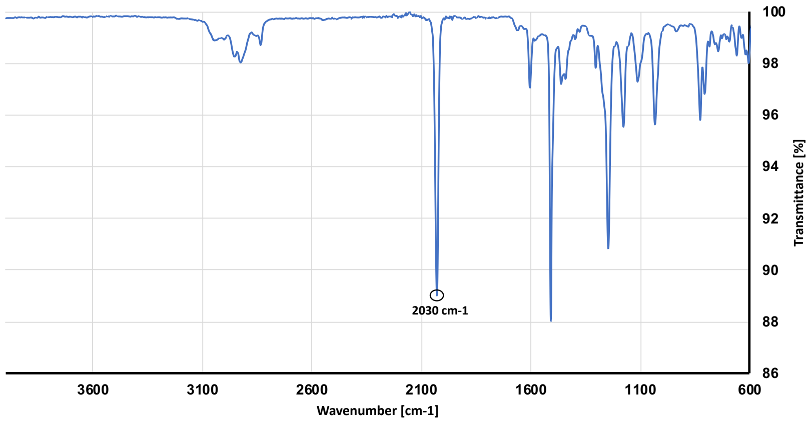

Figure S7: ATR-IR spectrum of the reaction mixture at 16 hours, the strong signal at 2030 cm^{-1} indicates the presence of the diazo intermediate.

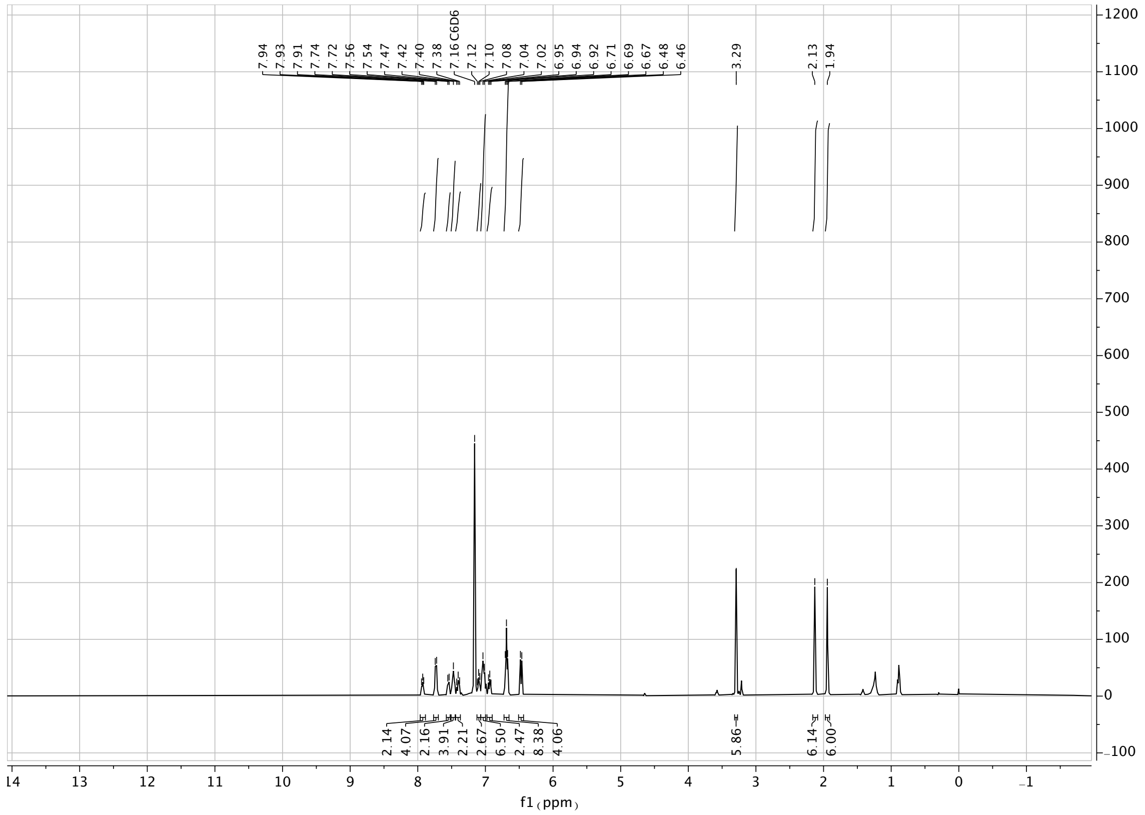

Figure S8: ^{1}H NMR spectrum of complex **2** in C_6D_6 at room temperature.

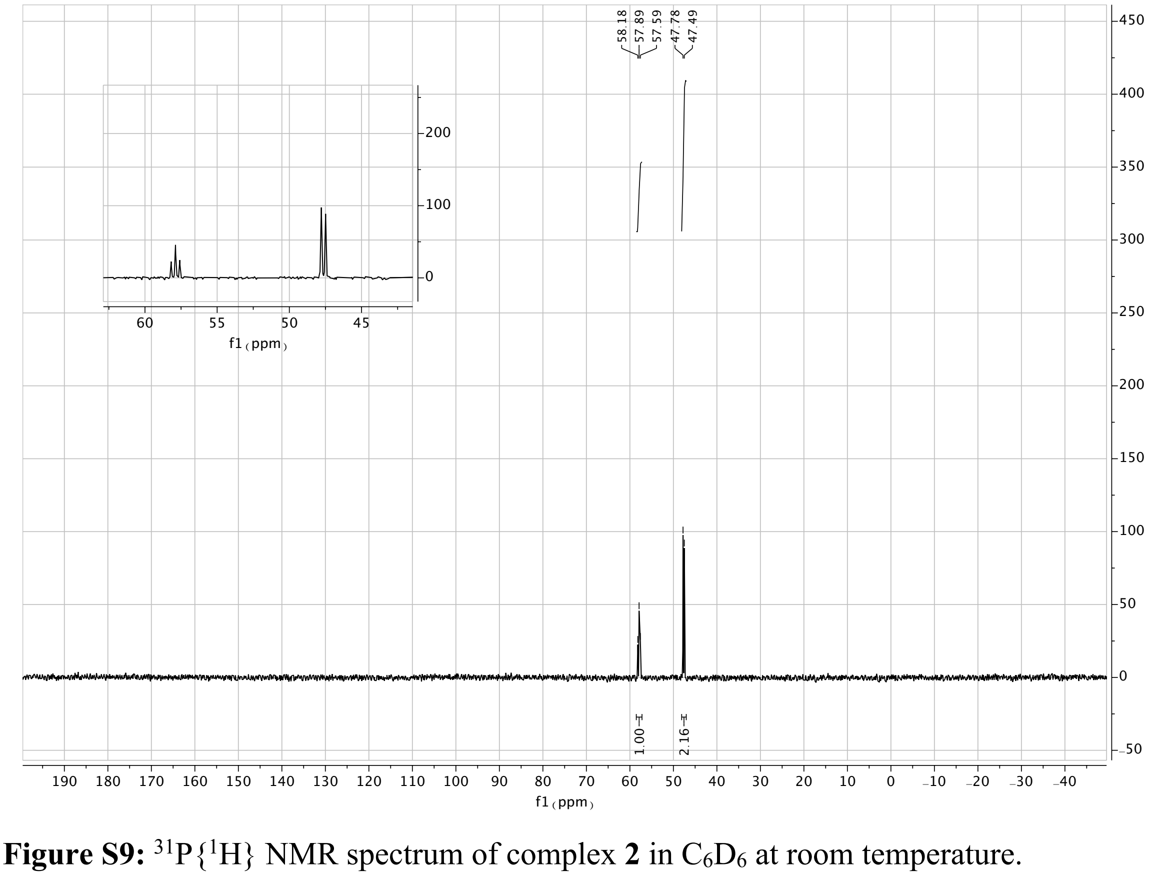

Figure S9: $^{31}P\{^{1}H\}$ NMR spectrum of complex **2** in C_6D_6 at room temperature.

The following ^{13}C NMR was recorded after 3 days of a saturated solution in C_6D_6, the signal corresponding to the carbene carbon appears at 288.85 (dt, J = 30, 17 Hz).

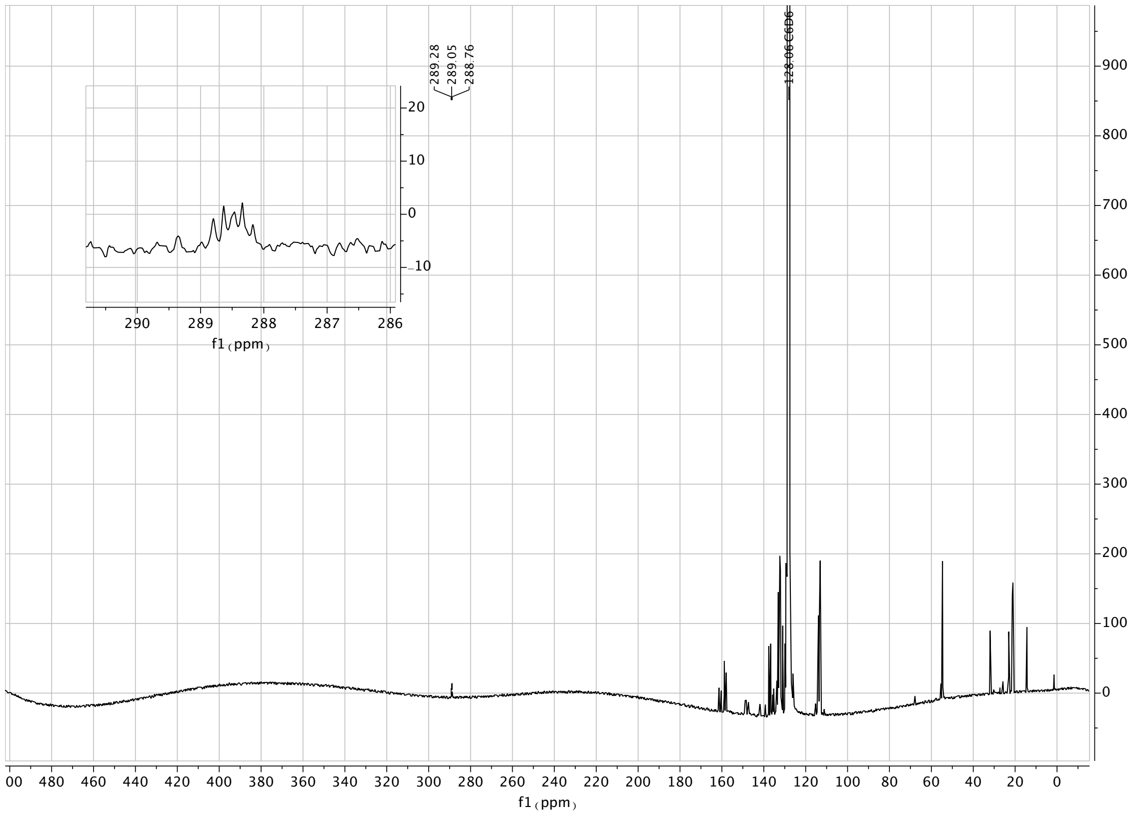

Figure S10: ^{13}C NMR spectrum of complex **2** in C_6D_6 at room temperature.

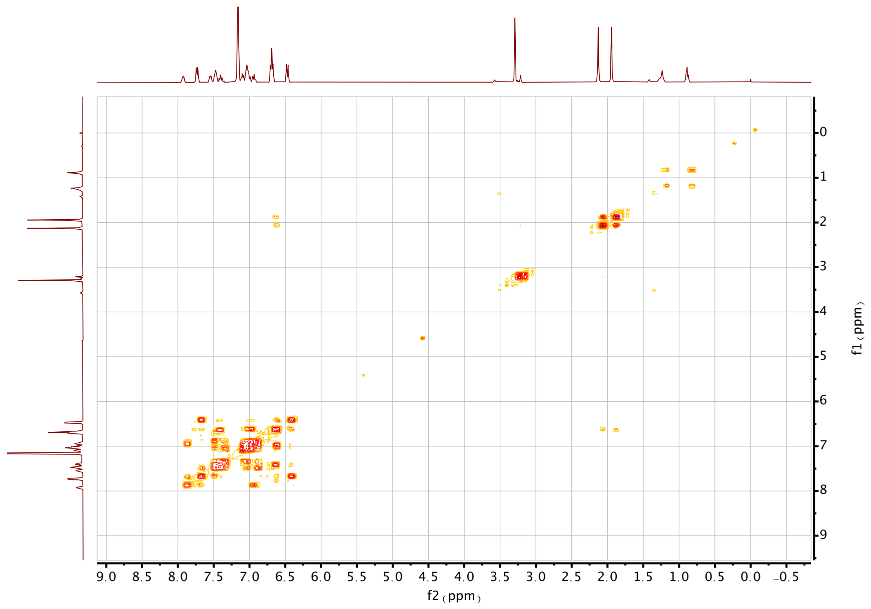

Figure S11: ^{1}H COSY spectrum of complex **2** in C_6D_6 at room temperature.

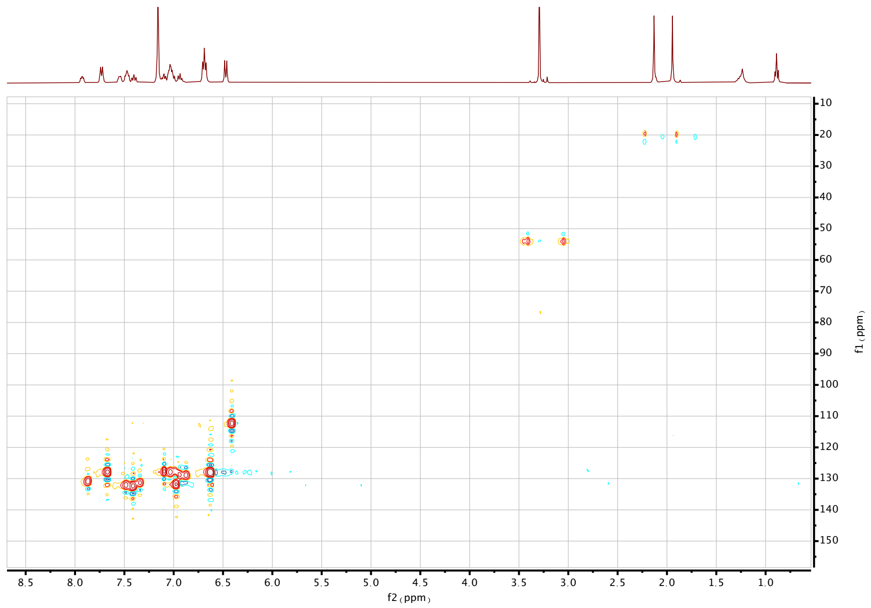

Figure S12: HSQC spectrum of complex **2** in C_6D_6 at room temperature.

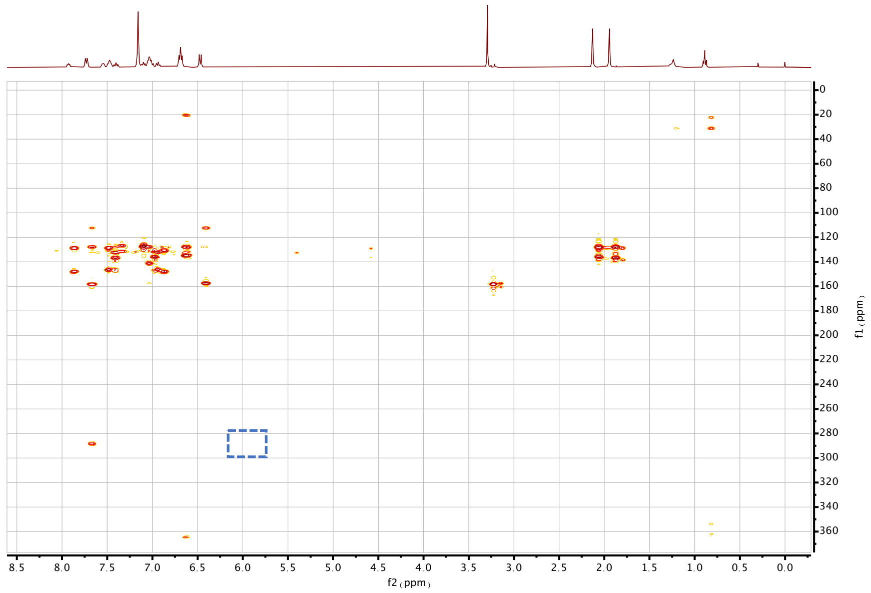

Figure S13: HMBC spectrum of complex **2** in C_6D_6 at room temperature. The carbene signal is highlighted by the blue dashed rectangle.

Reaction of complex 1 with CO to generate (PPP$^{p\text{-}tol}$)Ni(CO) (complex 3):

5 mg of complex **1** (0.006 mmol) were dissolved in 0.8 mL of C_6D_6 in a 20 mL vial. The resulting solution was transferred to a J-Young NMR tube. After 3 freeze-pump-thaw degassing cycles, the solution was exposed to 1 atm of CO. The color of the solution changed from red to yellow in less than 5 minutes. The sample was then submitted to different NMR and ATR-IR analysis and used as reference for the next experiment.

^{1}H NMR (400 MHz, Benzene-*d*$_6$) δ 8.05 – 7.96 (m, 4H), 7.85 – 7.77 (m, 2H), 7.60 – 7.56 (m, 2H), 7.52 – 7.45 (m, 2H), 7.05 – 6.99 (m, 4H), 6.98 – 6.95 (m, 2H), 6.94 – 6.90 (m, 5H), 6.88 – 6.82 (m, 4H), 6.56 (d, *J* = 7.7 Hz, 4H), 2.07 (s, 6H), 1.97 (s, 6H).

$^{31}P\{^{1}H\}$NMR (162 MHz, Benzene-d_6) δ 60.88 (t, J = 41.3 Hz, 1P), 47.32 (d, J = 41.6 Hz, 2P).

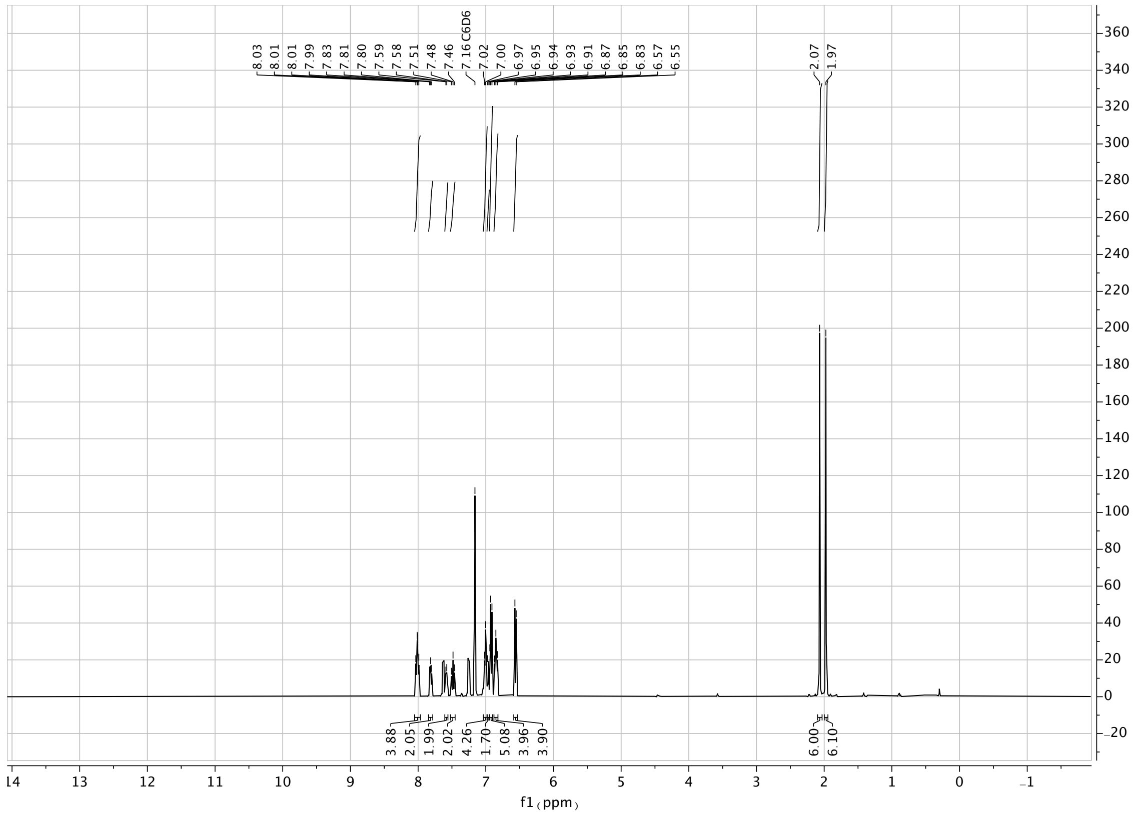

Figure S14: ^{1}H NMR spectrum of complex **3** in C_6D_6 at room temperature.

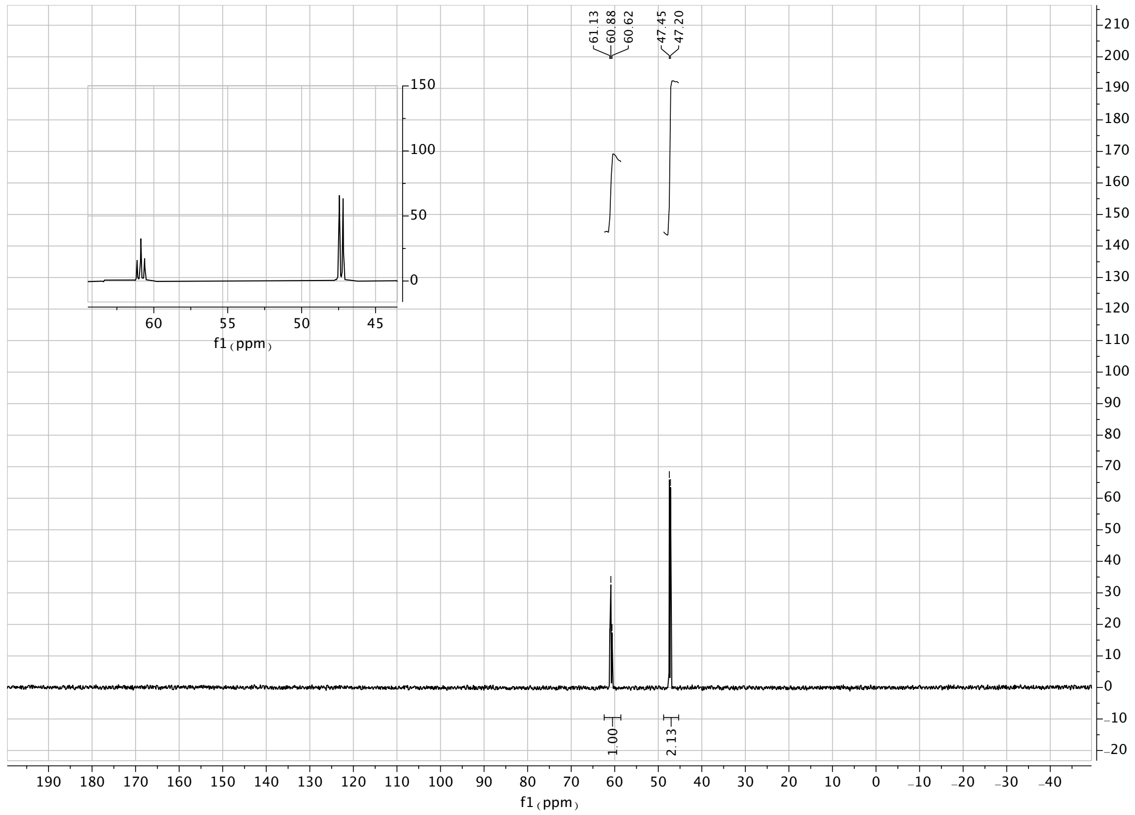

Figure S15: $^{31}P\{^1H\}$ NMR spectrum of complex **3** in C_6D_6 at room temperature.

Reaction of complex 2 with CO:

5 mg of complex **2** (0.005 mmol) were dissolved in 0.8 mL of C_6D_6 in a 20 mL vial. The resulting solution was transferred to a J-Young NMR tube. After 3 freeze-pump-thaw degassing cycles, the solution was exposed to 1 atm of CO. The color of the solution changed from black to yellow in less than 5 minutes. The sample was the submitted to different NMR and IR analysis showing the formation of complex **3** and 2,2-bis(4-methoxyphenyl)ethen-1-one.

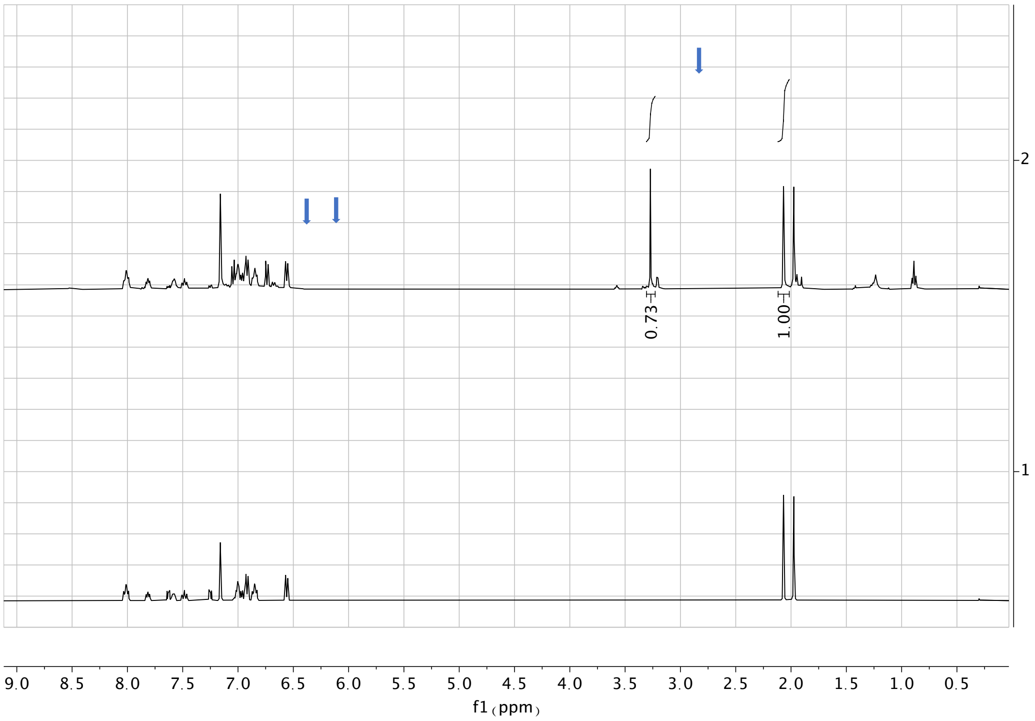

Figure S16: ^{1}H NMR spectra comparison of complex **3** (bottom) with the reaction between complex **2** and CO (top) in C_6D_6 at room temperature. The signals corresponding to 2,2-bis(4-methoxyphenyl)ethen-1-one are indicated with blue arrows.

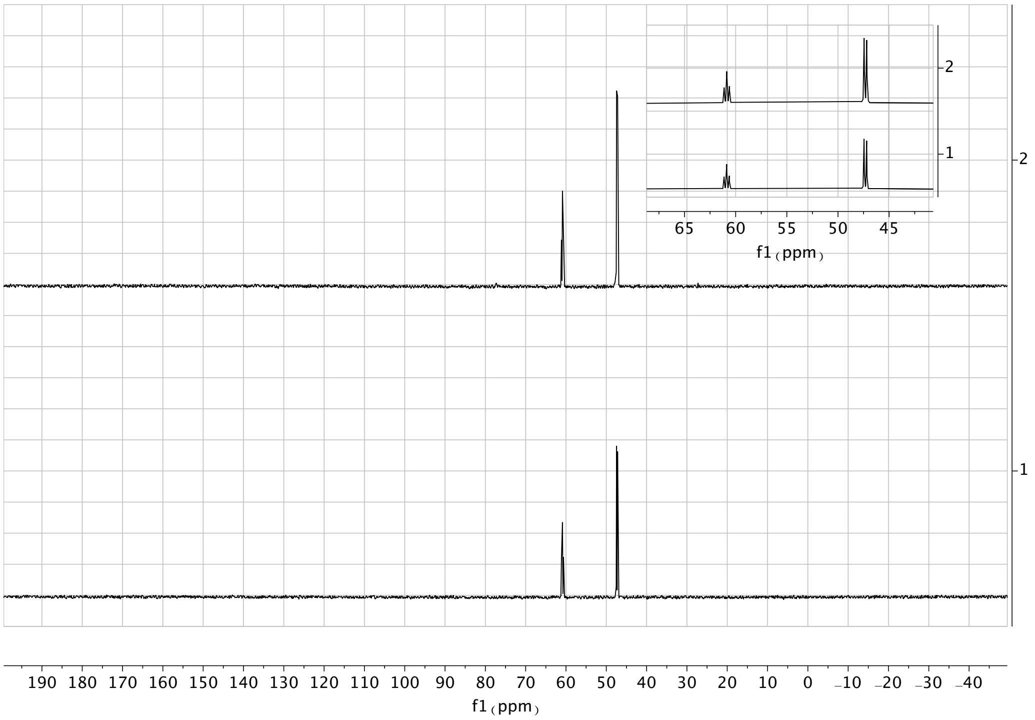

Figure S17: $^{31}P\{^{1}H\}$ NMR spectra comparison of complex **3** (bottom) with the reaction between complex **2** and CO (top).

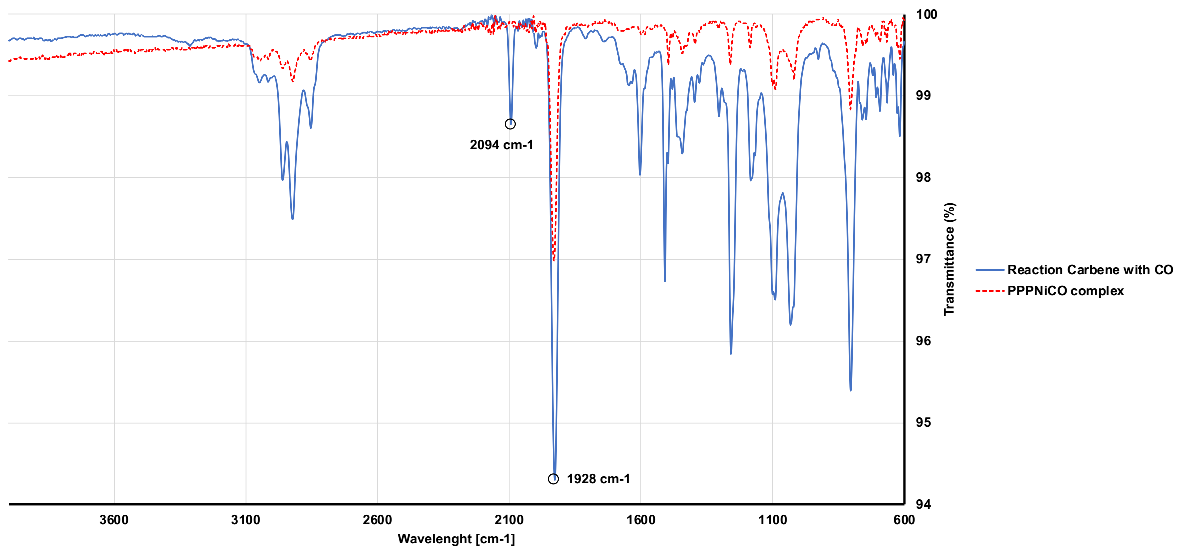

Figure S18: ATR-IR spectra comparison of complex **3** (red dashed) with the reaction between complex **2** and CO (blue line). The signal at 2094 cm^{-1} corresponds to 2,2-bis(4-methoxyphenyl)ethen-1-one.[7]

Reaction of complex 2 with CO_2 to form ($PPP^{p\text{-}tol}$)Ni[$(O_2C)_2C$(*p*-$C_6H_4OMe)_2$] (complex 4):

5 mg of complex **2** (0.005 mmol) were dissolved in 0.8 mL of C_6D_6 in a 20 mL vial. The resulting solution was transferred to a J-Young NMR tube. After 3 freeze-pump-thaw degassing cycles, the solution was exposed to 1 atm of CO_2 for 16 hours at room temperature. The color of the solution changed from black to light orange. The sample was the submitted to different NMR and IR analysis showing the formation of complex **4**. The solvent of the solution was then removed under vacuum and complex **4** was recovered as an orange powder (4 mg, 75 %).

^{1}H NMR (400 MHz, Benzene-*d*$_6$) δ 7.69 – 7.57 (m, 8H), 7.11 – 7.03 (m, 4H), 6.79 (dd, *J* = 10.2, 6.2 Hz, 13H), 6.73 (d, *J* = 7.5 Hz, 4H), 6.35 (d, *J* = 8.3 Hz, 4H), 3.25 (s, 6H), 2.11 (s, 6H), 1.89 (s, 6H).

^{31}P{^{1}H}NMR (162 MHz, Benzene-*d*$_6$) δ 43 (s, 1P), 14 (br, 2P).

IR (cm^{-1}): 3050, 2922, 2953, 2852, 1643, 1602, 1505, 1244, 1080.

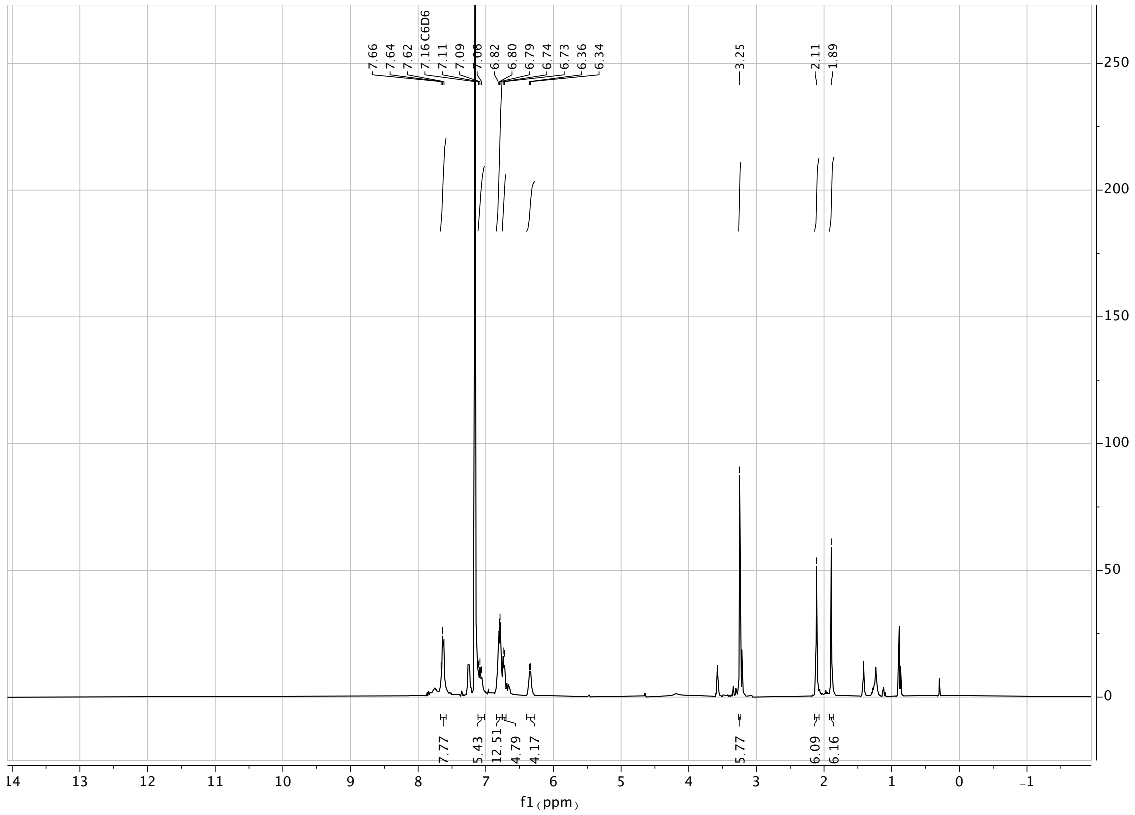

Figure S19: ^{1}H NMR spectrum for the reaction between complex **2** and CO_2 in C_6D_6 at room temperature.

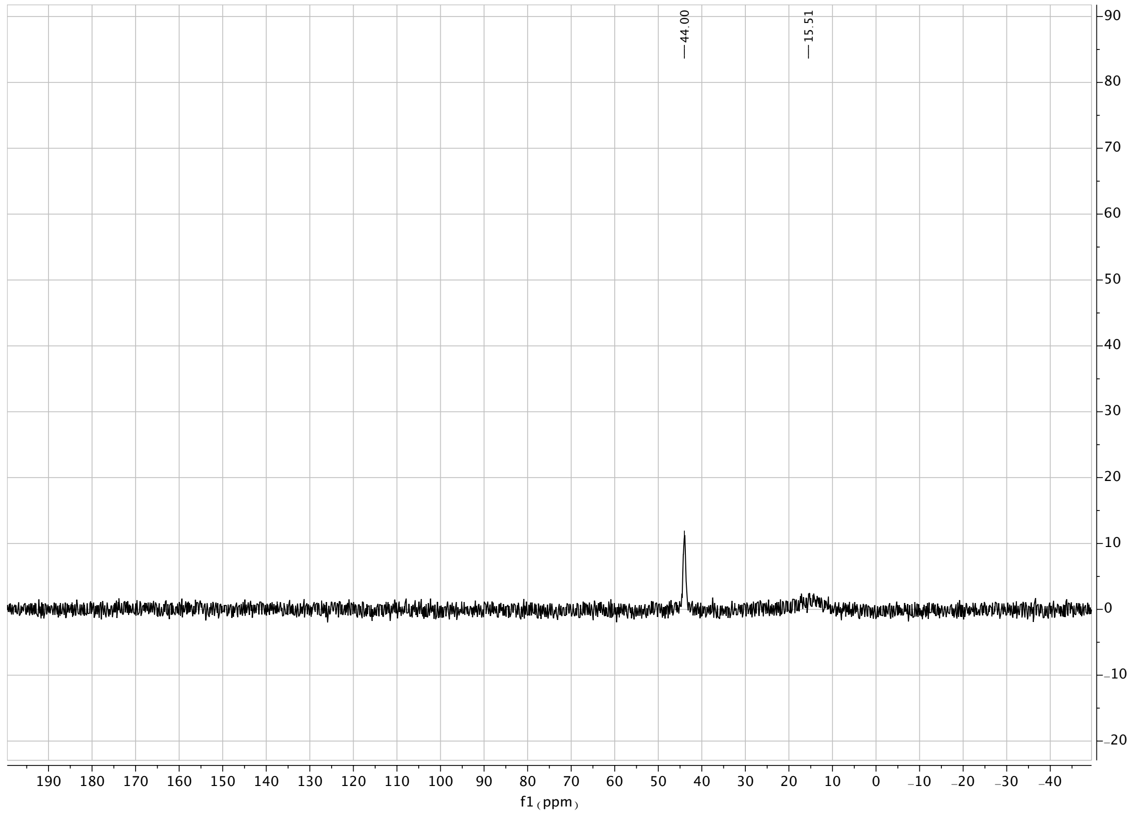

Figure S20: $^{31}P\{^{1}H\}$ NMR spectrum for the reaction between complex **2** and CO_2 in C_6D_6 at room temperature.

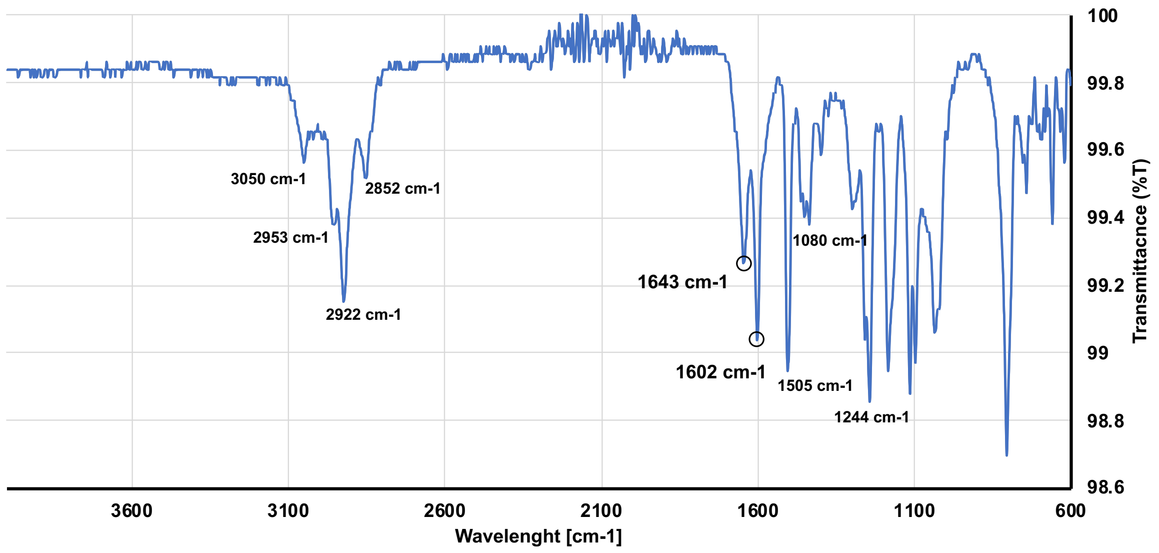

Figure S21: IR spectra for the reaction between complex **2** and CO_2. The signals corresponding to the ester groups (1602 and 1643 cm^{-1}) are similar to those previously reported for an analogous fragment (1618 and 1650 cm^{-1}).[8]

Protonation of complex 4:

5 mg of complex **2** (0.005 mmol) were dissolved in 0.8 mL of C_6D_6 in a 20 mL vial. The resulting solution was transferred to a J-Young NMR tube. After 3 freeze-pump-thaw degassing cycles, the solution was exposed to 1 atm of CO_2 for 16 hours. The color of the solution changed from black to light orange. The formation of complex **4** was controlled by ^{1}H and ^{31}P NMR analysis. Then, 10 µL of a 1 M HCl solution in ether were added under $N_{2(g)}$ atmosphere and the reaction was controlled by ^{1}H and ^{31}P NMR analysis. To remove residual diethyl ether, the solvents were evaporated, and the residue was redissolved in 0.8 mL of C_6D_6.

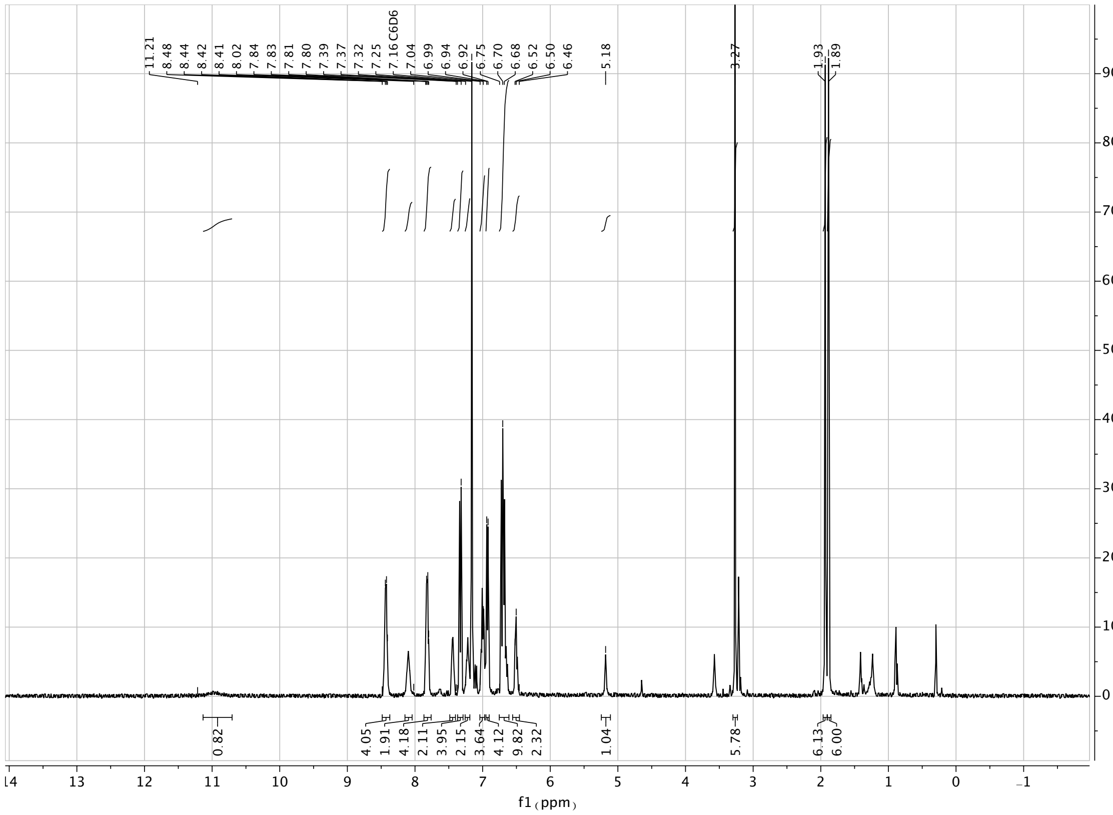

Figure S22: ^{1}H NMR spectrum from the protonation reaction of complex **4** in C_6D_6 at room temperature.

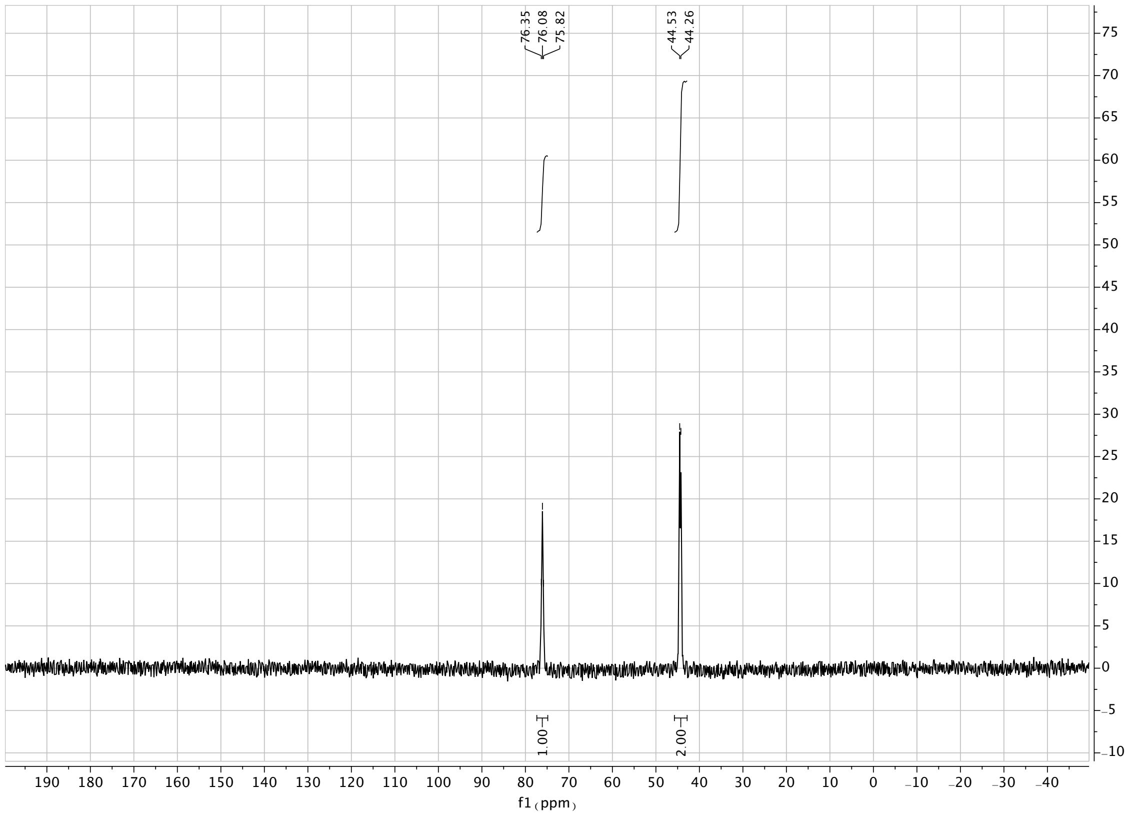

Figure S23: $^{31}P\{^{1}H\}$ NMR spectrum from the protonation reaction of complex **4** in C_6D_6 at room temperature.

The above presented spectra suggest that the protonation of complex **4** leads to the formation of 2,2-bis(4-methoxyphenyl)malonic acid and complex PPP$^{p\text{-tol}}$NiCl$_2$ (see independent synthesis below). The proton singlet at 5.18 ppm and the broad signal at 11 ppm (Figure S17) would indicate that the malonic acid undergoes monodecarboxylation to form 2,2-bis(4-methoxyphenyl)acetic acid. To support this hypothesis, the reaction solution was added to 1 mL of distilled water and extracted with 3 mL of diethyl ether. The organic phase was recovered, and the solvent evaporated under vacuum. The ^{1}H NMR analysis of the residue in CDCl$_3$ indeed corresponded to the compound 2,2-bis(4-methoxyphenyl)acetic acid.[9]

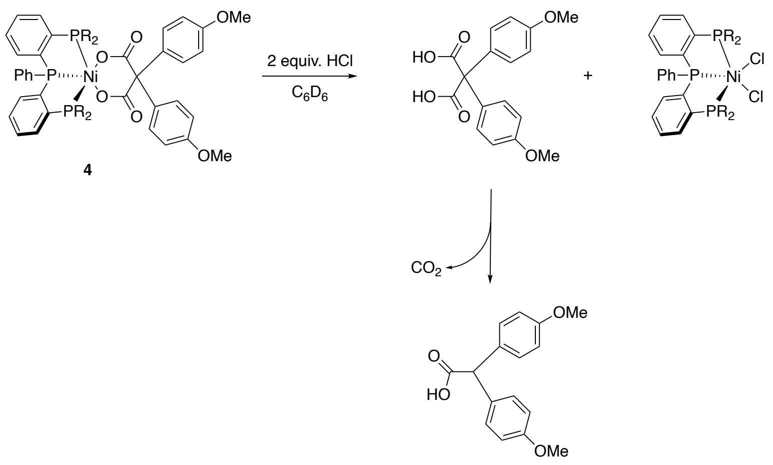

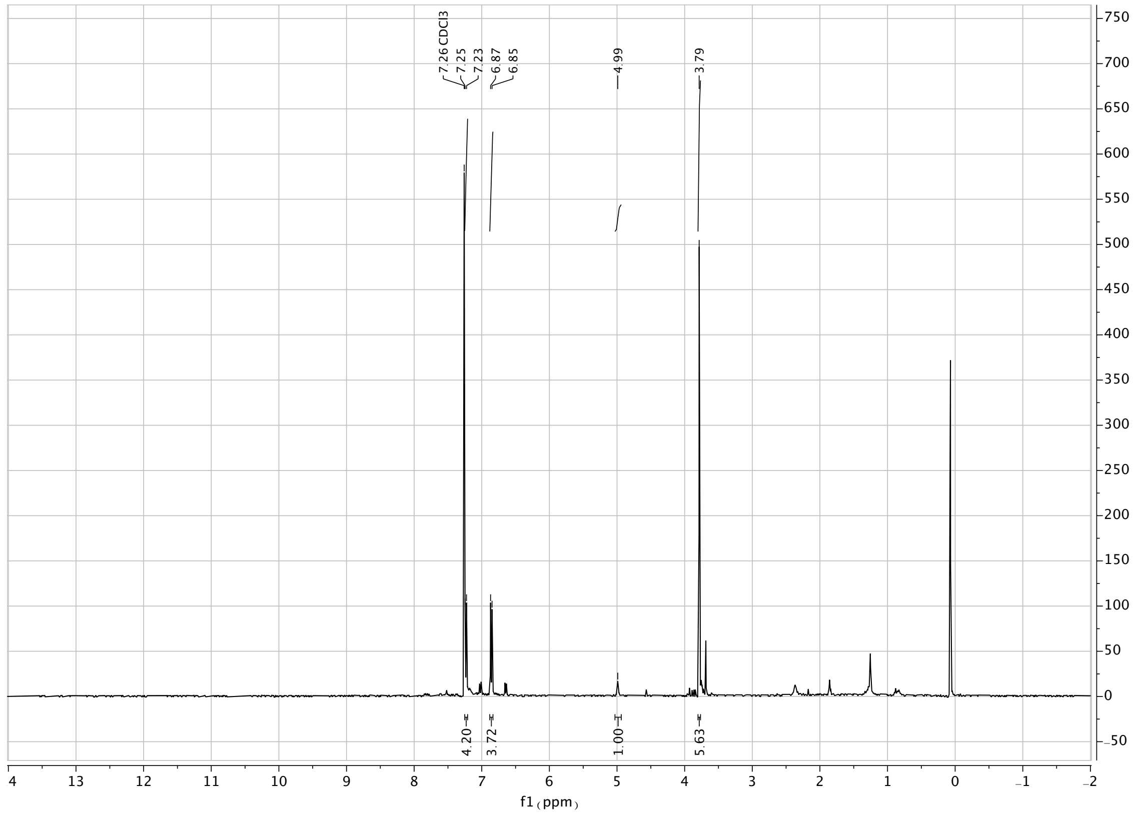

Figure S24: ^{1}H NMR spectra of the recovered organic part from the protonation reaction of complex **4** in CDCl$_3$ at room temperature.

Synthesis of complex 9 $PPP^{p\text{-}tol}NiCl_2$

In order to support our hypothesis of the formation of complex $PPP^{p\text{-}tol}NiCl_2$, this compound was independently synthesized with the following procedure. Under N_2 atmosphere, ligand $PPP^{p\text{-}tol}$ (30 mg, 0.044 mmol) was dissolved in 3mL of THF at room temperature. In a separate 20 mL vial, $NiCl_2$(dme) was dissolved in 2 mL of THF (10 mg, 0.044 mmol). The solution of ligand was added drop by drop to the nickel precursor solution and stirred at room temperature for 16 hours resulting in a green solution. The resulting solution was filtered and most of the solvent was evaporated, after which hexane was added resulting in the precipitation of a green solid. The solid was recovered by filtration, washed with hexane and then dried under vacuum. 31 mg of the complex were recovered resulting in a yield of 86%.

^{1}H NMR (400 MHz, Benzene-d_6) δ 8.52 (br, 4H), 7.91 (br, 5H), 7.53 (br, 2H), 7.01 (d, J = 7.5 Hz, 4H), 6.93 (d, J = 8.0 Hz, 6H), 6.70 (d, J = 7.8 Hz, 5H), 6.65 (d, J = 8.4 Hz, 1H), 6.50 (d, J = 7.9 Hz, 2H), 1.94 (s, 6H), 1.89 (s, 6H).
^{31}P{^{1}H} NMR (162 MHz, Benzene-d_6) δ 75.68 (br, 1P), 44.30 (br, 2P).

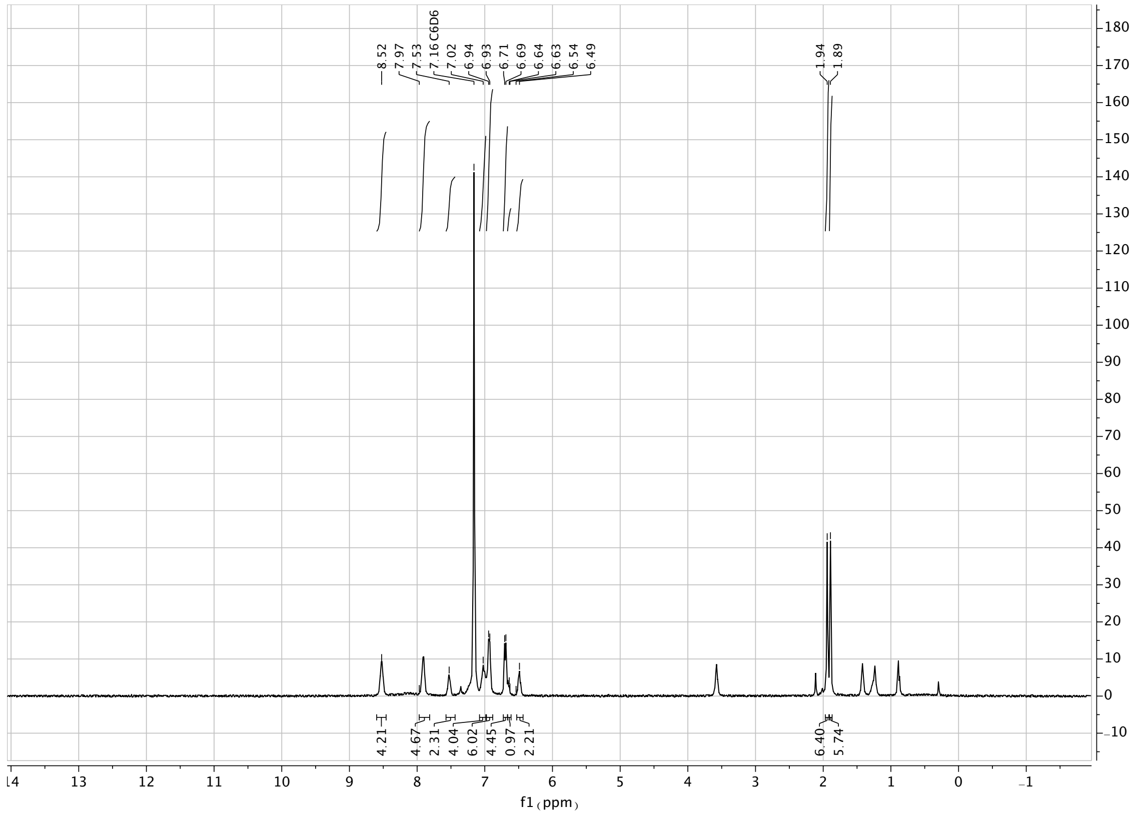

Figure S25: ^{1}H NMR of complex **9** $PPP^{p\text{-}tol}NiCl_2$ in C_6D_6.

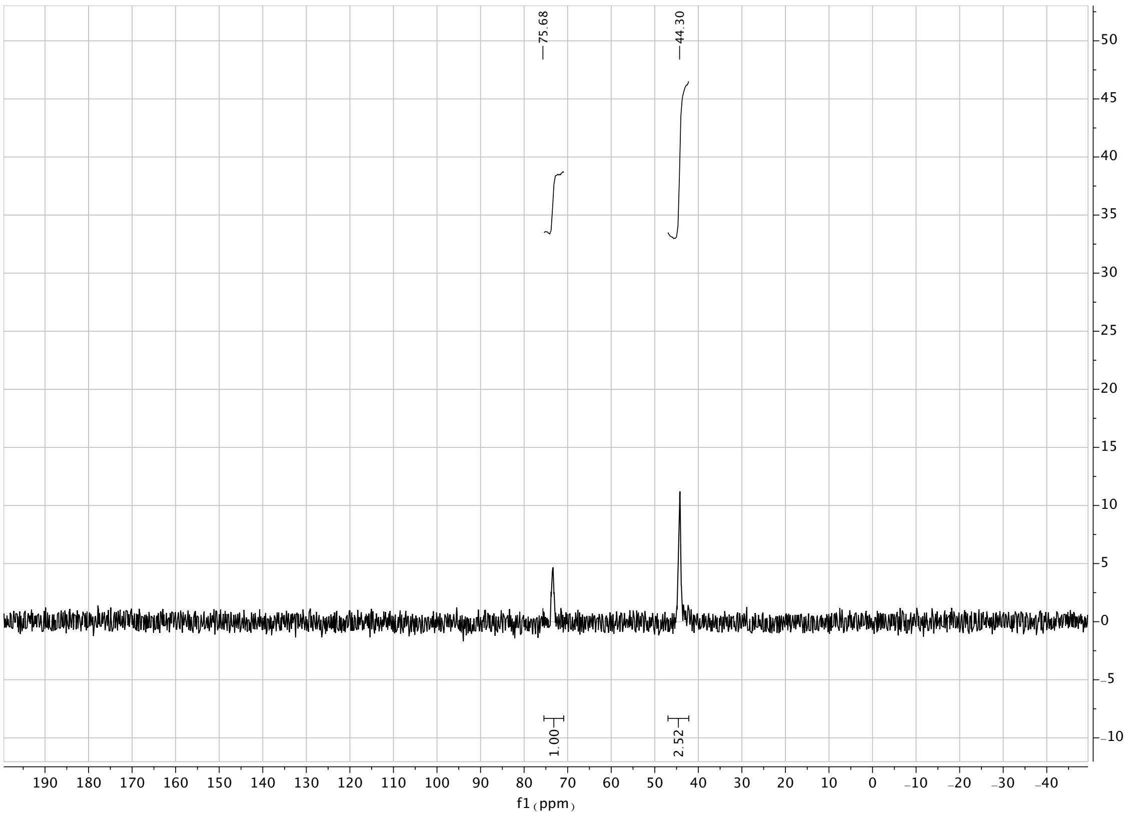

Figure S26: $^{31}P\{^{1}H\}$ NMR of complex **9** PPP$^{p\text{-}tol}$NiCl$_2$ in C_6D_6.

Reaction of complex 2 with $[H(Et_2O)]^+[[3,5\text{-}(CF_3)_2C_6H_3]_4B]^-$:

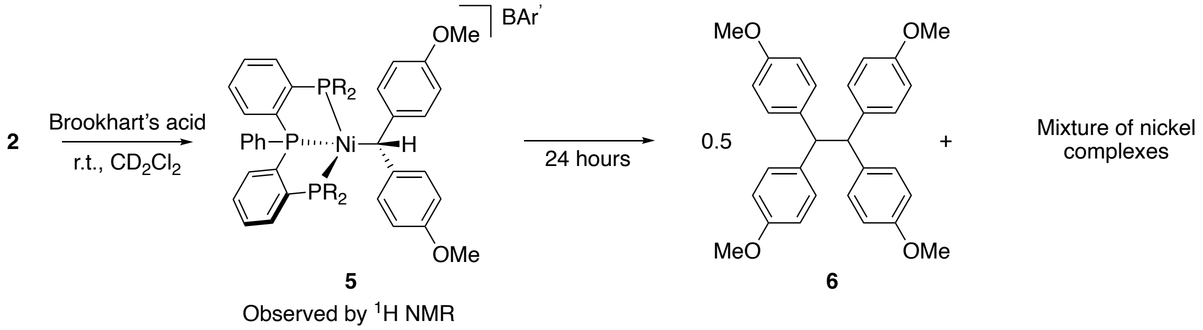

In a 20 mL vial, 5 mg of complex **2** (0.005 mmol) were dissolved in 0.4 mL of CD_2Cl_2. To this solution, 5 mg of $[H(Et_2O)]^+[[3,5\text{-}(CF_3)_2C_6H_3]_4B]^-$ (0.005 mmol) dissolved in 0.4 mL of CD_2Cl_2 were added, the resulting solution was then transferred to a J-Young NMR tube for NMR analysis.

The ^{31}P NMR spectrum showed the presence of at least 3 complexes from which the major compound is characterized by:

^{31}P NMR (162 MHz, THF-d_8) δ 82.09 (s, 1P), 47.83 (d, J = 170.5 Hz, 1P), 35.75 (d, J = 168.5 Hz, 1P).

We suggest that these signals correspond to the alkyl complex **5**. The ^{1}H NMR spectrum, despite its complexity, shows a clear signal for the alkyl ligand at 4.93 ppm (dd, J = 14.6, 7.3 Hz). We support this hypothesis by selective ^{31}P decoupling analysis shown in figure S27.

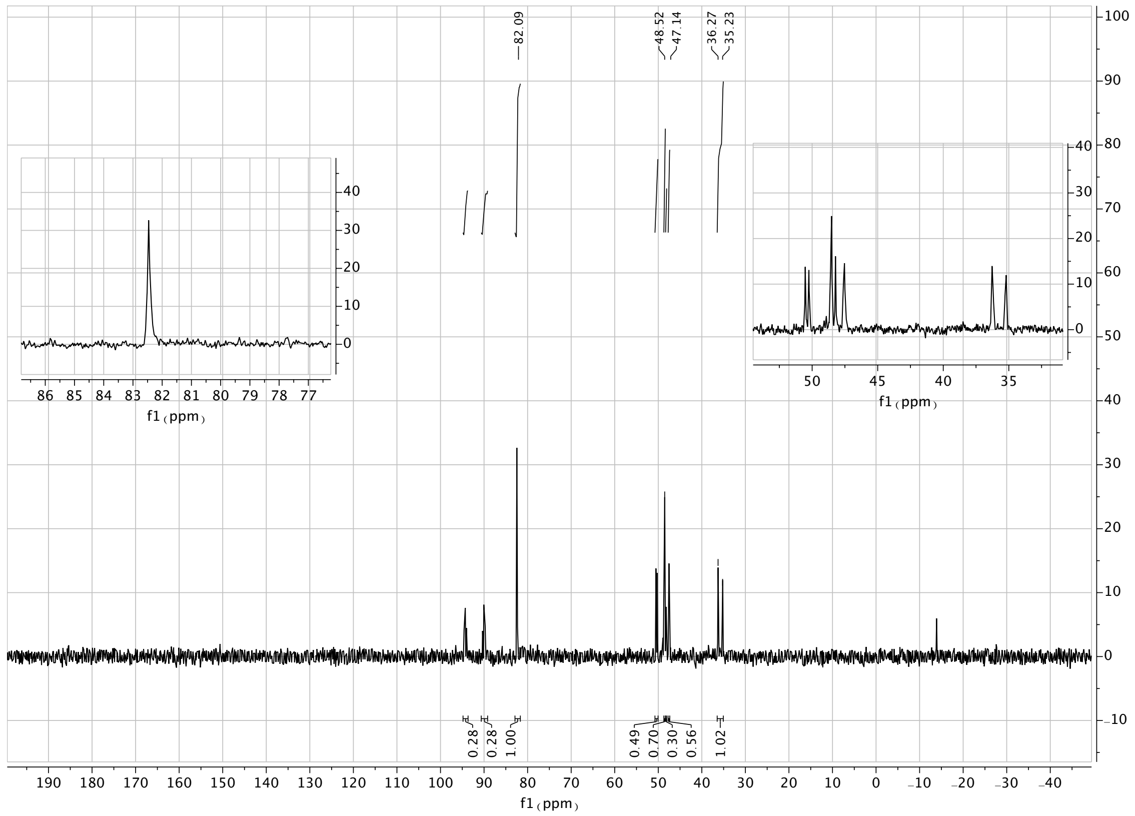

Figure S27: ^{31}P NMR spectrum for the reaction between complex **2** and HBArF in CD_2Cl_2 at room temperature.

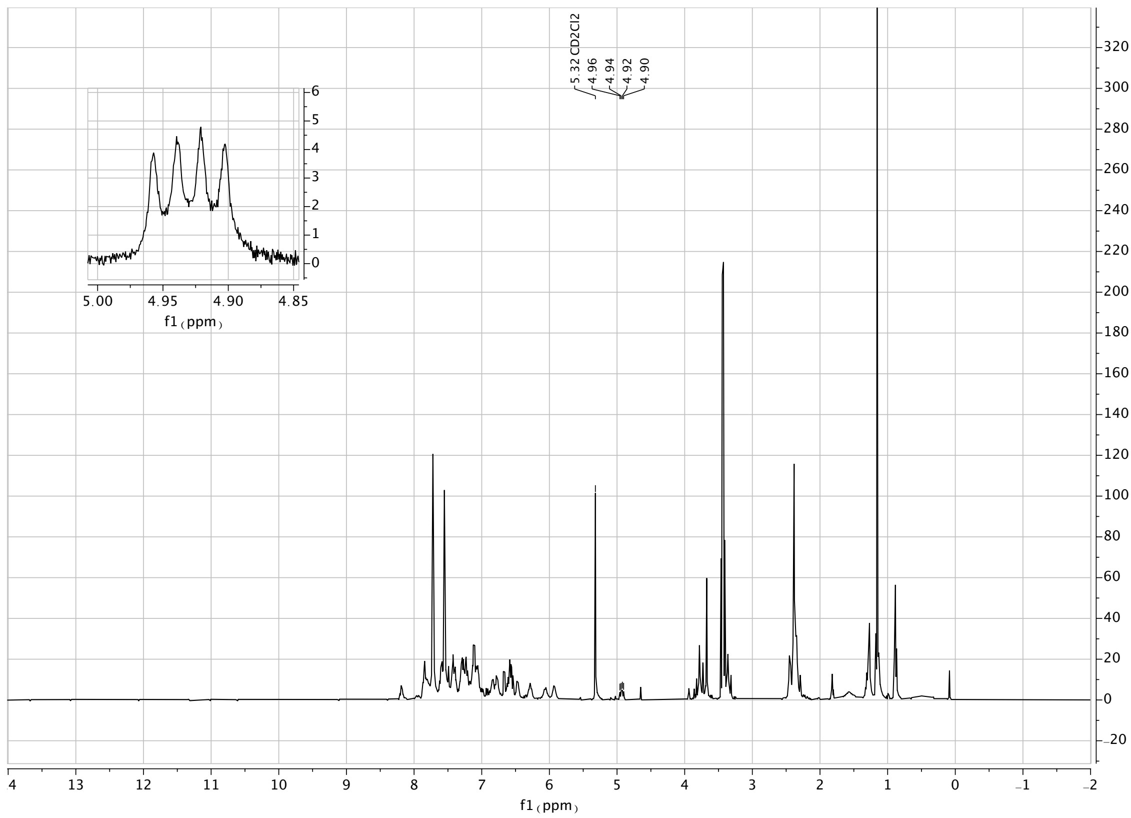

Figure S28: ^{1}H NMR spectrum for the reaction between complex **2** and HBArF in CD_2Cl_2 at room temperature.

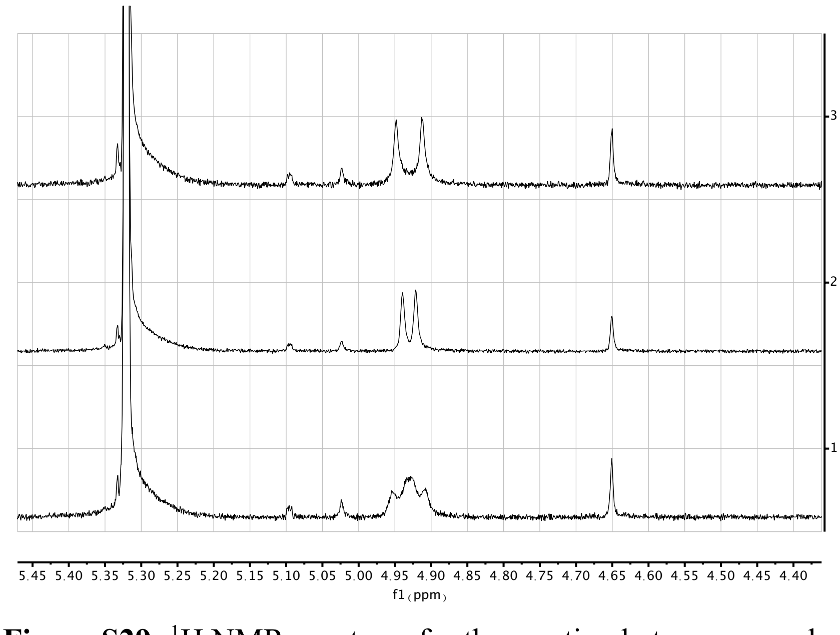

Figure S29: ^{1}H NMR spectrum for the reaction between complex **2** and HBArF with a focalized decoupling with ^{31}P signals at 82, 47 and 35 ppm (from bottom to the top) in CD_2Cl_2 at room temperature.

Reaction of complex 2 with HCl dissolved in ether:

In a 20 mL vial, 5 mg of complex **2** (0.005 mmol) were dissolved in 0.8 mL of d_8-THF, and the resulting solution was transferred to a J-Young NMR tube. Next, the solution was cooled at -78ºC and 5 µL of a 1 M HCl solution in ether was added. The solution immediately changed from black to red and was submitted to NMR analysis. The reaction mixture was also submitted to EPR analysis. For this purpose, 5 drops of the solution were diluted in 1 mL of toluene before measuring the EPR spectrum.

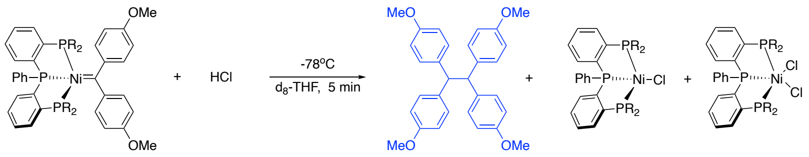

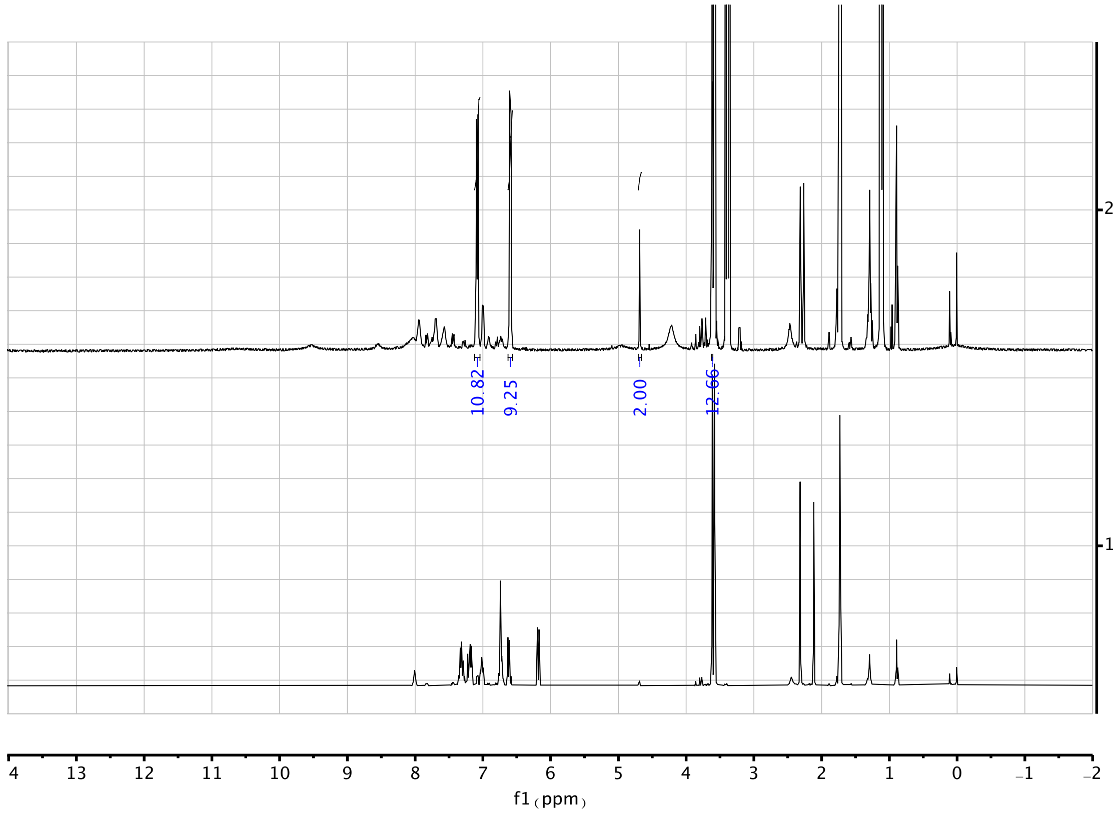

Figure S30: ^{1}H NMR spectra comparison of complex **2** (bottom, C_6D_6 at room temperature) with the reaction between complex **2** and HCl (top, d_8-THF at room temperature) where the integrated signals correspond to the organic homocoupling product 1,1,2,2-tetrakis(4-methoxyphenyl)ethane.

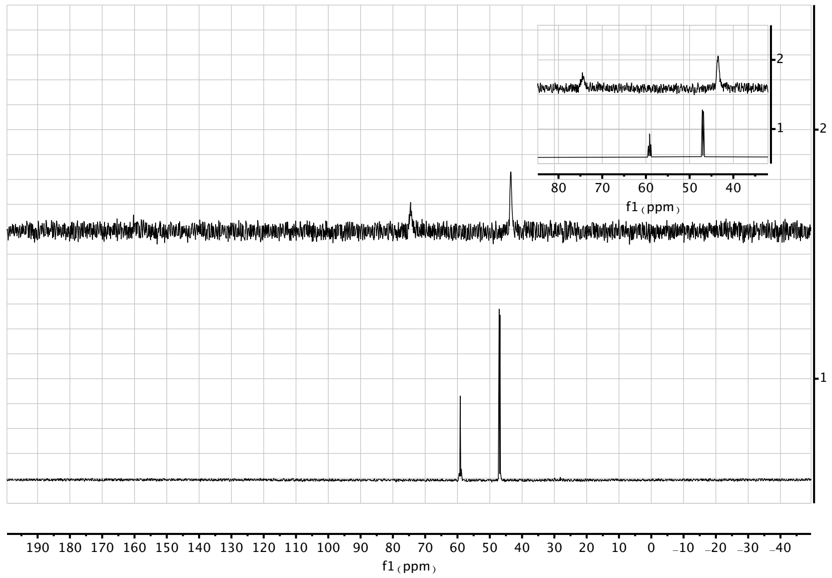

Figure S31: ^{31}P {^{1}H} NMR spectra comparison of complex **2** (bottom, C_6D_6 at room temperature) with the reaction between complex **2** and HCl (top, d_8-THF at room temperature). The phosphorous signals observed after reaction correspond to complex PPP$^{p\text{-tol}}$NiCl$_2$ (**9**).

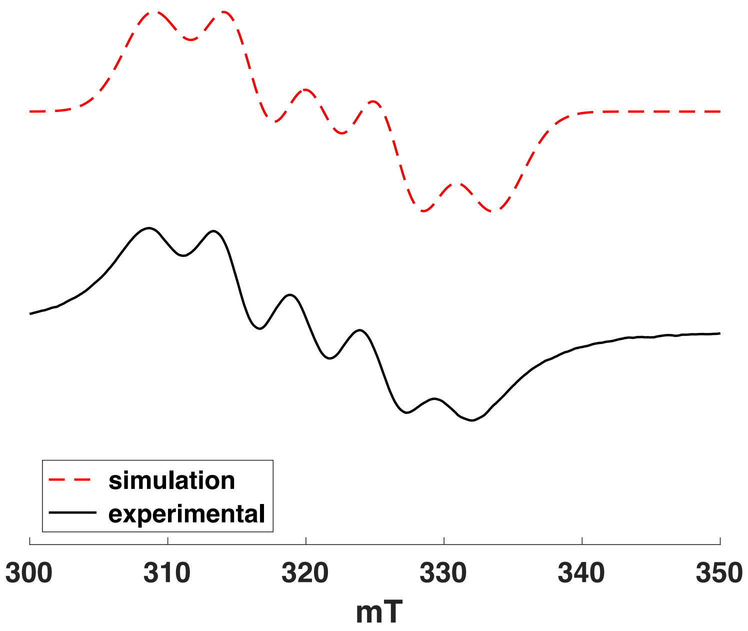

Figure S32: EPR spectrum at room temperature for the reaction between complex **2** and HCl indicating the presence of complex **8**. The EPR spectrum was modeled with the Matlab toolbox EasySpin,[10] giving a g value of 2.09 and (300, 150, 150) MHz as hyperfine coupling values.

Synthesis of complex ($PPP^{p\text{-tol}}$)$Ni^{I}Cl$ (8) for the comparison of the EPR spectrum:

50 mg of ligand $PPP^{p\text{-tol}}$ (0.07 mmol) and 10 mg of $Ni(COD)_2$ (0.04 mmol) were added to a 20 mL vial. The mixture was dissolved in 2 mL of THF and stirred at room temperature for 5 minutes. The resulting deep red solution was slowly added under stirring to a 3 mL $NiCl_2$(glyme) (8 mg, 0.04 mmol) THF suspension, and the reaction mixture was stirred for 1 hour. The solution was filtered through glass fiber paper and the THF was almost completely evaporated. Addition of hexane (5 mL) afforded a red precipitate, which was recovered by filtration and dried under vacuum. Yield: 47 mg (89 %). A small sample of the compound was dissolved in 1 mL of toluene and its EPR spectrum was measured.

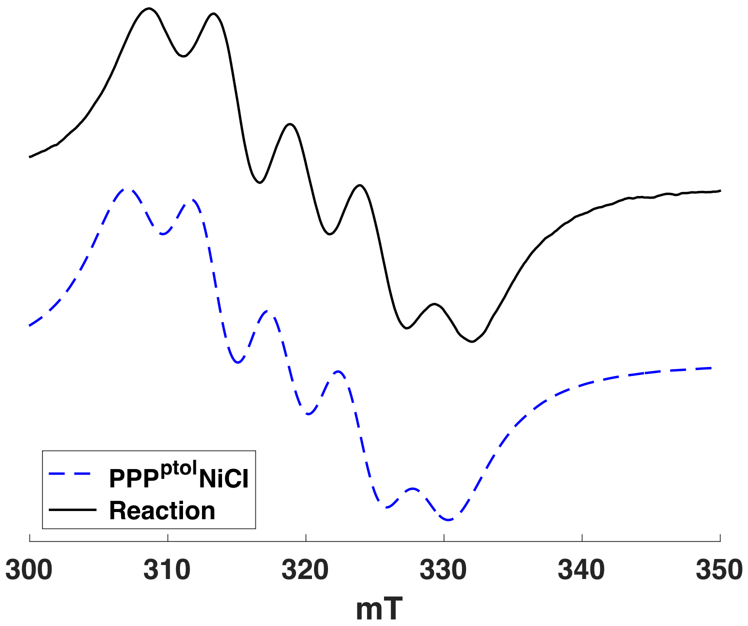

Figure S33: EPR spectrum at room temperature for the reaction between complex **2** and HCl indicating the presence of complex **8** (black line). EPR spectrum for a pure sample of complex **8** (blue dashed line).

COMPUTATIONAL SECTION

General information

DFT calculations were performed using the Gaussian 16 software package version C.01.[11] In the case of nickel carbene **2**, *p*-tolyl and *p*-anisyl groups were substituted by phenyl groups. Geometry optimizations were carried out in vacuum at the M06L-D3/6-31g(d,p)[12] level of theory on all atoms. Frequency analyses on all stationary points were used to ensure that they are minima (no imaginary frequency). ΔG^{o} was calculated by single point calculation at M06L-D3/def2TZVP level of theory adjusting the value with the thermal correction obtained at the M06L-D3/6-31g(d,p) level of theory with temperature 298.15 K and pressure 1 atmosphere. QTAIM analysis was performed on the electron density extracted from the single point calculation at M06L-D3/def2TZVP level of theory using the Multiwfn program.[13] Energy Decomposition Analysis (EDA) was performed at ZORA-BLYP-D3(BJ)/TZ2P//M06L-D3/6-31g(d,p)[12,14] level of theory using ADF.[15] For (dtpbe)$NiCPh_2$ and (PPP^{Ph})$NiCPh_2$, EDA was

performed on the optimized structures. For the analysis of simplified structures (Figure 4, Figure S42 and S43), substituents were deleted or/and were replaced by protons using standard P–H and C–H bond lengths except for structures **J** and **K** (see figure S44) and without geometry optimization. In the case of the structure **J**, C=C distance was adjusted to represent olefins (non-aromatic) in the backbone and in structure **K**, the position of the phosphine of the apical position was changed to depict the phosphine lone pair in opposite position to nickel be structure **J**. Additionally, Ni–C distance of P^1-Ni-C^1-C^2 dihedral angle were changed if indicated in Figure S42 and S43.

DFT calculations

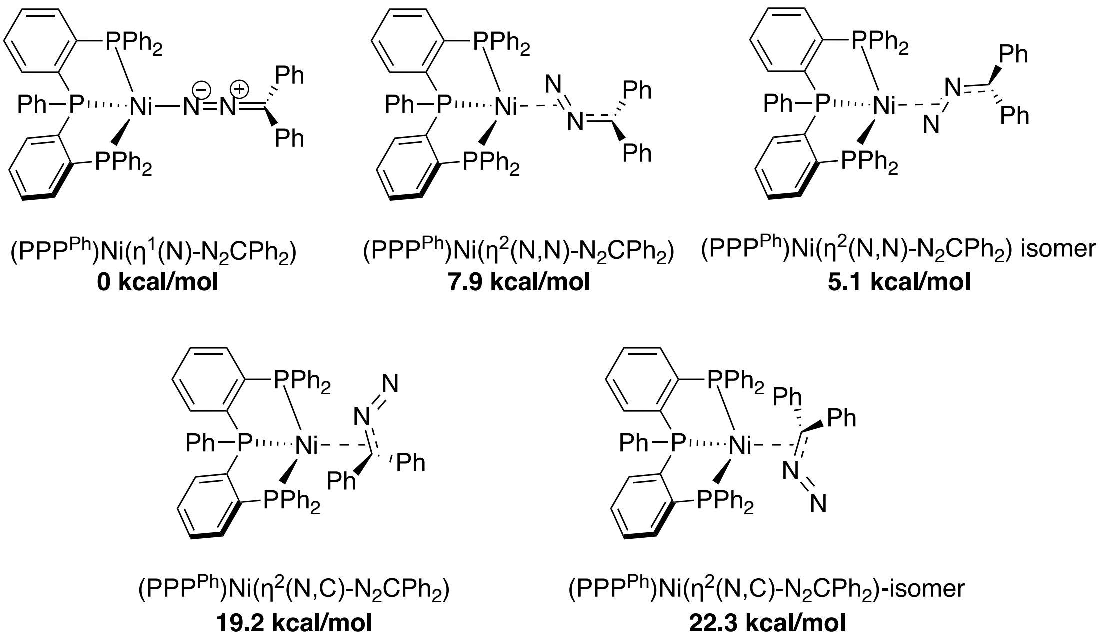

Figure S34: Different coordination modes between diazoalkane and (PPP^{Ph})Ni, where the Gibbs free energy is reported with respect to the most stable coordination mode, computed at M06L-D3/def2TZVP//M06L-D3/6-31g(d,p).

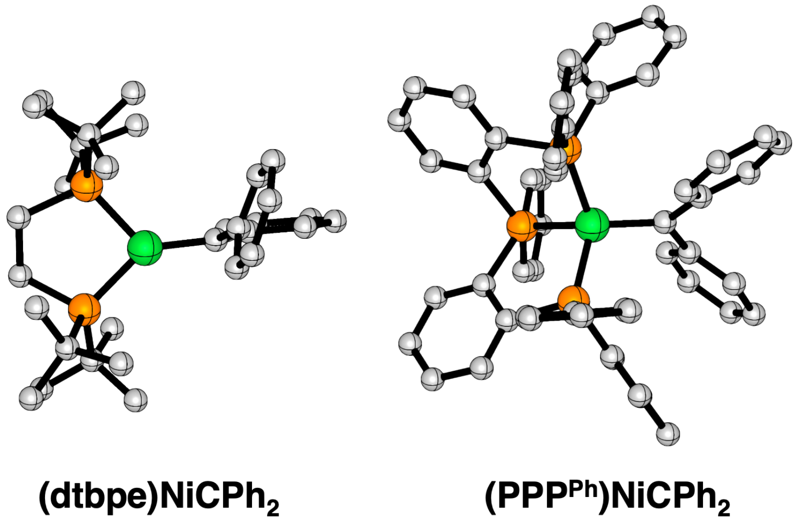

Figure S35. Optimized structures of the Nickel–Carbene complexes studied in this work computed at M06L-D3/6-31g(d,p) level of theory.

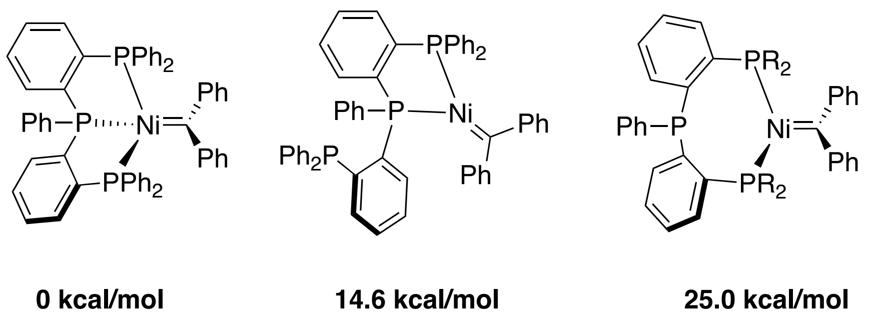

Figure S36: Gibbs free energy of (PPP$^{p\text{-tol}}$)NiC(Ph)$_2$ **2** with different coordination modes.

Table S1: Bond dissociation Gibbs free energies (ΔG_{BDE}; in kcal mol^{-1}), bond dissociation enthalpies (ΔH_{BDE}; in kcal mol^{-1}), bond dissociation energies (ΔE_{BDE}; in kcal mol^{-1}) and acitvation strain analysis of the Ni–Carbene bond of (dtbpe)NiCPh$_2$ and (PPP^{Ph})NiCPh$_2$.[a,b,c]

Structure	ΔG_{BDE}	ΔH_{BDE}	ΔE_{BDE}	ΔE_{strain}	ΔE_{int}
**(dtbpe)NiCPh$_2$**	71.2 (68.3)	84.1 (81.1)	86.2 (83.2)	12.4 (12.0)	–98.6 (–95.2)
**(PPP^{Ph})NiCPh$_2$**	56.9 (52.5)	72.3 (67.8)	74.1 (69.6)	9.8 (9.5)	–83.9 (–79.1)

[a] Computed at ZORA-BLYP-D3(BJ)/TZ2P//M06L-D3/6-31g(d,p). [b] Values in parenthesis are computed at M06L-D3/def2TZVP//M06L-D3/6-31g(d,p). [c] $\Delta E_{BDE} = -\Delta E$.

Table S2: Single point energies and thermal corrections of optimized structures obtained at the M06L-D3/def2TZVP//M06L-D3/6-31g(d,p) level of theory

	**Energy (Hartree)**	**Thermal correction to free energy**	**Thermal correction to enthalpy**
**(PPP^{Ph})Ni[η^1(N)-N$_2$CPh$_2$]**	-4764.206417	0.730302	0.872964
**(PPP^{Ph})Ni[η^2(N,N)-N$_2$CPh$_2$]**	-4764.194738	0.731252	0.731252
**(PPP^{Ph})Ni[η^2(N,N)-N$_2$CPh$_2$] isomer**	-4764.198633	0.730735	0.730735
**(PPP^{Ph})Ni[η^2(C,N)-N$_2$CPh$_2$]**	-4764.177007	0.731525	0.731525
**(PPP^{Ph})Ni[η^2(C,N)-N$_2$CPh$_2$] isomer**	-4764.171814	0.731198	0.731198
**(PPP^{Ph})NiCPh$_2$**	–4654.648228	0.723451	0.860999
**(PPP^{Ph})NiCPh$_2$, without side phosphine**	–4654.608586	0.723724	0.861005
**(PPP^{Ph})NiCPh$_2$, without central phosphine**	–4654.620373	0.718841	0.861015
**(dtbpe)NiCPh$_2$**	-3402.743419	0.671338	0.780625
**CPh$_2$**	-501.395951	0.14833	0.195656
**(dtbpe)Ni**	-2901.214894	0.14833	0.581625
**(PPP^{Ph})Ni**	-4153.141378	0.547839	0.662527
**(PPP^{Ph})Ni(*p*-anisyl)$_2$**	-4883.75890	0.782383	0.931338

QTAIM comparison of reported Ni carbenes

QTAIM was used to define the nature of the Ni–C bond interaction.[16] Visual inspection of the Laplacian map on the plane of the carbene ligand does not show significant differences between Fischer and Schrock carbenes (it mostly depicts the contour lines of the =C(C)$_2$ atoms). Nevertheless, the perpendicular plane [p(π) orbital plane] of the carbene shows a significant difference for each type of carbene. For the donor–acceptor interaction characteristic of a Fischer carbene, an area of depleted charge concentration (a hole) is observed at the carbene carbon atom. This is observed in Figure S36 and Figure S37[8] and the area of charge depletion is marked by pink arrows. In the case of a delocalized interaction characteristic of a Schrock carbene, the visual inspection shows the charge concentration to be continuous around the carbon atom, as also discussed by Piers and co-workers in Figure S38.[17] The comparison of the three discussed Laplacian maps at the carbene carbon atoms is depicted in Figure S39. Note that these differences between Fischer and Schrock carbenes, as illustrated by the Laplacian maps are fully consistent with the work of Frenking, Solà, and Vyboishchikov.[16]

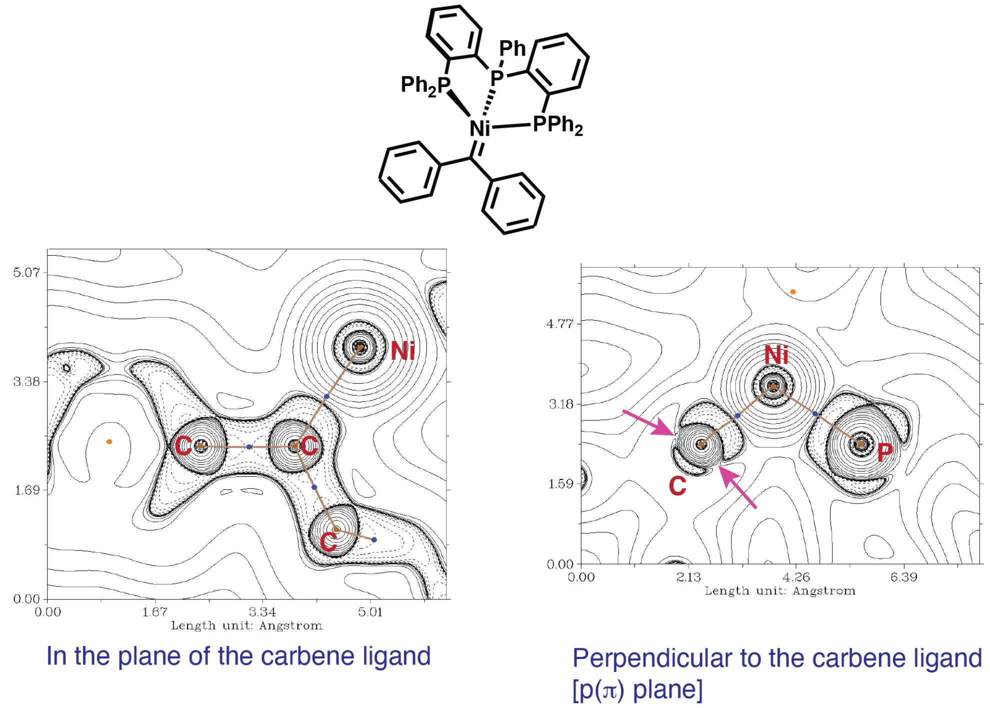

Figure S37: Two different views of the Laplacian map with the positive area (ionic bond area; solid lines) and negative area (covalent bond area; dashed lines) from the QTAIM topological analysis of (PPP^{Ph})NiCPh$_2$. Blue dots represent bonding critical points (BCPs) and orange dots Ring critical points (RCPs). Pink arrows on the p(π) plane point at an area of charge depletion.

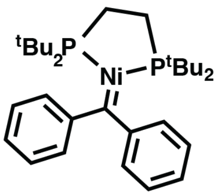

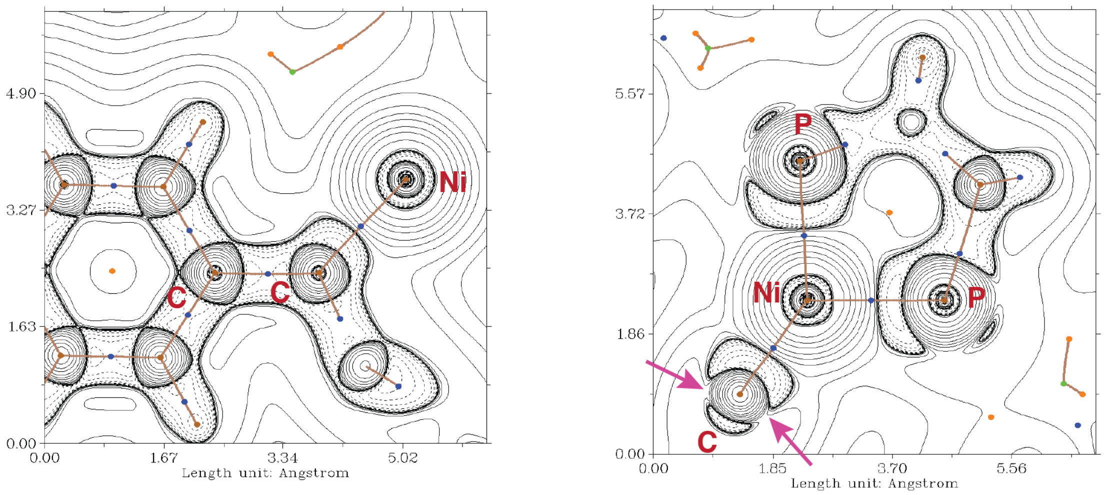

In the plane of the carbene ligand

Perpendicular to the carbene ligand [p(π) plane]

Figure S38: Two different views of the Laplacian map with the positive area (ionic bond area; solid lines) and negative area (covalent bond area; dashed lines) from the QTAIM topological analysis of (dtpbe)NiCPh$_2$.[8] Blue dots represent bonding critical points (BCPs) and orange dots Ring critical points (RCPs). Pink arrows on the p(π) plane point at an area of charge depletion.

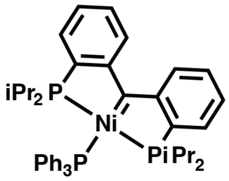

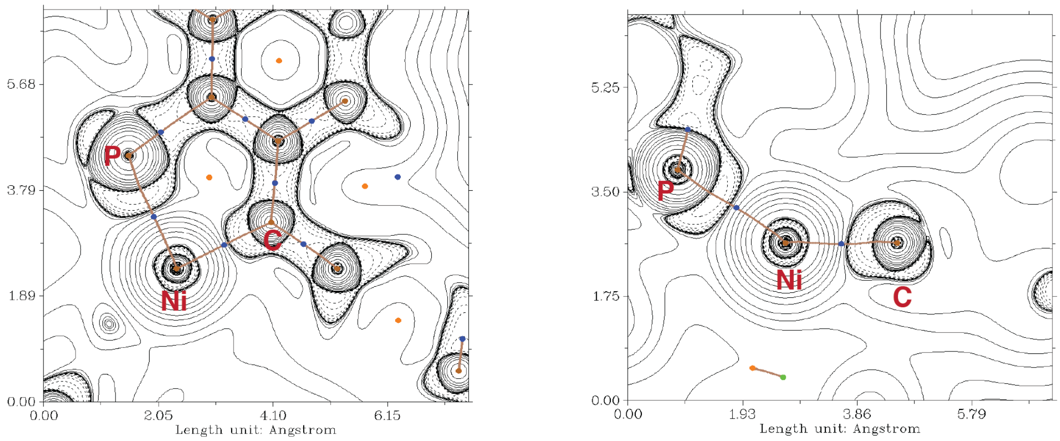

In the plane of the carbene ligand

Perpendicular to the carbene ligand [p(π) plane]

Figure S39: Two different views of the Laplacian map with the positive area (ionic bond area; solid lines) and negative area (covalent bond area; dashed lines) from the QTAIM topological analysis of (PCcarbeneCP)NiPPh$_3$.[17] Blue dots represent bonding critical points (BCPs) and orange dots Ring critical points (RCPs).

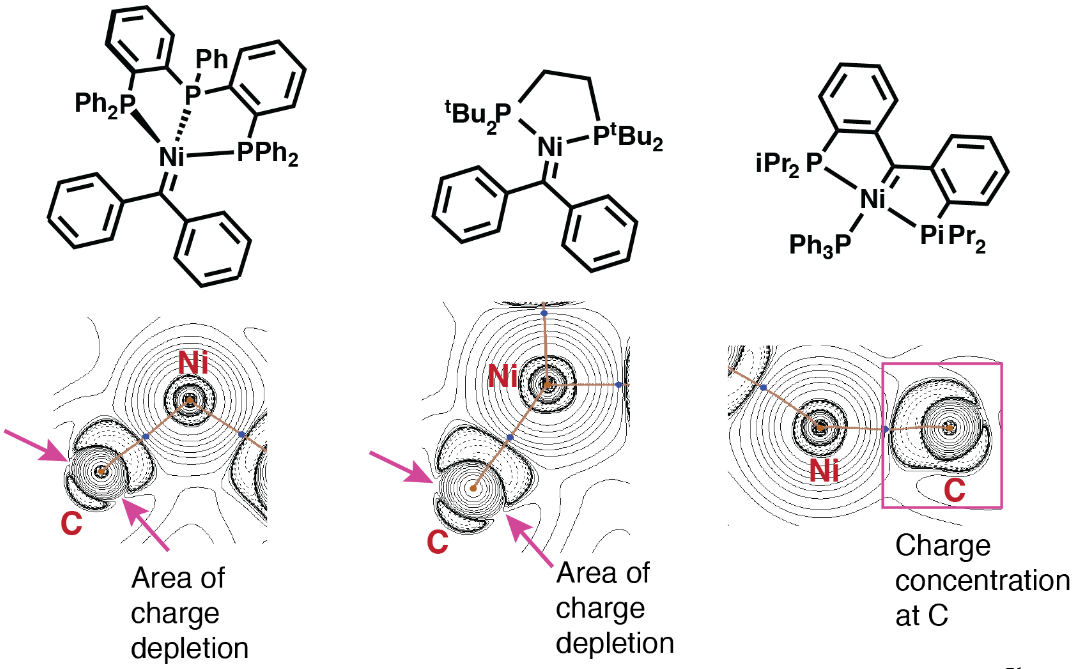

Figure S40: Zoomed images at the carbene carbon from the Laplacian maps of $(PPP^{Ph})NiCPh_2$ (left, Figure S36), $(dtpbe)NiCPh_2$[8] (middle, Figure S37) and $(PCcarbeneCP)NiPPh_3$[17] (right, Figure S38).

Additional notes of the EDA analysis

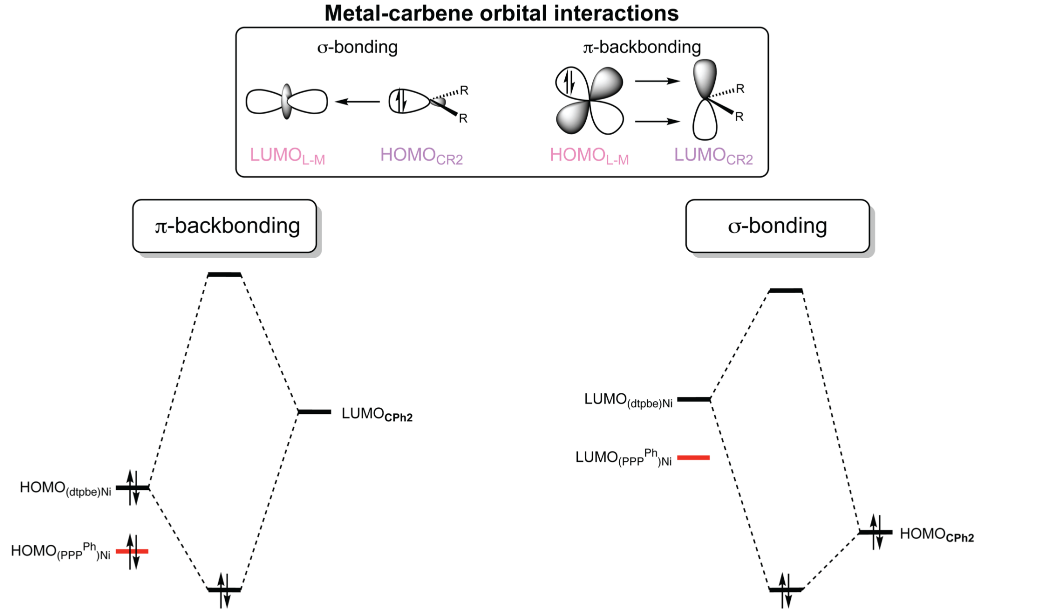

System	⟨HOMO|LUMO⟩	ε HOMO	Mul. pop. HOMO	Mul. pop. LUMO
(dtpbe)$NiCPh_2$	0.15	–2.3	1.17	0.80
(PPP^{Ph})$NiCPh_2$	0.10	–3.0	1.48	0.64

System	⟨LUMO|HOMO⟩	ε LUMO	Mul. pop. LUMO	Mul. pop. HOMO
(dtpbe)$NiCPh_2$	0.35	–1.5	0.46	1.50
(PPP^{Ph})$NiCPh_2$	0.17	–1.9	0.22	1.50

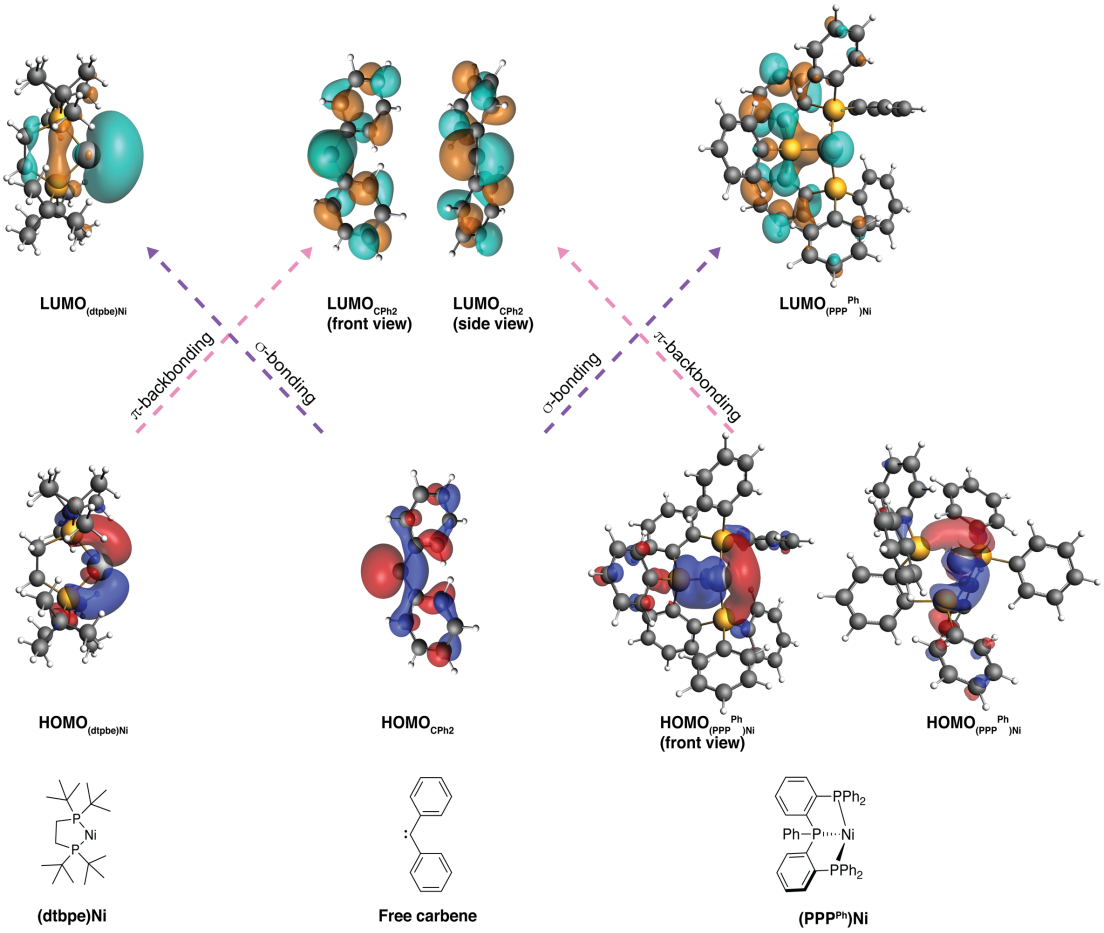

Figure S41. Molecular orbital analyses of the π-backbonding and σ-bonding between the Nickel complex and carbene of the Ni–carbene complexes, computed at ZORA-BLYP-D3(BJ)/TZ2P//M06L-D3/6-31g(d,p).

Table S3. Energy decomposition analyses (in kcal mol^{-1}) of the Ni–carbene complexes and Ni–carbene bond length (in Å).[a]

**Complex**	ΔE_{int}	ΔV_{elstat}	ΔE_{Pauli}	ΔE_{oi}	ΔE_{disp}	***r*****(Ni•••C)**
**(PPP^{Ph})NiC(*p*-anisyl)$_2$**	–82.6	–177.0	225.7	–91.3	–39.9	1.856

[a] Computed at ZORA-BLYP-D3(BJ)/TZ2P//M06L-D3/6-31g(d,p).

To study the influence of the phosphine in apical position in the EDA analysis in (dtpbe)NiCPh$_2$, we analyze different simplified structures related to both (dtpbe)NiCPh$_2$ and (PPP^{Ph})NiCPh$_2$. In Figure S38, Structure **A** represents the simplified version of (dtpbe)NiCPh$_2$ with a ΔE_{int} of –87.6 kcal/mol.Twisting the P1-Ni-C1-C2 dihedral angle from 88º to 65º to resemble the dihedral angle of (PPP^{Ph})NiCPh$_2$ results in structure **D**. **D** presents a slightly decrease of ΔE_{int} (–84.7 kcal mol^{-1}) due to a loss of stabilizing orbital overlap (Figure 3). Enlongation of the Ni–C bond from 1.805 Å to 1.854 Å to resemble (PPP^{Ph})NiCPh$_2$ results in structure **E**. This structure does not yield significant changes in ΔE_{int} (–84.8 kcal mol^{-1}). Increase of the P-Ni-P angle from 92.5º to 125º to resemble (PPP^{Ph})NiCPh$_2$ results in structure **B**. In structure **B**, a substantional change in the ΔE_{int} (–62.1 kcal mol^{-1}) is observed as a consequence of the steric repulsion between the phosphines and the carbene. Finally, having the phosphine in apical position in structure **C**, results in the a favorable change of ΔE_{int} (–71.8 kcal mol^{-1}) that is evident by the decrease of destabilizing ΔE_{Pauli}. To corroborate that the simplified structures are representative of (dtpbe)NiCPh$_2$, first we replace the *t*-butyl substituents by protons (**F**, Figure S39). Subsequently, the ethane linker is replaced by protons resulting in **A**. In Table S2 the EDA is shown, the same trend is observed after simplifying the structures without significant changes.

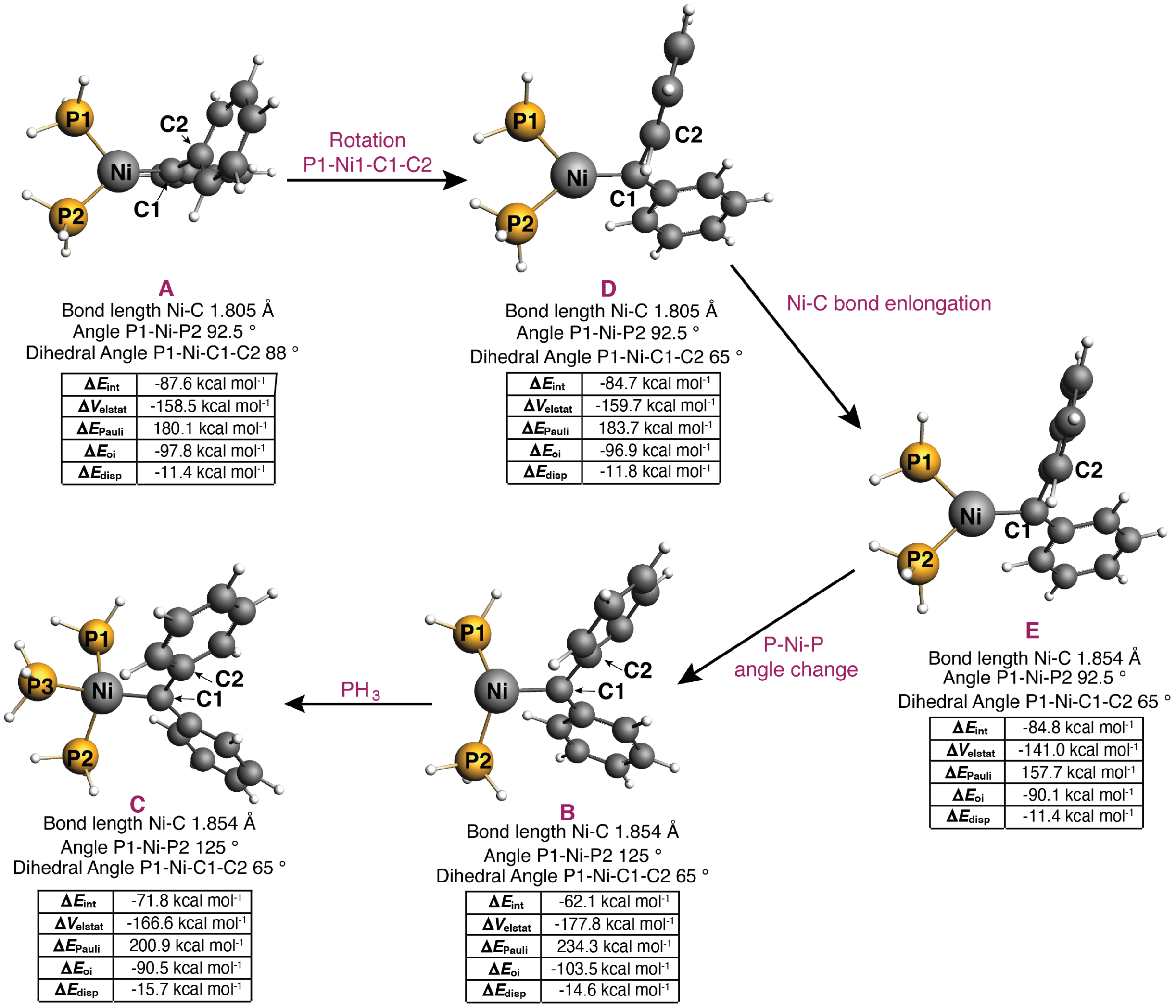

A

ΔE_{int}	-87.6 kcal mol^{-1}
ΔV_{elstat}	-158.5 kcal mol^{-1}
ΔE_{Pauli}	180.1 kcal mol^{-1}
ΔE_{oi}	-97.8 kcal mol^{-1}
ΔE_{disp}	-11.4 kcal mol^{-1}

D

ΔE_{int}	-84.7 kcal mol^{-1}
ΔV_{elstat}	-159.7 kcal mol^{-1}
ΔE_{Pauli}	183.7 kcal mol^{-1}
ΔE_{oi}	-96.9 kcal mol^{-1}
ΔE_{disp}	-11.8 kcal mol^{-1}

E

ΔE_{int}	-84.8 kcal mol^{-1}
ΔV_{elstat}	-141.0 kcal mol^{-1}
ΔE_{Pauli}	157.7 kcal mol^{-1}
ΔE_{oi}	-90.1 kcal mol^{-1}
ΔE_{disp}	-11.4 kcal mol^{-1}

C

ΔE_{int}	-71.8 kcal mol^{-1}
ΔV_{elstat}	-166.6 kcal mol^{-1}
ΔE_{Pauli}	200.9 kcal mol^{-1}
ΔE_{oi}	-90.5 kcal mol^{-1}
ΔE_{disp}	-15.7 kcal mol^{-1}

B

ΔE_{int}	-62.1 kcal mol^{-1}
ΔV_{elstat}	-177.8 kcal mol^{-1}
ΔE_{Pauli}	234.3 kcal mol^{-1}
ΔE_{oi}	-103.5 kcal mol^{-1}
ΔE_{disp}	-14.6 kcal mol^{-1}

Figure S42. Energy decomposition analyses (in kcal mol^{-1}) of the $(PH_3)_2Ni$ and (PH3)$_3$Ni derived carbenes (CPh_2) computed at ZORA-BLYP-D3(BJ)/TZ2P//M06L-D3/6-31g(d,p) level of theory.

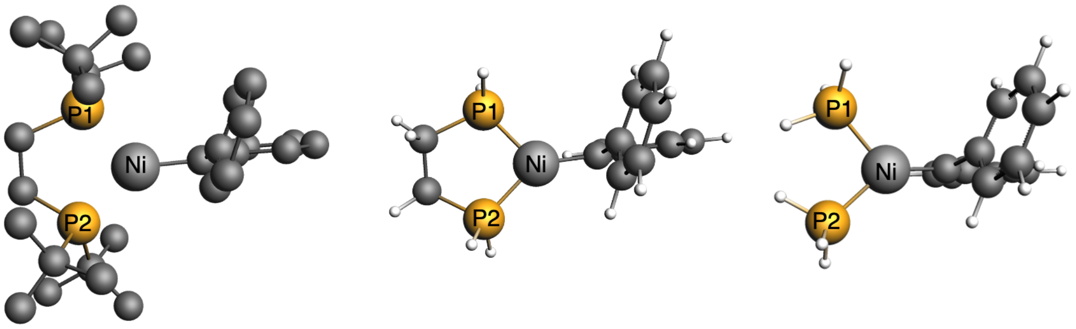

Figure S43. (dtpbe)$NiCPh_2$ and derived structures studied with EDA.

Table S4: EDA (in kcal/mol) of the structures presented in Figure S38 computed at ZORA-BLYP-D3(BJ)/TZ2P//M06L-D3/6-31g(d,p) level of theory

	ΔE_{int}	ΔV_{elstat}	ΔE_{Pauli}	ΔE_{oi}	ΔE_{disp}
**(dtpbe)NiCPh₂**	–98.6	–69.1	207.5	–112.3	–24.7
**F**	–86.4	–159.2	182.8	–98.3	–11.7
**A**	–87.6	–158.5	180.1	–97.8	–11.4

The same procedure is followed for (PPP^{Ph})$NiCPh_2$, which is a more complex structure and hence several additional intermediate structures are analyzed to guarantee that $(PH_3)_3NiCPh_2$ is representative of (PPP^{Ph})$NiCPh_2$ (Figure S40, Table S3). Note that no significant changes are observed when the structure is simplified. First, in structure **G** the phenyl group of the phosphine in apical position is replaced by a proton. In Structure **H**, the phenyl groups of the side phosphine arms are replaced by protons without changing the substituent in apical position. Structure **I** has all the phenyl substituents of the phosphines replaced by protons; once again no significant changes are observed. For structure **J**, the aromatic rings of the backbone are deleted, the C=C distance is changed to represent an olefin; comparison with the structure **C** $(PH_3)_3NiCPh_2$ shows the backbone does not have an effect in the chemical bonding of the fragments. Additionally, structure **J** is minimally modified to represent the proximity of a phosphine in the backbone without coordination resulting in structure **K**. EDA shows the stabilization is observed if the phosphine is coordinated and not only by proximity.

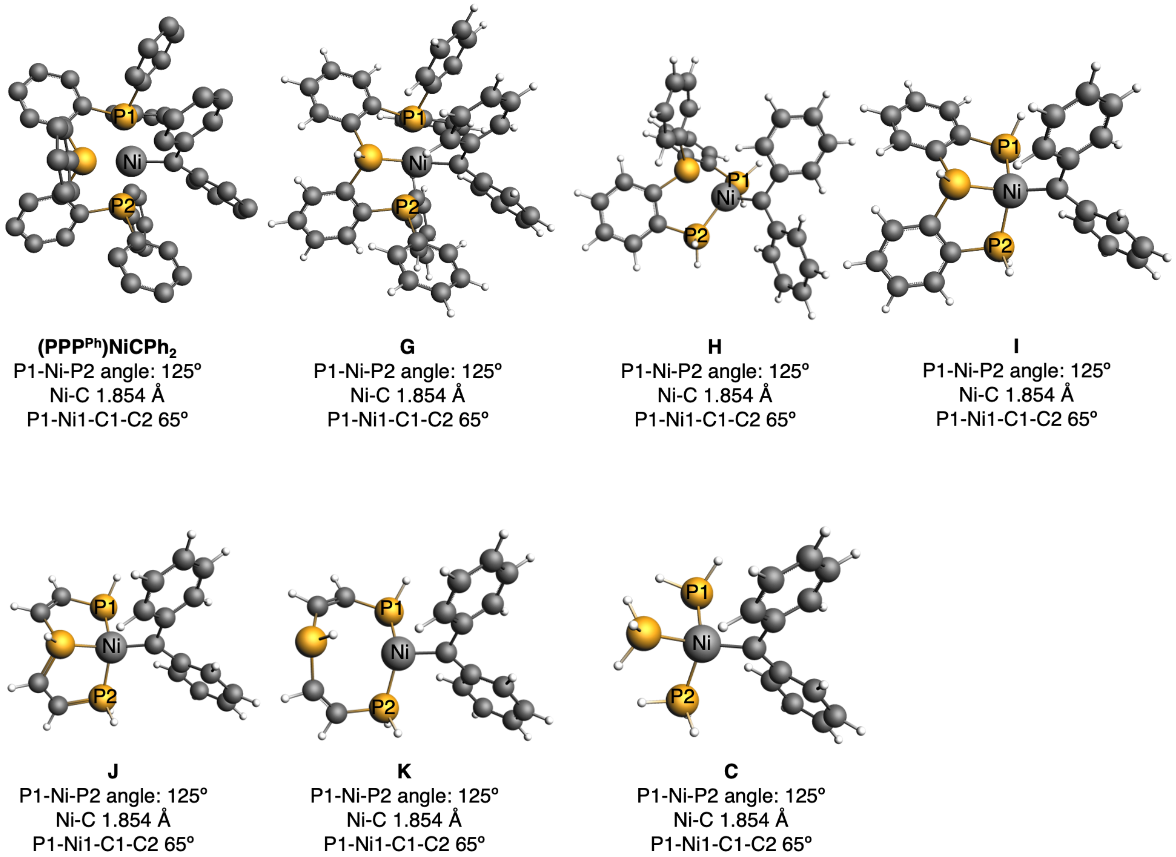

Figure S44: $(PPP^{Ph})NiCPh_2$ and derived structures studied with EDA.

Table S5: EDA (in kcal/mol) of the structures presented in Figure S39 computed at ZORA-BLYP-D3(BJ)/TZ2P//M06L-D3/6-31g(d,p) level of theory

	ΔE_{int}	ΔV_{elstat}	ΔE_{Pauli}	ΔE_{oi}	ΔE_{disp}
**$(PPP^{Ph})NiCPh_2$**	–83.9	–176.0	223.9	–94.2	–37.2
**G**	–82.3	–174.5	221.5	–94.3	–35.0
**H**	–73.1	–168.9	205.4	–91.2	–18.4
**I**	–71.7	–167.1	202.6	–91.3	–15.9
**J**	–71.0	–168.8	205.9	–92.1	–15.7
**K**	–63.7	–176.9	230.8	–101.8	–15.8
**C**	–71.8	–166.6	200.9	–90.5	–15.7

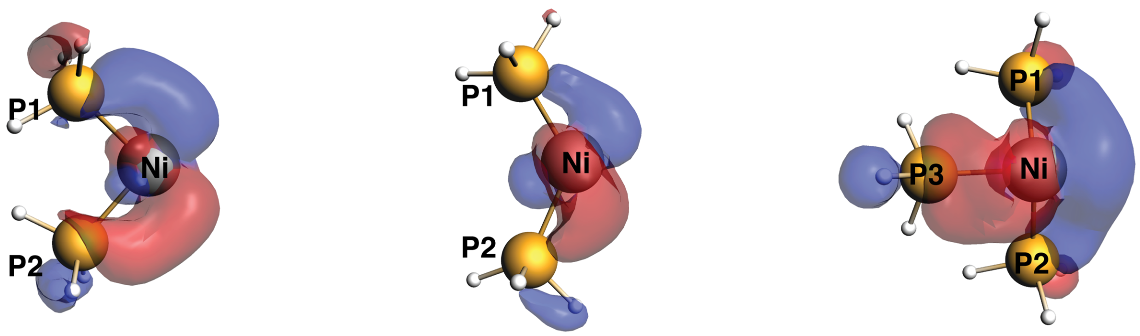

Figure S45: Molecular orbitals L-Ni (HOMO) of structure A to C.

Cartesian coordinates of optimized structures

Structures were optimized at the M06L-D3/6-31g(d,p) level of theory. Single point energies at M06L-D3/def2TZVP level of theory are reported.

$(PPP^{Ph})Ni[\eta^2(N)-N_2CPh_2]$
Energy: -4764.20641654 Hartree

Ni	-0.28192	-0.44604	-0.45947
P	-1.90838	-1.53226	0.40728
P	0.71687	-0.54151	1.47600
P	-1.66441	1.17933	-0.84273
C	-1.35284	-2.33548	1.95420
C	-0.13509	-1.85006	2.46090
C	0.36455	-2.36478	3.65941
C	-0.33134	-3.35952	4.33985
C	-1.53561	-3.84565	3.83184
C	-2.04908	-3.33251	2.64456
C	-3.11890	-0.27970	0.98852
C	-2.96871	1.01006	0.45204
C	-3.80658	2.03722	0.89489
C	-4.76556	1.78802	1.87267
C	-4.91530	0.50656	2.40072
C	-4.09868	-0.52717	1.95272
C	-2.92404	-2.76575	-0.45763
C	-2.48938	-4.09492	-0.55545
C	-3.18947	-5.00842	-1.33533
C	-4.32037	-4.60432	-2.04306
C	-4.74878	-3.28125	-1.96511
C	-4.05830	-2.36650	-1.17704
C	0.45858	0.95238	2.48994
C	1.32807	2.03904	2.31898
C	1.09709	3.23749	2.98388
C	-0.02047	3.38167	3.80593

C	-0.90722	2.31645	3.95621
C	-0.66885	1.10672	3.30800
C	2.48147	-0.96096	1.73102
C	3.27231	-0.43806	2.76217
C	4.61054	-0.80360	2.87880
C	5.17429	-1.69792	1.97196
C	4.39314	-2.23728	0.95239
C	3.05566	-1.87159	0.83517
C	-2.66973	0.94588	-2.36245
C	-3.78810	1.74028	-2.64710
C	-4.58727	1.46097	-3.74952
C	-4.27415	0.38719	-4.58376
C	-3.15809	-0.39946	-4.31645
C	-2.36029	-0.12390	-3.20766
C	-1.30882	2.96664	-0.83990
C	-1.16659	3.69657	-2.02696
C	-0.82345	5.04570	-1.98744
C	-0.61597	5.68267	-0.76534
C	-0.73210	4.95664	0.41913
C	-1.06650	3.60777	0.38243
H	1.31070	-1.99475	4.04979
H	0.06950	-3.76333	5.26539
H	-2.07166	-4.62979	4.35923
H	-2.98129	-3.72049	2.23785
H	-3.69175	3.04035	0.49045
H	-5.39882	2.59774	2.22469
H	-5.66447	0.31520	3.16383
H	-4.20709	-1.52729	2.36828
H	-1.59815	-4.40907	-0.01574
H	-2.84782	-6.03805	-1.39579
H	-4.86242	-5.31792	-2.65693
H	-5.62366	-2.95561	-2.52153
H	-4.39526	-1.33238	-1.12599
H	2.18859	1.94091	1.66022
H	1.78645	4.06629	2.84034
H	-1.78773	2.42221	4.58481
H	-1.36448	0.27940	3.43725
H	2.84379	0.26791	3.46909
H	5.21450	-0.38346	3.67870
H	4.82934	-2.92041	0.22840
H	2.45096	-2.27823	0.02608
H	-4.03418	2.57812	-1.99680
H	-5.45731	2.07757	-3.95849
H	-2.91025	-1.23609	-4.96366
H	-1.49542	-0.74383	-2.97451
H	-1.33614	3.20854	-2.98421
H	-0.72267	5.60150	-2.91558
H	-0.55695	5.43679	1.37888
H	-1.15513	3.04428	1.30892
H	6.22252	-1.97117	2.05537

H	-0.20451	4.32137	4.31996
H	-0.35468	6.73673	-0.73739
H	-4.90234	0.16698	-5.44262
N	0.93887	-0.60239	-1.74621
N	2.10386	-0.70087	-2.00499
C	3.29645	-0.13773	-1.83850
C	3.33726	1.16809	-1.16731
C	4.37084	1.50261	-0.27136
C	2.31888	2.11760	-1.37351
C	4.41316	2.75219	0.33801
H	5.13352	0.76225	-0.04376
C	2.36613	3.36605	-0.76551
H	1.49788	1.88175	-2.04741
C	3.42164	3.69938	0.08261
H	5.22403	2.98125	1.02560
H	1.57336	4.08270	-0.96296
H	3.45987	4.67941	0.55162
C	4.45268	-0.91304	-2.26301
C	4.32321	-2.28688	-2.55580
C	5.73310	-0.34414	-2.39538
C	5.41520	-3.04929	-2.94184
H	3.34087	-2.74704	-2.47041
C	6.82636	-1.11550	-2.77134
H	5.86576	0.71855	-2.21493
C	6.68267	-2.47423	-3.04268
H	5.27855	-4.10635	-3.15771
H	7.80155	-0.64355	-2.86352
H	7.53998	-3.07376	-3.33455

$(PPP^{Ph})Ni[\eta^2(N,N)\text{-}N_2CPh_2]$

Energy: -4764.19473848 Hartree

Ni	-0.28192	-0.44604	-0.45947
P	-1.90838	-1.53226	0.40728
P	0.71687	-0.54151	1.47600
P	-1.66441	1.17933	-0.84273
C	-1.35284	-2.33548	1.95420
C	-0.13509	-1.85006	2.46090
C	0.36455	-2.36478	3.65941
C	-0.33134	-3.35952	4.33985
C	-1.53561	-3.84565	3.83184
C	-2.04908	-3.33251	2.64456
C	-3.11890	-0.27970	0.98852
C	-2.96871	1.01006	0.45204
C	-3.80658	2.03722	0.89489
C	-4.76556	1.78802	1.87267
C	-4.91530	0.50656	2.40072
C	-4.09868	-0.52717	1.95272
C	-2.92404	-2.76575	-0.45763
C	-2.48938	-4.09492	-0.55545

C	-3.18947	-5.00842	-1.33533
C	-4.32037	-4.60432	-2.04306
C	-4.74878	-3.28125	-1.96511
C	-4.05830	-2.36650	-1.17704
C	0.45858	0.95238	2.48994
C	1.32807	2.03904	2.31898
C	1.09709	3.23749	2.98388
C	-0.02047	3.38167	3.80593
C	-0.90722	2.31645	3.95621
C	-0.66885	1.10672	3.30800
C	2.48147	-0.96096	1.73102
C	3.27231	-0.43806	2.76217
C	4.61054	-0.80360	2.87880
C	5.17429	-1.69792	1.97196
C	4.39314	-2.23728	0.95239
C	3.05566	-1.87159	0.83517
C	-2.66973	0.94588	-2.36245
C	-3.78810	1.74028	-2.64710
C	-4.58727	1.46097	-3.74952
C	-4.27415	0.38719	-4.58376
C	-3.15809	-0.39946	-4.31645
C	-2.36029	-0.12390	-3.20766
C	-1.30882	2.96664	-0.83990
C	-1.16659	3.69657	-2.02696
C	-0.82345	5.04570	-1.98744
C	-0.61597	5.68267	-0.76534
C	-0.73210	4.95664	0.41913
C	-1.06650	3.60777	0.38243
H	1.31070	-1.99475	4.04979
H	0.06950	-3.76333	5.26539
H	-2.07166	-4.62979	4.35923
H	-2.98129	-3.72049	2.23785
H	-3.69175	3.04035	0.49045
H	-5.39882	2.59774	2.22469
H	-5.66447	0.31520	3.16383
H	-4.20709	-1.52729	2.36828
H	-1.59815	-4.40907	-0.01574
H	-2.84782	-6.03805	-1.39579
H	-4.86242	-5.31792	-2.65693
H	-5.62366	-2.95561	-2.52153
H	-4.39526	-1.33238	-1.12599
H	2.18859	1.94091	1.66022
H	1.78645	4.06629	2.84034
H	-1.78773	2.42221	4.58481
H	-1.36448	0.27940	3.43725
H	2.84379	0.26791	3.46909
H	5.21450	-0.38346	3.67870
H	4.82934	-2.92041	0.22840
H	2.45096	-2.27823	0.02608
H	-4.03418	2.57812	-1.99680

H	-5.45731	2.07757	-3.95849
H	-2.91025	-1.23609	-4.96366
H	-1.49542	-0.74383	-2.97451
H	-1.33614	3.20854	-2.98421
H	-0.72267	5.60150	-2.91558
H	-0.55695	5.43679	1.37888
H	-1.15513	3.04428	1.30892
H	6.22252	-1.97117	2.05537
H	-0.20451	4.32137	4.31996
H	-0.35468	6.73673	-0.73739
H	-4.90234	0.16698	-5.44262
N	0.93887	-0.60239	-1.74621
N	2.10386	-0.70087	-2.00499
C	3.29645	-0.13773	-1.83850
C	3.33726	1.16809	-1.16731
C	4.37084	1.50261	-0.27136
C	2.31888	2.11760	-1.37351
C	4.41316	2.75219	0.33801
H	5.13352	0.76225	-0.04376
C	2.36613	3.36605	-0.76551
H	1.49788	1.88175	-2.04741
C	3.42164	3.69938	0.08261
H	5.22403	2.98125	1.02560
H	1.57336	4.08270	-0.96296
H	3.45987	4.67941	0.55162
C	4.45268	-0.91304	-2.26301
C	4.32321	-2.28688	-2.55580
C	5.73310	-0.34414	-2.39538
C	5.41520	-3.04929	-2.94184
H	3.34087	-2.74704	-2.47041
C	6.82636	-1.11550	-2.77134
H	5.86576	0.71855	-2.21493
C	6.68267	-2.47423	-3.04268
H	5.27855	-4.10635	-3.15771
H	7.80155	-0.64355	-2.86352
H	7.53998	-3.07376	-3.33455

$(PPP^{Ph})Ni[\eta^2(N,N)-N_2CPh_2]$ isomer

Energy: -4764.19863273 Hartree

Ni	-0.28192	-0.44604	-0.45947
P	-1.90838	-1.53226	0.40728
P	0.71687	-0.54151	1.47600
P	-1.66441	1.17933	-0.84273
C	-1.35284	-2.33548	1.95420
C	-0.13509	-1.85006	2.46090
C	0.36455	-2.36478	3.65941
C	-0.33134	-3.35952	4.33985
C	-1.53561	-3.84565	3.83184
C	-2.04908	-3.33251	2.64456

C	-3.11890	-0.27970	0.98852
C	-2.96871	1.01006	0.45204
C	-3.80658	2.03722	0.89489
C	-4.76556	1.78802	1.87267
C	-4.91530	0.50656	2.40072
C	-4.09868	-0.52717	1.95272
C	-2.92404	-2.76575	-0.45763
C	-2.48938	-4.09492	-0.55545
C	-3.18947	-5.00842	-1.33533
C	-4.32037	-4.60432	-2.04306
C	-4.74878	-3.28125	-1.96511
C	-4.05830	-2.36650	-1.17704
C	0.45858	0.95238	2.48994
C	1.32807	2.03904	2.31898
C	1.09709	3.23749	2.98388
C	-0.02047	3.38167	3.80593
C	-0.90722	2.31645	3.95621
C	-0.66885	1.10672	3.30800
C	2.48147	-0.96096	1.73102
C	3.27231	-0.43806	2.76217
C	4.61054	-0.80360	2.87880
C	5.17429	-1.69792	1.97196
C	4.39314	-2.23728	0.95239
C	3.05566	-1.87159	0.83517
C	-2.66973	0.94588	-2.36245
C	-3.78810	1.74028	-2.64710
C	-4.58727	1.46097	-3.74952
C	-4.27415	0.38719	-4.58376
C	-3.15809	-0.39946	-4.31645
C	-2.36029	-0.12390	-3.20766
C	-1.30882	2.96664	-0.83990
C	-1.16659	3.69657	-2.02696
C	-0.82345	5.04570	-1.98744
C	-0.61597	5.68267	-0.76534
C	-0.73210	4.95664	0.41913
C	-1.06650	3.60777	0.38243
H	1.31070	-1.99475	4.04979
H	0.06950	-3.76333	5.26539
H	-2.07166	-4.62979	4.35923
H	-2.98129	-3.72049	2.23785
H	-3.69175	3.04035	0.49045
H	-5.39882	2.59774	2.22469
H	-5.66447	0.31520	3.16383
H	-4.20709	-1.52729	2.36828
H	-1.59815	-4.40907	-0.01574
H	-2.84782	-6.03805	-1.39579
H	-4.86242	-5.31792	-2.65693
H	-5.62366	-2.95561	-2.52153
H	-4.39526	-1.33238	-1.12599
H	2.18859	1.94091	1.66022

H	1.78645	4.06629	2.84034
H	-1.78773	2.42221	4.58481
H	-1.36448	0.27940	3.43725
H	2.84379	0.26791	3.46909
H	5.21450	-0.38346	3.67870
H	4.82934	-2.92041	0.22840
H	2.45096	-2.27823	0.02608
H	-4.03418	2.57812	-1.99680
H	-5.45731	2.07757	-3.95849
H	-2.91025	-1.23609	-4.96366
H	-1.49542	-0.74383	-2.97451
H	-1.33614	3.20854	-2.98421
H	-0.72267	5.60150	-2.91558
H	-0.55695	5.43679	1.37888
H	-1.15513	3.04428	1.30892
H	6.22252	-1.97117	2.05537
H	-0.20451	4.32137	4.31996
H	-0.35468	6.73673	-0.73739
H	-4.90234	0.16698	-5.44262
N	0.93887	-0.60239	-1.74621
N	2.10386	-0.70087	-2.00499
C	3.29645	-0.13773	-1.83850
C	3.33726	1.16809	-1.16731
C	4.37084	1.50261	-0.27136
C	2.31888	2.11760	-1.37351
C	4.41316	2.75219	0.33801
H	5.13352	0.76225	-0.04376
C	2.36613	3.36605	-0.76551
H	1.49788	1.88175	-2.04741
C	3.42164	3.69938	0.08261
H	5.22403	2.98125	1.02560
H	1.57336	4.08270	-0.96296
H	3.45987	4.67941	0.55162
C	4.45268	-0.91304	-2.26301
C	4.32321	-2.28688	-2.55580
C	5.73310	-0.34414	-2.39538
C	5.41520	-3.04929	-2.94184
H	3.34087	-2.74704	-2.47041
C	6.82636	-1.11550	-2.77134
H	5.86576	0.71855	-2.21493
C	6.68267	-2.47423	-3.04268
H	5.27855	-4.10635	-3.15771
H	7.80155	-0.64355	-2.86352
H	7.53998	-3.07376	-3.33455

(PPP^{Ph})Ni[η^{2}(N,C)-N$_{2}$CPh$_{2}$]

Energy: -4764.17700704 Hartree

Ni	-0.17879	0.09436	-0.58244
P	0.63217	1.90760	0.32641

P	-1.39204	-0.03581	1.28079
P	1.74895	-0.89112	-0.15636
C	-0.62378	2.60349	1.46356
C	-1.60254	1.68469	1.88431
C	-2.61691	2.10001	2.74934
C	-2.67322	3.42396	3.17737
C	-1.70428	4.33475	2.75934
C	-0.67723	3.92594	1.91285
C	1.91848	1.30375	1.50265
C	2.33259	-0.03933	1.37074
C	3.15922	-0.59312	2.34991
C	3.59514	0.17616	3.42625
C	3.22498	1.51442	3.53052
C	2.38063	2.07111	2.57449
C	1.36076	3.34789	-0.50328
C	0.51595	4.27291	-1.13639
C	1.04454	5.30034	-1.90808
C	2.42374	5.41209	-2.07637
C	3.26985	4.49564	-1.45824
C	2.74397	3.47386	-0.67473
C	-0.57439	-0.85657	2.69944
C	-0.21022	-2.20108	2.54073
C	0.52555	-2.86579	3.51460
C	0.92458	-2.18740	4.66472
C	0.57704	-0.84930	4.83008
C	-0.16823	-0.18551	3.85830
C	-3.09766	-0.68632	1.28748
C	-3.43732	-1.93380	1.82526
C	-4.72542	-2.43911	1.67573
C	-5.69325	-1.70651	0.99246
C	-5.36814	-0.45852	0.46629
C	-4.08044	0.04818	0.60746
C	3.08906	-0.44464	-1.32537
C	4.20679	0.32631	-0.98872
C	5.15830	0.64985	-1.95345
C	5.01190	0.19941	-3.26189
C	3.91245	-0.58559	-3.60291
C	2.95944	-0.90182	-2.64323
C	2.08790	-2.68465	0.03579
C	3.36444	-3.20300	0.29471
C	3.56489	-4.57643	0.38415
C	2.49904	-5.45262	0.18171
C	1.23811	-4.95083	-0.12743
C	1.03587	-3.57515	-0.20267
H	-3.37062	1.38627	3.07497
H	-3.47519	3.74615	3.83552
H	-1.75045	5.36815	3.09200
H	0.07487	4.63889	1.58096
H	3.44984	-1.63813	2.28595
H	4.22372	-0.27595	4.18909

H	3.57348	2.11570	4.36568
H	2.05692	3.10653	2.66921
H	-0.56264	4.19118	-1.01117
H	0.37698	6.01122	-2.38682
H	2.83520	6.20876	-2.68929
H	4.34648	4.57299	-1.58557
H	3.41045	2.76332	-0.19404
H	-0.49749	-2.72155	1.63018
H	0.80473	-3.90543	3.36023
H	0.89803	-0.30960	5.71746
H	-0.41687	0.86400	3.99642
H	-2.69557	-2.51402	2.36646
H	-4.97303	-3.40890	2.09905
H	-6.11172	0.12415	-0.07045
H	-3.83450	1.01691	0.17645
H	4.34168	0.67182	0.03395
H	6.01973	1.25265	-1.67656
H	3.79047	-0.94272	-4.62148
H	2.09989	-1.51569	-2.90704
H	4.20967	-2.52607	0.40397
H	4.55739	-4.96537	0.59521
H	0.40823	-5.62450	-0.32417
H	0.05338	-3.18164	-0.46450
H	-6.69661	-2.10538	0.87280
H	1.51448	-2.69566	5.42255
H	2.65928	-6.52560	0.24327
H	5.75232	0.45705	-4.01387
N	0.47075	0.83366	-3.31252
N	-0.18843	0.33804	-2.46512
C	-1.42892	-0.28108	-2.11122
C	-1.45963	-1.73976	-2.32904
C	-2.39471	-2.53864	-1.64389
C	-0.55299	-2.38380	-3.18706
C	-2.40196	-3.91975	-1.79254
H	-3.11078	-2.05925	-0.98155
C	-0.55651	-3.76774	-3.32633
H	0.15413	-1.78353	-3.75438
C	-1.47629	-4.54725	-2.62794
H	-3.13234	-4.50921	-1.24253
H	0.16304	-4.23966	-3.99071
H	-1.47607	-5.62828	-2.73686
C	-2.60370	0.58550	-2.29601
C	-2.52472	1.96664	-2.03346
C	-3.83974	0.08210	-2.73425
C	-3.62819	2.79499	-2.16961
H	-1.57324	2.38100	-1.70658
C	-4.94794	0.91436	-2.85957
H	-3.93414	-0.97330	-2.97203
C	-4.85993	2.27293	-2.56848
H	-3.52816	3.85595	-1.95142

H	-5.89289	0.48910	-3.18989
H	-5.72993	2.91660	-2.65989

(PPP^{Ph})Ni[η^2(N,C)-N$_2$CPh$_2$] isomer

Energy: -4764.17181408 Hartree

Ni	0.25357	-0.18252	-0.57986
P	-0.04410	-0.71643	1.58869
P	2.04318	0.81703	0.15636
P	-1.58958	1.04655	-0.29583
C	1.60624	-0.72938	2.38928
C	2.58432	0.01658	1.70999
C	3.87120	0.12334	2.24359
C	4.18976	-0.52011	3.43605
C	3.21868	-1.25942	4.10959
C	1.92867	-1.35808	3.59484
C	-0.72306	0.85028	2.29132
C	-1.30519	1.75213	1.37862
C	-1.65796	3.03152	1.80727
C	-1.45806	3.40603	3.13502
C	-0.92574	2.50055	4.04891
C	-0.55216	1.22756	3.62401
C	-0.96131	-2.05228	2.41306
C	-0.40200	-3.33922	2.46782
C	-1.13569	-4.40726	2.97092
C	-2.44200	-4.21140	3.41747
C	-3.00673	-2.93984	3.36124
C	-2.27384	-1.86634	2.86478
C	1.86790	2.58293	0.60674
C	1.37448	3.44844	-0.38051
C	1.09954	4.77919	-0.09006
C	1.29565	5.26340	1.20256
C	1.77188	4.40972	2.19317
C	2.06012	3.07834	1.90023
C	3.53958	0.73619	-0.88168
C	3.96938	1.78671	-1.69707
C	5.04747	1.61030	-2.56062
C	5.70693	0.38667	-2.62045
C	5.28212	-0.66679	-1.81271
C	4.20105	-0.49717	-0.95650
C	-3.16036	0.16901	0.12497
C	-4.13842	0.73647	0.95657
C	-5.28202	0.02318	1.30172
C	-5.46426	-1.27606	0.83060
C	-4.49057	-1.85894	0.02632
C	-3.34623	-1.14423	-0.31850
C	-2.09005	2.45635	-1.35387
C	-3.25282	3.21878	-1.18349
C	-3.54242	4.27191	-2.04581
C	-2.68267	4.57043	-3.10098

C	-1.53776	3.80386	-3.29878
C	-1.24711	2.75242	-2.43396
H	4.62536	0.69973	1.71178
H	5.19558	-0.44635	3.83976
H	3.46724	-1.76249	5.04006
H	1.17278	-1.93423	4.12377
H	-2.07655	3.74283	1.09815
H	-1.71651	4.41247	3.45424
H	-0.77895	2.79199	5.08522
H	-0.09907	0.52939	4.32610
H	0.61372	-3.49820	2.11026
H	-0.68679	-5.39640	3.01168
H	-3.01645	-5.04765	3.80559
H	-4.02587	-2.77818	3.70274
H	-2.72709	-0.87819	2.81473
H	1.19246	3.06131	-1.38150
H	0.71373	5.43160	-0.86943
H	1.91119	4.77617	3.20717
H	2.41649	2.41886	2.68802
H	3.46885	2.74972	-1.65406
H	5.37024	2.43479	-3.19013
H	5.78762	-1.62809	-1.85389
H	3.85850	-1.33046	-0.34508
H	-3.98731	1.73013	1.37350
H	-6.02648	0.47944	1.94897
H	-4.60761	-2.87923	-0.32883
H	-2.57976	-1.61903	-0.92183
H	-3.94437	2.98276	-0.37898
H	-4.44613	4.85671	-1.89725
H	-0.87293	4.01453	-4.13169
H	-0.35974	2.14198	-2.60207
H	6.54656	0.25183	-3.29645
H	1.06678	6.29907	1.43817
H	-2.91395	5.39031	-3.77534
H	-6.35467	-1.83534	1.10532
N	1.29446	0.31114	-3.23344
N	0.80276	-0.35470	-2.38424
C	0.19953	-1.59018	-2.02296
C	1.16393	-2.67442	-1.71457
C	0.84420	-3.64154	-0.74799
C	2.39297	-2.78139	-2.37761
C	1.72120	-4.67611	-0.45102
H	-0.10388	-3.55196	-0.21892
C	3.27758	-3.81267	-2.07040
H	2.66063	-2.03849	-3.12582
C	2.94953	-4.76405	-1.10849
H	1.45025	-5.41108	0.30435
H	4.22846	-3.87507	-2.59452
H	3.64201	-5.56684	-0.87167
C	-1.05641	-1.86276	-2.74002

C	-1.79677	-0.82395	-3.33929
C	-1.58564	-3.16311	-2.82659
C	-3.01434	-1.06781	-3.95785
H	-1.41882	0.19448	-3.30222
C	-2.80781	-3.40078	-3.44736
H	-1.03281	-3.99559	-2.40202
C	-3.53965	-2.35814	-4.00953
H	-3.55924	-0.23628	-4.39788
H	-3.18816	-4.41858	-3.49149
H	-4.49762	-2.54718	-4.48471

(PPP^{Ph})NiCPh$_2$
Energy: -4654.64822810 Hartree

C	1.33100000	-0.86200000	2.64300000
C	2.14000000	0.28800000	2.76000000
C	3.33700000	0.26000000	3.46300000
C	3.78300000	-0.93100000	4.03600000
C	3.01200000	-2.08700000	3.91800000
C	1.79200000	-2.04700000	3.25300000
H	1.79300000	1.20800000	2.29100000
H	3.93000000	1.16700000	3.55200000
H	3.36300000	-3.02100000	4.34900000
H	1.18900000	-2.94800000	3.16500000
C	0.09800000	-0.80800000	1.86000000
C	-1.01900000	-1.58800000	2.36500000
C	-1.31100000	-1.68000000	3.74600000
C	-2.46400000	-2.30800000	4.19800000
C	-3.34400000	-2.91100000	3.29800000
C	-3.05700000	-2.87500000	1.93300000
C	-1.92400000	-2.21600000	1.47700000
H	-0.63700000	-1.20300000	4.45400000
H	-2.67900000	-2.33100000	5.26400000
H	-4.24100000	-3.40800000	3.65900000
H	-3.72600000	-3.34600000	1.21500000
H	-1.71200000	-2.17600000	0.41200000
H	4.73100000	-0.96000000	4.56900000
C	-0.17700000	-3.63000000	-1.15300000
C	-1.01400000	-4.60100000	-1.69100000
C	-1.37300000	-4.54500000	-3.03700000
C	-0.89100000	-3.51000000	-3.83700000
C	-0.05400000	-2.53600000	-3.30000000
C	2.70400000	-2.02500000	-0.41000000
C	2.99300000	-3.39200000	-0.44500000
C	4.15800000	-3.88000000	0.14500000
C	5.04800000	-3.01000000	0.76700000
C	4.77400000	-1.64400000	0.79500000
C	3.61000000	-1.15700000	0.21400000
C	-2.66500000	1.58800000	1.26500000
C	-2.42300000	2.94600000	1.50700000

C	-2.81500000	3.53900000	2.70400000
C	-3.46000000	2.78600000	3.68200000
C	-3.71100000	1.43600000	3.45100000
C	-3.31600000	0.84000000	2.25800000
C	-3.41500000	-0.17200000	-0.88500000
C	-4.74900000	0.10300000	-0.55900000
C	-5.77900000	-0.67200000	-1.08700000
C	-5.49200000	-1.72400000	-1.95400000
C	-4.16900000	-2.00200000	-2.29100000
C	-3.14000000	-1.23600000	-1.75500000
Ni	0.08200000	-0.00300000	0.19000000
P	0.85100000	1.70700000	-0.81800000
P	1.20100000	-1.23500000	-1.10600000
P	-1.97500000	0.74000000	-0.21000000
C	1.83300000	1.16900000	-2.28000000
C	1.96900000	-0.21800000	-2.42900000
H	2.85300000	-1.81300000	-3.57700000
H	3.89500000	-0.28300000	-5.23100000
H	3.65300000	2.17300000	-4.97300000
H	2.37600000	3.10300000	-3.06900000
H	-3.90500000	2.42200000	-1.79400000
H	-3.57600000	4.48500000	-3.12900000
H	-1.30500000	5.45700000	-3.37500000
H	0.64300000	4.33200000	-2.33900000
H	3.78800000	2.05500000	-0.69900000
H	5.21900000	3.48700000	0.71200000
H	4.20000000	5.18900000	2.20900000
H	1.72900000	5.45100000	2.27000000
H	0.29200000	4.00200000	0.87900000
H	0.08300000	-3.66200000	-0.09500000
H	-1.39500000	-5.39500000	-1.05400000
H	-1.17300000	-3.45700000	-4.88500000
H	0.29500000	-1.72100000	-3.92900000
H	2.31500000	-4.08100000	-0.94000000
H	4.36900000	-4.94500000	0.11400000
H	5.45700000	-0.95600000	1.28700000
H	3.38300000	-0.09200000	0.25900000
H	-1.93300000	3.55300000	0.74900000
H	-2.62000000	4.59600000	2.86800000
H	-4.21300000	0.83500000	4.20400000
H	-3.51700000	-0.21700000	2.09600000
H	-4.98000000	0.91700000	0.12300000
H	-6.80900000	-0.45200000	-0.82000000
H	-3.93100000	-2.82400000	-2.96100000
H	-2.10300000	-1.47000000	-1.99300000
C	2.72500000	-0.73600000	-3.48400000
C	3.31600000	0.12200000	-4.40500000
C	3.18000000	1.50300000	-4.26000000
C	2.45100000	2.02500000	-3.19700000
C	-0.52000000	2.70400000	-1.53400000

C	-1.81100000	2.17900000	-1.35500000
C	-2.90500000	2.82700000	-1.93500000
C	-2.72100000	3.99100000	-2.67600000
C	-1.44500000	4.53500000	-2.81700000
C	-0.35000000	3.89600000	-2.24500000
C	1.93700000	2.93800000	-0.02300000
C	3.33300000	2.80100000	-0.05000000
C	4.13900000	3.60700000	0.74800000
C	3.56900000	4.56300000	1.58500000
C	2.18300000	4.70900000	1.61900000
C	1.37500000	3.90200000	0.82700000
H	5.95200000	-3.39400000	1.23200000
H	-2.03100000	-5.30000000	-3.45900000
H	-6.29800000	-2.33000000	-2.36000000
H	-3.76500000	3.24900000	4.61600000
C	0.32100000	-2.58600000	-1.95100000

(dtbpe)NiCPh$_2$
Energy: -3402.74341884 Hartree

Ni	-0.00200	-0.20900	0.12300
P	-0.44900	1.51300	-1.03600
P	-2.03800	-0.88600	0.07100
C	3.95700	-1.78000	0.62700
H	4.06300	-1.45200	1.65900
C	1.90400	-0.05100	1.81300
C	1.65000	-0.78500	0.56600
C	-0.15600	3.19400	-0.21000
C	3.68500	-2.63200	-2.00700
H	3.57700	-2.95900	-3.03800
C	1.11500	-0.26900	2.96100
H	0.42500	-1.10900	2.95200
C	0.33400	1.54100	-2.76400
C	-2.28000	1.49700	-1.39700
H	-2.74800	2.12600	-0.63200
H	-2.52000	1.95900	-2.36400
C	1.84900	1.33700	-2.65200
H	2.25000	1.02700	-3.62500
H	2.09400	0.56100	-1.92100
H	2.37500	2.24800	-2.36100
C	-2.18400	-2.67600	-0.54400
C	-3.22300	-0.65700	1.53700
C	-0.24600	0.32200	-3.48700
H	0.25200	0.19900	-4.45600
H	-1.31900	0.42300	-3.68400
H	-0.08500	-0.59500	-2.90800
C	-2.84900	0.07700	-1.30700
H	-2.62600	-0.47100	-2.23000
H	-3.94300	0.09000	-1.21200
C	2.75700	-1.48300	-0.05800

C	0.03500	2.78700	-3.59300
H	0.39500	2.63900	-4.62000
H	0.54000	3.67500	-3.20300
H	-1.03700	3.00300	-3.65600
C	2.65500	-1.94000	-1.39100
H	1.73700	-1.71700	-1.93400
C	4.85800	-2.91500	-1.30400
H	5.66300	-3.46600	-1.78100
C	4.98400	-2.48900	0.01800
H	5.89100	-2.71100	0.57400
C	-3.52000	-3.04900	-1.18900
H	-3.43500	-4.04900	-1.63300
H	-3.79100	-2.36500	-2.00100
H	-4.35200	-3.08200	-0.48500
C	2.85300	0.99500	1.87500
H	3.49300	1.16400	1.01000
C	1.22200	0.54900	4.08200
H	0.59400	0.35400	4.94900
C	-0.99700	4.34000	-0.77400
H	-0.78200	5.25600	-0.20800
H	-2.07100	4.15400	-0.67800
H	-0.78500	4.55200	-1.82400
C	-1.86400	-3.61900	0.61600
H	-1.76600	-4.64500	0.24100
H	-2.64600	-3.62900	1.38200
H	-0.91500	-3.34700	1.09300
C	-0.50100	3.03100	1.27300
H	-0.18400	3.92600	1.82300
H	-0.00400	2.16400	1.72600
H	-1.57900	2.92900	1.42400
C	1.32800	3.54300	-0.30900
H	1.55300	4.37600	0.36800
H	1.61400	3.85700	-1.31700
H	1.96200	2.70000	-0.01300
C	-1.09000	-2.85300	-1.59900
H	-1.05000	-3.90300	-1.91500
H	-0.11000	-2.57100	-1.19700
H	-1.28300	-2.25000	-2.49300
C	2.12100	1.61300	4.09500
H	2.19300	2.26400	4.96200
C	2.94200	1.82500	2.98500
H	3.65900	2.64300	2.98800
C	-3.55800	0.83200	1.61300
H	-4.11300	1.03500	2.53700
H	-4.18300	1.16500	0.77800
H	-2.64800	1.43400	1.63400
C	-2.48100	-1.02200	2.82400
H	-3.15500	-0.90800	3.68200
H	-1.62500	-0.35700	2.97700
H	-2.10900	-2.05100	2.82700

C	-4.53800	-1.42700	1.45200
H	-5.18700	-1.12100	2.28200
H	-4.40300	-2.50900	1.53400
H	-5.08100	-1.21800	0.52400

(PCP)NiPPh$_3$
Energy: -4202.11971675 Hartree

Ni	-0.42176	-0.03180	-0.11583
P	-0.36502	-2.06606	-0.91370
P	-1.43311	1.91591	-0.36779
P	1.68073	0.42958	0.26770
C	-2.02066	-0.63187	0.66592
C	-2.09628	-2.02548	1.07044
C	-1.27584	-2.95325	0.37246
C	-1.22056	-4.29529	0.73627
H	-0.60476	-4.99151	0.17237
C	-1.93666	-4.75670	1.84068
H	-1.87597	-5.79888	2.13862
C	-2.71755	-3.85276	2.56354
H	-3.25922	-4.19344	3.44323
C	-2.81929	-2.52197	2.18179
H	-3.41324	-1.83678	2.77997
C	-3.24867	0.15136	0.56693
C	-3.14215	1.48141	0.07844
C	-4.25668	2.29619	-0.07277
H	-4.14556	3.30028	-0.47955
C	-5.53107	1.82540	0.25626
H	-6.40248	2.46096	0.13008
C	-5.66315	0.51621	0.71268
H	-6.65106	0.12072	0.93791
C	-4.55403	-0.31039	0.85090
H	-4.70389	-1.34245	1.15260
C	1.15172	-3.07890	-1.33468
H	1.75856	-2.39489	-1.94292
C	0.90192	-4.33282	-2.17195
H	1.84952	-4.86226	-2.32245
H	0.49858	-4.10983	-3.16281
H	0.21468	-5.03378	-1.68854
C	1.95588	-3.41415	-0.08544
H	2.90080	-3.89278	-0.36595
H	1.41693	-4.09531	0.57998
H	2.19957	-2.52385	0.49577
C	-1.36763	-2.24448	-2.47732
H	-1.44769	-3.32407	-2.66613
C	-2.77110	-1.68377	-2.31640
H	-3.35231	-1.85786	-3.22886
H	-2.74971	-0.60584	-2.12651
H	-3.30246	-2.14601	-1.47893
C	-0.62170	-1.58100	-3.62882

H	-1.20305	-1.63614	-4.55586
H	0.35766	-2.03368	-3.81863
H	-0.44583	-0.52128	-3.40396
C	-0.97921	3.23865	0.87135
H	0.05645	3.49943	0.61886
C	-1.81397	4.51060	0.82818
H	-1.43487	5.22457	1.56858
H	-2.86140	4.31125	1.07732
H	-1.78217	5.00976	-0.14540
C	-1.00076	2.62677	2.26845
H	-0.64392	3.35091	3.00988
H	-0.37589	1.72913	2.33570
H	-2.01852	2.32905	2.54530
C	-1.64580	2.87321	-1.95060
H	-2.34733	3.69246	-1.73665
C	-2.24847	1.99679	-3.03618
H	-2.37627	2.56472	-3.96415
H	-3.22433	1.59668	-2.74527
H	-1.59059	1.14793	-3.25454
C	-0.31760	3.46581	-2.39531
H	-0.43508	4.03211	-3.32580
H	0.41037	2.66509	-2.58205
H	0.12015	4.13398	-1.64624
C	2.25215	2.15383	0.54914
C	2.25299	2.70973	1.83583
H	2.01910	2.08568	2.69412
C	2.52722	4.06110	2.02633
H	2.51322	4.47405	3.03151
C	2.81244	4.88026	0.93698
H	3.02256	5.93555	1.08571
C	2.83471	4.33437	-0.34461
H	3.06727	4.96114	-1.20157
C	2.55681	2.98432	-0.53582
H	2.57522	2.57173	-1.54179
C	2.83271	-0.13928	-1.04866
C	2.43008	0.10968	-2.37027
H	1.47951	0.61811	-2.54328
C	3.19222	-0.33263	-3.44635
H	2.85950	-0.13175	-4.46143
C	4.36081	-1.05703	-3.21890
H	4.94868	-1.42243	-4.05614
C	4.76448	-1.32118	-1.91283
H	5.66829	-1.89563	-1.72842
C	4.01249	-0.85808	-0.83488
H	4.33117	-1.09123	0.17760
C	2.22939	-0.37050	1.82923
C	3.50359	-0.18809	2.38414
H	4.21720	0.47149	1.89378
C	3.85317	-0.82687	3.57027
H	4.84409	-0.67961	3.99117

C	2.93251	-1.64805	4.22017
H	3.20951	-2.14566	5.14556
C	1.65755	-1.82050	3.68794
H	0.92882	-2.45180	4.18919
C	1.30761	-1.18144	2.50234
H	0.30904	-1.30456	2.08004

(PPP^{Ph})$NiCPh_2$ no central P

Energy: -4654.60858573 Hartree

Ni	-0.51182	0.16388	-0.31003
P	1.92658	-0.81807	-2.72447
P	1.22364	1.44536	-0.03770
P	0.06459	-1.84093	0.17636
C	2.73056	0.81178	-2.41297
C	2.50126	1.72847	-1.36367
C	3.27737	2.89380	-1.31445
C	4.25717	3.16765	-2.26135
C	4.47765	2.27264	-3.30078
C	3.71600	1.11257	-3.36831
C	2.48503	-1.84350	-1.28717
C	1.77541	-2.38319	-0.19505
C	2.43114	-3.29181	0.65348
C	3.77298	-3.60783	0.49455
C	4.48995	-3.03574	-0.55417
C	3.83710	-2.19929	-1.44755
C	0.13361	-0.48801	-2.77985
C	-0.40209	0.82213	-2.82882
C	-1.65142	1.05880	-3.41039
C	-2.39848	0.01248	-3.93074
C	-1.90658	-1.29349	-3.85239
C	-0.65909	-1.53542	-3.30307
C	2.32897	1.11031	1.38757
C	1.99715	1.50019	2.69394
C	2.78147	1.10778	3.77480
C	3.90828	0.31399	3.57542
C	4.25638	-0.06918	2.28148
C	3.47875	0.32963	1.19900
C	0.65140	3.17690	0.26218
C	1.32607	4.11240	1.05837
C	0.83555	5.40750	1.20568
C	-0.32757	5.79609	0.54668
C	-1.00317	4.87896	-0.25428
C	-0.52196	3.58058	-0.38680
C	-0.96553	-3.15912	-0.57178
C	-0.42616	-4.30765	-1.16088
C	-1.25358	-5.22931	-1.79919
C	-2.62999	-5.02487	-1.84661
C	-3.17952	-3.89261	-1.24801
C	-2.35248	-2.96472	-0.62560

C	-0.10244	-2.14877	1.97669
C	-1.29440	-2.64610	2.51760
C	-1.45755	-2.75935	3.89471
C	-0.43505	-2.37825	4.75826
C	0.74995	-1.86838	4.23254
C	0.91089	-1.74246	2.85735
C	-3.30900	1.12254	-0.45238
C	-3.98009	0.31759	-1.38552
C	-5.07584	0.79773	-2.09682
C	-5.50428	2.11268	-1.93775
C	-4.84296	2.93553	-1.02488
C	-3.78768	2.43931	-0.27143
H	3.11093	3.61044	-0.51601
H	4.84163	4.08034	-2.18393
H	5.23197	2.47423	-4.05598
H	3.87518	0.40697	-4.18082
H	1.87328	-3.74129	1.47072
H	4.25227	-4.30227	1.17892
H	5.54275	-3.26461	-0.69487
H	4.38151	-1.80209	-2.30400
H	0.22127	1.67359	-2.57098
H	-2.03665	2.07556	-3.44819
H	-3.36444	0.21001	-4.38527
H	-2.48464	-2.12324	-4.25106
H	-0.26215	-2.54830	-3.31165
H	1.12357	2.12239	2.86747
H	2.50530	1.42307	4.77796
H	5.13759	-0.68154	2.10907
H	3.76583	0.02630	0.19560
H	2.23836	3.82594	1.57600
H	1.36613	6.11353	1.83884
H	-1.91797	5.16422	-0.76784
H	-1.08028	2.84980	-0.96935
H	0.64768	-4.47666	-1.13610
H	-0.81812	-6.11110	-2.26153
H	-4.25247	-3.72083	-1.27917
H	-2.77531	-2.06371	-0.18260
H	-2.10351	-2.95319	1.86147
H	-2.39313	-3.14378	4.29188
H	1.55651	-1.55679	4.89221
H	1.84217	-1.34464	2.46270
H	-3.62651	-0.69608	-1.55593
H	-5.58635	0.14017	-2.79729
H	-5.17078	3.96241	-0.87928
H	-3.30015	3.07116	0.46860
C	-2.15571	0.66217	0.33000
C	-2.41941	0.68340	1.77286
C	-3.72306	0.60004	2.31371
C	-3.93631	0.57866	3.68662
C	-2.86190	0.67404	4.56988

C	-1.56501	0.76279	4.06297
C	-1.35561	0.74431	2.69465
H	-4.57137	0.53564	1.63683
H	-4.94939	0.49662	4.07256
H	-3.03296	0.67071	5.64328
H	-0.71239	0.81939	4.73671
H	-0.34898	0.76656	2.29178
H	-0.70920	6.80653	0.66365
H	4.51567	0.00321	4.42097
H	-0.56407	-2.46867	5.83342
H	-3.27145	-5.74346	-2.34908
H	-6.34556	2.49114	-2.51139

(PPP^{Ph})NiCPh$_2$ no side P
Energy: -4654.62037343 Hartree

Ni	2.15959	0.63192	0.05783
P	0.11030	0.54289	-0.41358
P	2.23328	-1.47624	-0.24341
P	-3.36724	0.05263	-0.45293
C	-0.04190	-0.81295	-1.64390
C	0.97290	-1.78648	-1.53858
C	0.97333	-2.89003	-2.39200
C	-0.01559	-3.02186	-3.36415
C	-0.99941	-2.04534	-3.49275
C	-1.01460	-0.94611	-2.63739
C	-0.94740	0.11946	1.04769
C	-2.34843	-0.06479	1.08052
C	-2.95811	-0.38903	2.30008
C	-2.21828	-0.53260	3.46961
C	-0.84136	-0.34558	3.43995
C	-0.22133	-0.02685	2.23828
C	-0.68460	2.00472	-1.13506
C	-0.61059	2.27279	-2.50626
C	-1.13089	3.45553	-3.02213
C	-1.69826	4.40219	-2.17375
C	-1.73715	4.16217	-0.80135
C	-1.23663	2.97275	-0.28455
C	1.68724	-2.53464	1.14522
C	2.47044	-2.55244	2.30704
C	2.02633	-3.20218	3.45242
C	0.78633	-3.84113	3.45712
C	0.00440	-3.83664	2.30564
C	0.45236	-3.19152	1.15506
C	3.72324	-2.33302	-0.87506
C	3.98694	-3.68820	-0.64328
C	5.13118	-4.28088	-1.17038
C	6.01989	-3.52701	-1.93363
C	5.76645	-2.17655	-2.16531
C	4.62743	-1.58076	-1.63374

C	-3.60999	-1.72178	-0.85298
C	-2.92478	-2.75820	-0.20876
C	-3.01829	-4.06897	-0.67131
C	-3.81627	-4.37101	-1.77068
C	-4.51882	-3.35074	-2.41069
C	-4.40366	-2.03835	-1.96638
C	-4.99372	0.55417	0.23109
C	-6.06548	-0.31625	0.46059
C	-7.27242	0.16600	0.96212
C	-7.42255	1.51958	1.24998
C	-6.36111	2.39503	1.02702
C	-5.16104	1.91822	0.51269
C	2.72874	3.59637	-0.46304
C	2.34661	3.69127	-1.81831
C	2.21766	4.91782	-2.44951
C	2.50835	6.09569	-1.75865
C	2.92717	6.02901	-0.42966
C	3.02814	4.80203	0.21118
H	1.75865	-3.63820	-2.30395
H	-0.01152	-3.88188	-4.02811
H	-1.76718	-2.14145	-4.25561
H	-1.79043	-0.19105	-2.73536
H	-4.03519	-0.53948	2.32704
H	-2.72108	-0.79110	4.39782
H	-0.24030	-0.45079	4.33940
H	0.85850	0.10819	2.20689
H	-0.14234	1.55034	-3.17103
H	-1.08127	3.64290	-4.09160
H	-2.09601	5.32876	-2.57736
H	-2.16203	4.90407	-0.13018
H	-1.27839	2.78481	0.78733
H	3.43081	-2.03908	2.30863
H	2.64480	-3.20370	4.34644
H	-0.96465	-4.33094	2.30234
H	-0.16941	-3.18526	0.26252
H	3.29513	-4.27647	-0.04335
H	5.33008	-5.33274	-0.98328
H	6.46253	-1.58412	-2.75256
H	4.43186	-0.51924	-1.78592
H	-2.30816	-2.53613	0.65869
H	-2.46459	-4.85686	-0.16551
H	-5.14686	-3.57649	-3.26845
H	-4.93279	-1.24324	-2.49010
H	-5.95265	-1.37691	0.24854
H	-8.09590	-0.52191	1.13482
H	-6.47270	3.45369	1.24494
H	-4.33830	2.60654	0.32174
H	2.14440	2.76373	-2.35047
H	1.89726	4.96118	-3.48782
H	3.16953	6.94197	0.10880

H	3.35288	4.75538	1.24837
C	2.82580	2.30136	0.15529
C	2.75289	2.13764	1.60492
C	1.83910	2.85311	2.42909
C	1.60614	2.47834	3.73959
C	2.28808	1.38948	4.30152
C	3.21020	0.68878	3.53487
C	3.44935	1.05377	2.20629
H	1.28414	3.67786	1.98635
H	0.87852	3.02405	4.33591
H	2.10274	1.10371	5.33362
H	3.75949	-0.14181	3.97231
H	4.23808	0.56822	1.63629
H	6.91408	-3.99057	-2.34120
H	0.43226	-4.33806	4.35592
H	-8.36410	1.89270	1.64294
H	-3.88998	-5.39366	-2.12948
H	2.42230	7.05765	-2.25682

CPh_2
Energy: -501.395950957 Hartree

C	-1.22427	0.57922	0.02979
C	-2.37180	1.14249	-0.58625
C	-3.58863	0.48512	-0.56734
C	-3.72680	-0.70855	0.14935
C	-2.63004	-1.26028	0.81252
C	-1.38747	-0.65044	0.72457
H	-2.25488	2.09758	-1.09099
H	-4.44841	0.91424	-1.07421
H	-2.74670	-2.18087	1.37828
H	-0.52461	-1.08413	1.22411
C	-0.00000	1.30888	-0.00001
C	1.22427	0.57922	-0.02970
C	1.38742	-0.65048	-0.72445
C	2.62999	-1.26032	-0.81249
C	3.72680	-0.70857	-0.14945
C	3.58868	0.48512	0.56724
C	2.37185	1.14248	0.58625
H	0.52453	-1.08419	-1.22391
H	2.74658	-2.18094	-1.37823
H	4.69517	-1.19839	-0.20381
H	4.44849	0.91425	1.07403
H	2.25500	2.09755	1.09105
H	-4.69516	-1.19840	0.20360

(dtbpe)Ni
Energy: -2901.21489372 Hartree

Ni	0.00008	0.00002	-1.34079

P	-1.61269	0.00095	-0.07998
P	1.61280	-0.00099	-0.07992
C	-2.88197	1.41568	0.01093
C	-2.53667	-1.64135	0.17305
C	-0.72754	0.24683	1.55485
H	-0.74064	1.32814	1.73336
H	-1.28631	-0.20329	2.38824
C	-3.03697	-2.11739	-1.19224
H	-3.49758	-3.10944	-1.09867
H	-2.20685	-2.19658	-1.90382
H	-3.78097	-1.44609	-1.62870
C	2.88199	-1.41580	0.01095
C	2.53668	1.64149	0.17306
C	-1.49153	-2.64777	0.65626
H	-1.92559	-3.65555	0.67041
H	-1.14127	-2.42956	1.67098
H	-0.62312	-2.66375	-0.01149
C	0.72761	-0.24690	1.55483
H	0.74061	-1.32825	1.73314
H	1.28635	0.20304	2.38833
C	-3.68888	-1.63207	1.17210
H	-4.04499	-2.65817	1.33420
H	-4.54510	-1.04985	0.81930
H	-3.38680	-1.23679	2.14851
C	3.55601	-1.64945	1.36487
H	4.16969	-2.55873	1.31381
H	2.82820	-1.80351	2.16793
H	4.21729	-0.83482	1.66325
C	-3.55595	1.64938	1.36487
H	-4.16885	2.55920	1.31408
H	-2.82820	1.80244	2.16817
H	-4.21800	0.83516	1.66270
C	3.94387	-1.19066	-1.06312
H	4.56027	-2.09094	-1.17908
H	4.62016	-0.36758	-0.81205
H	3.48722	-0.96968	-2.03531
C	-2.11197	2.69124	-0.34113
H	-2.81072	3.53258	-0.43332
H	-1.56636	2.59386	-1.28801
H	-1.38796	2.95568	0.43614
C	-3.94395	1.19023	-1.06298
H	-4.56065	2.09033	-1.17889
H	-4.61995	0.36697	-0.81169
H	-3.48745	0.96932	-2.03526
C	2.11195	-2.69139	-0.34089
H	2.81066	-3.53278	-0.43288
H	1.56641	-2.59412	-1.28783
H	1.38786	-2.95564	0.43638
C	1.49136	2.64776	0.65624
H	1.92504	3.65572	0.66977

H	1.14163	2.42989	1.67120
H	0.62265	2.66312	-0.01114
C	3.03673	2.11755	-1.19230
H	3.49726	3.10966	-1.09881
H	2.20649	2.19668	-1.90374
H	3.78069	1.44633	-1.62890
C	3.68891	1.63262	1.17207
H	4.04487	2.65885	1.33381
H	4.54526	1.05041	0.81956
H	3.38684	1.23769	2.14863

(PPP^{Ph})Ni
Energy: -4153.14137797 Hartree

Ni	0.09296	-0.08374	-1.08258
P	-0.38370	-1.61863	0.23105
P	2.09083	0.03269	-0.48182
P	-1.54937	1.11348	-0.51156
C	1.18803	-2.13612	1.03890
C	2.31812	-1.35701	0.73597
C	3.53941	-1.65812	1.34303
C	3.64966	-2.73705	2.21545
C	2.53443	-3.52453	2.49528
C	1.30737	-3.22111	1.91370
C	-1.23771	-0.70756	1.58583
C	-1.74341	0.56654	1.25082
C	-2.30079	1.36323	2.25405
C	-2.35495	0.91035	3.57000
C	-1.85939	-0.34885	3.89955
C	-1.30341	-1.15319	2.90852
C	-1.28412	-3.20122	0.09593
C	-0.74056	-4.16189	-0.77083
C	-1.39424	-5.36481	-1.00084
C	-2.61731	-5.62664	-0.38223
C	-3.16943	-4.68128	0.47667
C	-2.50423	-3.48172	0.72266
C	2.38754	1.47814	0.60537
C	3.01026	2.64463	0.14117
C	3.03071	3.79619	0.92344
C	2.40906	3.81108	2.17058
C	1.77486	2.66047	2.63593
C	1.77017	1.50364	1.86486
C	3.62575	-0.09893	-1.48184
C	4.88652	0.35316	-1.06948
C	6.00467	0.17712	-1.87936
C	5.88298	-0.46104	-3.11152
C	4.63680	-0.91793	-3.53303
C	3.51895	-0.73224	-2.72588
C	-3.15131	0.67635	-1.28298
C	-3.82873	-0.48331	-0.89065

C	-4.95614	-0.91621	-1.58029
C	-5.42345	-0.19633	-2.67756
C	-4.75102	0.95503	-3.08330
C	-3.62008	1.38563	-2.39681
C	-1.65451	2.93797	-0.34225
C	-2.85796	3.64803	-0.23232
C	-2.85034	5.03045	-0.06873
C	-1.64121	5.71992	0.00118
C	-0.44001	5.02257	-0.10018
C	-0.44831	3.64282	-0.28003
H	4.41754	-1.05693	1.11739
H	4.60946	-2.97157	2.66778
H	2.62257	-4.37692	3.16358
H	0.43732	-3.84237	2.12098
H	-2.67633	2.35396	2.01016
H	-2.78000	1.54659	4.34171
H	-1.89580	-0.69924	4.92749
H	-0.89587	-2.12938	3.16524
H	0.20614	-3.95094	-1.26704
H	-0.95458	-6.09953	-1.67009
H	-3.13511	-6.56288	-0.57025
H	-4.11806	-4.88018	0.96875
H	-2.93644	-2.75882	1.41169
H	3.46788	2.65435	-0.84566
H	3.52491	4.69008	0.55062
H	1.27685	2.66175	3.60216
H	1.26337	0.61165	2.23214
H	4.99049	0.85911	-0.11188
H	6.97419	0.53814	-1.54642
H	4.53390	-1.41275	-4.49484
H	2.54163	-1.07919	-3.05922
H	-3.45938	-1.05466	-0.04196
H	-5.46602	-1.82140	-1.25989
H	-5.10668	1.51883	-3.94171
H	-3.09589	2.28229	-2.72164
H	-3.80488	3.11393	-0.27936
H	-3.79056	5.57034	0.00823
H	0.50851	5.55099	-0.04609
H	0.48718	3.09494	-0.37956
H	6.75628	-0.59719	-3.74332
H	2.41305	4.71549	2.77247
H	-1.63721	6.79890	0.13037
H	-6.30384	-0.53313	-3.21759

(PPP^{Ph})Ni(*p*-anisyl)$_2$
Energy: -4883.75890512 Hartree

Ni	0.00687	-0.19134	-0.00836
P	0.85789	-1.85294	-1.02631
P	0.95679	-0.82373	1.76712

P	-1.99495	-0.84179	-0.69615
C	1.70881	-2.94697	0.18600
C	1.73826	-2.46590	1.50227
C	2.39975	-3.19239	2.49677
C	3.00016	-4.40931	2.18873
C	2.96644	-4.89298	0.88104
C	2.33292	-4.16064	-0.11853
C	-0.46459	-2.90822	-1.75099
C	-1.78269	-2.49792	-1.48689
C	-2.85026	-3.28133	-1.93208
C	-2.61343	-4.45613	-2.63993
C	-1.30735	-4.84140	-2.93821
C	-0.23726	-4.06760	-2.49904
C	2.08622	-1.69842	-2.36811
C	3.46443	-1.71504	-2.10664
C	4.37849	-1.36512	-3.09604
C	3.93579	-0.99158	-4.36293
C	2.56785	-0.97084	-4.63412
C	1.65317	-1.31502	-3.64660
C	-0.06068	-0.99388	3.26892
C	-0.59904	0.17978	3.82373
C	-1.52825	0.11901	4.85552
C	-1.94318	-1.11662	5.35041
C	-1.42504	-2.28763	4.80124
C	-0.49335	-2.22978	3.76786
C	2.42511	0.13586	2.31202
C	2.58888	0.73510	3.56402
C	3.72851	1.48817	3.84206
C	4.72059	1.64464	2.87936
C	4.57421	1.03470	1.63498
C	3.43623	0.29059	1.35354
C	-2.53889	0.13681	-2.15083
C	-2.17042	-0.22099	-3.45471
C	-2.43765	0.62404	-4.52814
C	-3.07466	1.84445	-4.32032
C	-3.45138	2.20853	-3.02980
C	-3.18760	1.36530	-1.95603
C	-3.53363	-1.03307	0.28303
C	-4.82400	-0.84011	-0.22624
C	-5.93593	-0.96003	0.60370
C	-5.77569	-1.28245	1.94924
C	-4.49669	-1.48425	2.46475
C	-3.38588	-1.35296	1.63877
C	1.31042	2.37700	-0.19777
C	2.24169	1.93206	-1.15699
C	3.48004	2.53590	-1.32320
C	3.84567	3.58977	-0.47862
C	2.94490	4.05723	0.48767
C	1.69505	3.47931	0.60148
H	2.44545	-2.79877	3.51069

H	3.50517	-4.97717	2.96528
H	3.44464	-5.83819	0.63915
H	2.33871	-4.52596	-1.14308
H	-3.87112	-2.96195	-1.73059
H	-3.44928	-5.06366	-2.97620
H	-1.12348	-5.74591	-3.51160
H	0.78110	-4.35789	-2.75006
H	3.82237	-2.00378	-1.12032
H	5.44273	-1.38780	-2.87529
H	4.65020	-0.71749	-5.13393
H	2.21198	-0.67902	-5.61857
H	0.58558	-1.26836	-3.85635
H	-0.29785	1.14563	3.42049
H	-1.93612	1.03878	5.26616
H	-1.75019	-3.25466	5.17599
H	-0.11367	-3.15110	3.33351
H	1.82991	0.61150	4.33151
H	3.83874	1.95239	4.81855
H	5.34072	1.15479	0.87295
H	3.31202	-0.16398	0.36980
H	-1.67923	-1.17456	-3.63622
H	-2.14647	0.32425	-5.53168
H	-3.94278	3.16194	-2.84853
H	-3.48757	1.66495	-0.95498
H	-4.95680	-0.57901	-1.27374
H	-6.93112	-0.80057	0.19674
H	-4.35800	-1.72990	3.51467
H	-2.38335	-1.47868	2.04666
H	1.96104	1.09231	-1.79149
H	4.15597	2.16922	-2.08907
H	3.25881	4.86940	1.13656
H	1.00033	3.84616	1.35415
C	0.05500	1.66674	-0.01781
C	-1.10311	2.46630	0.34992
C	-1.35344	3.71327	-0.25074
C	-2.55537	4.38915	-0.03632
C	-3.51384	3.87496	0.84362
C	-3.24860	2.66369	1.48997
C	-2.08313	1.96109	1.23929
H	-0.64025	4.13609	-0.95289
H	-4.44690	4.39632	1.02628
H	-3.98414	2.25287	2.17895
H	-1.90846	1.00240	1.71820
H	5.60284	2.24124	3.09420
H	-2.67348	-1.16611	6.15332
H	-6.64487	-1.36974	2.59562
H	-3.27589	2.50659	-5.15781
O	5.05450	4.21501	-0.51026
O	-2.70971	5.55008	-0.74110
C	5.98223	3.76561	-1.47478

H	5.60065	3.89235	-2.49614
H	6.87617	4.37581	-1.34606
H	6.24128	2.70811	-1.32923
C	-3.91749	6.25156	-0.55521
H	-3.86220	7.13223	-1.19558
H	-4.78938	5.64848	-0.84559
H	-4.05010	6.57206	0.48715

REFERENCES:

(1) Purification of Laboratory Chemicals; Armarego, W. L. F., Chai, C. L. L., Eds.; Elsevier Science: Oxford, UK, 2003.
(2) Pérez-García, P.M.; Darù, A.; Scheerder, A.R.; Lutz, M.; Harvey, J.N.; Moret, M-E. *Organometallics* **2020**, *39*, 1139−1144.
(3) Davis, P. J.; Harris, L.; Karim, A.; Thompson, A. L.; Gilpin, M.; Moloney, M. G.; Pound, M. J.; Thompson, C. *Tetrahedron Lett.* **2011**, *52* (14), 1553–1556.
(4) Brookhart, M.; Grant B.; Volpe, A. F. Jr. *Organometallics* **1992**, *11*, 3920-3922.
(5) Waterman, R.; Hillhouse, G. L. *Can. J. Chem.* **2005**, *83*, 328-331.
(6) Kim, Y.-E.; Kim, J.; Lee, Y. *Chem. Commun.* **2014**, *50*, 11458-11461.
(7) Melzer, A; Jenny, E. F. *Tetrahedron Letters* **1968**, *43*, 4503-4506.
(8) Mindiola, D. J.; Hillhouse, G. L. *J. Am. Chem. Soc.* **2002**, *34*, 9976-9977.
(9) Moore, D. L.; Denton, A. E.; Kohinke, R. M.; Craig, B. R.; Brenzovich, W. E. Jr. *Synthetic Communications* **2016**, *46*, 604-612.
(10) Stoll, S.; Schweiger, A. *J. Magn. Reson.* **2006**, *178*, 42-55.
(11) Frisch, M. J.; Trucks, G. W.; Schlegel, H. B.; Scuseria, G. E.; Robb, M. a.; Cheeseman, J. R.; Scalmani, G.; Barone, V.; Petersson, G. a.; Nakatsuji, H.; et al. G16_C01. 2016, p Gaussian 16, Revision C.01, Gaussian, Inc., Wallin.
(12) a) Zha, Y.; Truhlar, D. G. *J. Chem. Phys.* **2006**, *125*, 194101; b) Rassolov, V. A.; Pople, J. A.; Ratner, M. A.; Windus, T. L. *J. Chem. Phys.* **1998**, *109*, 1223-1229.
(13) Lu, T.; Chen, F. *J. Comput. Chem.* **2012**, *33*, 580–592.
(14) Becke, A. D. *Phys. Rev. A* **1988**, *38*, 3098; b) Lee, C.; Yang, W.; Parr, R. G. *Phys. Rev. B* **1988**, *37*, 785; c) Grimme, S.; Antony, J.; Ehrlich, S.; Krieg, H. *J. Chem. Phys.* **2010**, *132*, 154104; d) Grimme, S.; Ehrlich, S.; Goerigk, L. *J. Comput. Chem.* **2011**, *32*, 1456; e) Johnson, E. R.; Becke, A. D. *J. Chem. Phys.* **2005**, *123*, 024101; f) van Lenthe, E.; Baerends, E. J.; Snijders, J. G. *J. Chem. Phys.* **1993**, *99*, 4597; g) van Lenthe, E.; Baerends, E. J.; Snijders, J. G. *J. Chem. Phys.* **1994**, *101*, 9783; h) van Lenthe, E.; Ehlers, A.; Baerends, E. J. *J. Chem. Phys.* **1999**, *110*, 8943.
(15) a) te Velde, G.; Bickelhaupt, F. M.; Baerends, E. J.; Fonseca Guerra, C.; van Gisbergen, S. J. A.; Snijders, J. G. ; Ziegler, T. *J. Comput. Chem.* **2001**, *22*, 931; b) Fonseca Guerra, C.; Snijders, J. G.; te Velde, G.; Baerends, E. J. *Theor. Chem. Acc.* **1998**, *99*, 391; c) ADF2022, SCM Theoretical Chemistry, Vrije Universiteit, Amsterdam, The Netherlands, www.scm.com.
(16) G. Frenking, M. Solà, S. F. Vyboishchikov. *J. Organomet. Chem.* **2005**, *690*, 6178-6204.
(17) Gutsulyak, D. V.; Piers, W. E.; Borau-Garcia, J.; Parvez, M. *J. Am. Chem. Soc.* **2013**, *135*, 11776-11779.
